# Computational resources to define alleles and altered regulatory motifs at genomically edited candidate response elements

**DOI:** 10.1093/nar/gkab700

**Published:** 2021-08-20

**Authors:** Kirk T Ehmsen, Matthew T Knuesel, Delsy Martinez, Masako Asahina, Haruna Aridomi, Keith R Yamamoto

**Affiliations:** Department of Cellular and Molecular Pharmacology, University of California at San Francisco, 600 16th Street, GH S572D, Box 2280, San Francisco, CA 94143-2280, USA; Department of Cellular and Molecular Pharmacology, University of California at San Francisco, 600 16th Street, GH S572D, Box 2280, San Francisco, CA 94143-2280, USA; Department of Cellular and Molecular Pharmacology, University of California at San Francisco, 600 16th Street, GH S572D, Box 2280, San Francisco, CA 94143-2280, USA; Department of Cellular and Molecular Pharmacology, University of California at San Francisco, 600 16th Street, GH S572D, Box 2280, San Francisco, CA 94143-2280, USA; Department of Cellular and Molecular Pharmacology, University of California at San Francisco, 600 16th Street, GH S572D, Box 2280, San Francisco, CA 94143-2280, USA; Department of Cellular and Molecular Pharmacology, University of California at San Francisco, 600 16th Street, GH S572D, Box 2280, San Francisco, CA 94143-2280, USA

## Abstract

Unequivocal functional assessment of candidate genomic regulatory regions, such as transcriptional response elements, requires genetic alteration at their native chromosomal loci. Targeted DNA cleavage by Cas9 or other programmable nucleases enables analysis at virtually any genomic region, and diverse alleles generated by editing can be defined by deep sequencing for functional analysis. Interpretation of disrupted response elements, however, presents a special challenge, as these regions typically comprise clustered DNA binding motifs for multiple transcriptional regulatory factors (TFs); DNA sequence differences, natural or engineered, that affect binding by one TF can confer loss or gain of binding sites for other TFs. To address these and other analytical complexities, we created three computational tools that together integrate, in a single experiment, allele definition and TF binding motif evaluation for up to 9216 clones isolated, sequenced and propagated from Cas9-treated cell populations. We demonstrate 1) the capacity to functionally assess edited TF binding sites to query response element function, and 2) the efficacy and utility of these tools, by analyzing cell populations targeted by Cas9 for disruption of example glucocorticoid receptor (GR) binding motifs near *FKBP5*, a GR-regulated gene in the human adenocarcinoma cell line A549.

## Graphical Abstract

**Graphical Abstract:**
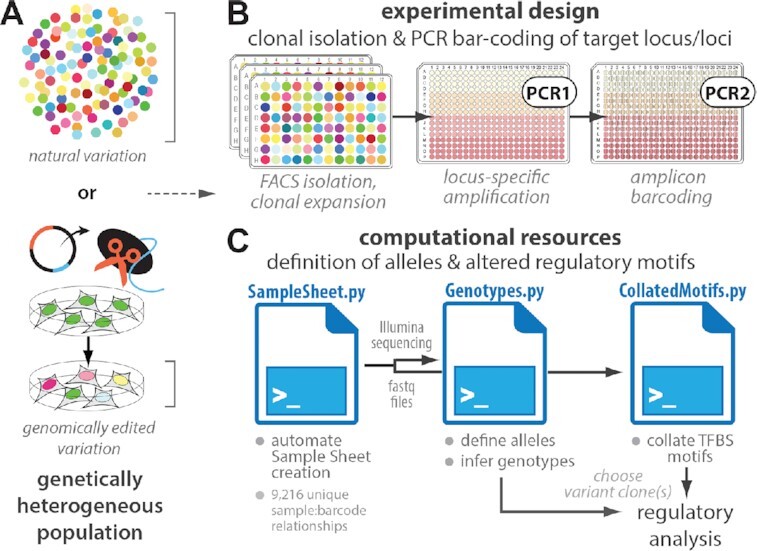
Workflow to evaluate (A) a genetically heterogeneous sample population includes (B) clonal isolation and PCR-barcoding of one or more target loci from diverse samples, followed by (C) computational resources to define alleles and altered transcription factor binding sites (TFBSs) that inform clone selection and regulatory analysis.

## INTRODUCTION

The glucocorticoid receptor (GR; product of the *NR3C1* gene) (1) is a ligand-gated transcriptional regulatory factor (TF) that binds to specific sequence motifs at genomic glucocorticoid response elements (GREs) and nucleates combinatorial assembly of multicomponent transcriptional regulatory complexes, which modulate expression of cognate target genes (2). As for many vertebrate signal-activated TFs that coordinate broad gene expression programs, three features greatly complicate determination of transcriptional regulatory activity by a genomic GR-occupied region (GOR). First, in any given context, most individual GORs appear to lack direct regulatory function (3), and outnumber glucocorticoid-responsive genes by an order of magnitude or more (4–6). Second, a GRE may in fact comprise multiple GORs, together with multiple non-GR TF motifs, scattered over tens or hundreds of kilobases, each potentially contributing distinct regulatory outcomes. Third, GRE activities are highly context-dependent (2), and must be assessed in their normal chromosomal environments. As a result, very few GREs or other response elements have been fully functionally validated at native loci in living cells (7).

In principle, functional validation can be addressed by targeted genomic editing of GORs residing within candidate GREs, coupled with expression analysis of predicted cognate target gene(s). Edited subclones can be identified from bulk cell populations treated with a programmable nuclease (including zinc-finger nucleases, transcription activator-like effector nucleases (TALENs), and RNA-guided CRISPR-associated nucleases (e.g. Cas9)) using deep sequencing (e.g. sequencing by synthesis (SBS)) (8,9) to interrogate target regions. This requires multiplexing hundreds to thousands of samples and a computational workflow for deconvolution and analysis to identify genetic alterations that compromise GR binding sequence (GBS) motifs underlying GR occupancy, while also detecting concomitant changes to other vertebrate transcription factor binding site (TFBS) motifs. To accomplish these goals, three unmet needs were apparent:

1. *An index (barcode) set and Sample Sheet scaled sufficiently for SBS discrimination of thousands of subclonal genotypes*. In many Illumina^®^ sequencing applications, DNA sequences from distinct samples are labeled with barcodes, pooled (multiplexed) as a library applied to a single flow cell, and demultiplexed based on sample:index relationships specified in a Sample Sheet, which relates user-specified workflow parameters to Illumina^®^ sequencer control software. Illumina^®^ systems formally limit barcode assignments to <96 (single-index) or <384 (dual-indexed/paired-end) distinct samples. SBS analysis of independent samples at larger scale requires an augmented barcode set, and automated sample:barcode assignment in a Sample Sheet that specifies hundreds to thousands of data relationships.
2. *Rapid clonal genotype inference to identify potentially rare mutant clones with desired characteristics*. Excellent web-based and command line interface (CLI) tools (e.g. plateScreen96, which can be used to uniquely identify up to 96 individual samples (9), as well as CRISPR-GA (10), AGEseq (11), CRISPResso (12), CRISPR-DAV (13), Cas-analyzer (14), BATCH-GE (15), BE-Analyzer (16) and CRIS.py (17)) perform aggregate mutation counts and efficiencies from next-generation sequencing (NGS) data, and report population-distributed allele type resolution distinct from the bundled mutation frequencies returned by aggregate analyses that rely on Sanger sequencing (e.g. TIDE (18), TIDER (19)). We sought to develop a tool that specifically identifies alleles and resolves genotypes for single clones, and visually maps guide RNA sequence(s) on alignments to aid evaluation of mutations triggered by the action of programmable nucleases (e.g. Cas9).
3. *Automated TF binding site (TFBS) collation, to infer potential consequences of sequence variants or edits to TF function at candidate response elements*. Cell populations edited by programmable nucleases typically display a broad spectrum of alleles at targeted loci, generated as insertion/deletion (indel) outcomes of non-homologous DNA double-strand break repair processes (20). For indels in putative response elements, identifying mutation-associated loss or gain of putative TFBSs is essential for interpretation and/or prediction of functional consequences.

To serve these needs, we developed computational tools to automate sequence processing from input to output of a targeted editing effort focused on candidate response elements, seeking to expedite clone selection for retrieval and archiving, to prioritize clones for analysis based on inferred genotype definitions, and to anticipate potential functional consequences based on altered TFBSs. In a use case, we deployed these tools to analyze Cas9-induced mutations affecting candidate GREs associated with a glucocorticoid-regulated gene in a human adenocarcinoma cell line. Beyond the application we describe, these tools operate independently of one another and can be used to evaluate variation (i) at sequenced loci among cells clonally isolated from a heterogeneous population and (ii) directly on complex populations with variant alleles.

## MATERIALS AND METHODS

### Code overview and implementation

**Figure 1.**
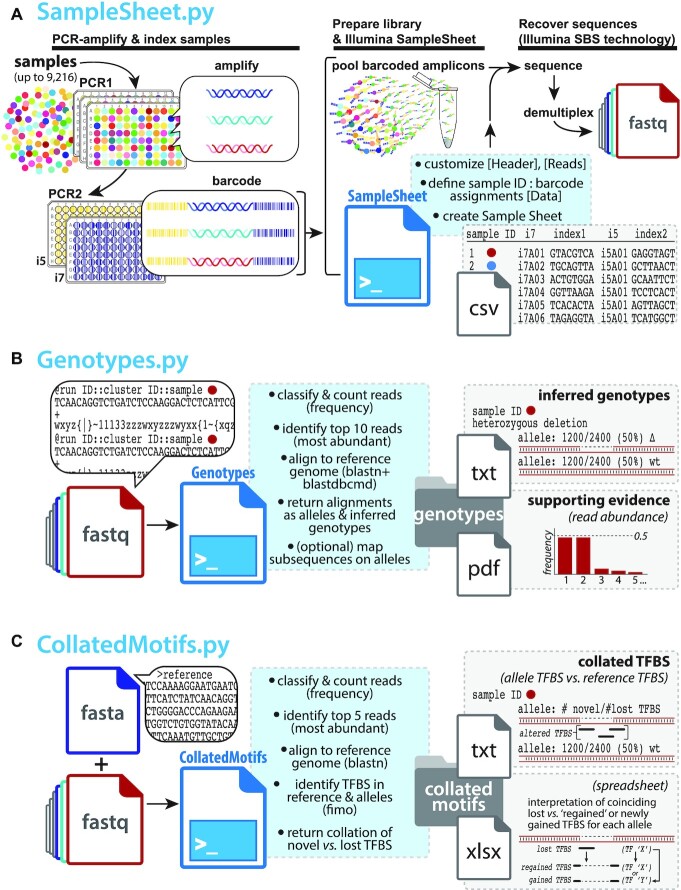
Three Python scripts facilitate analysis of genetic diversity in deeply sequenced amplicons. (**A**) SampleSheet.py automates construction of an Illumina^®^ Sample Sheet with up to 9216 sample:barcode relationships defined in its [Data] table. Briefly, target loci from up to 9216 samples can be amplified and indexed in two consecutive PCRs (PCR1 & PCR2), from essentially any nucleic acid source (e.g. Cas9-edited clonal isolates [*colored circles*]). After arraying genetic source material from individual samples in 96-well or 384-well (not shown) plates for amplification (PCR1), small amounts of each PCR1 product are used as templates in second reactions (PCR2) primed by pairs of uniquely barcoded forward and reverse primers compatible with Illumina^®^ sequencing platforms (ninety-six i7, ninety-six i5 barcode possibilities). Barcoded amplicons are pooled as a library; user-supplied values at SampleSheet.py prompts are expanded to populate a Sample Sheet with sample:barcode designations, enabling read demultiplexing into up to 9216 sample-specific fastq files following Illumina^®^ SBS; (**B**) Genotypes.py accepts any number of fastq files as input, applying Python counter functions to classify and count read frequencies. After aligning the most abundant reads to a reference genome (BLASTN), alleles are hypothesized and defined based on relative read frequencies and alignment comparison to the wild-type reference (e.g. SNPs, indels); genotypes are inferred based on allele definitions. Optionally, DNA subsequences (short oligonucleotide sequences) can be mapped onto allele outputs to flag positions and/or presence/absence of specific sequence motifs; (**C**) CollatedMotifs.py accepts fastq files as input, along with a single fasta file defining reference sequence(s). Like Genotypes.py, CollatedMotifs.py identifies candidate alleles by read frequency and alignment to a reference sequence; it then identifies and compares matches to TFBS motifs in reference and allele sequences (Meme FIMO), returning a visualization and spreadsheet of novel and lost TFBS in each allele.

**Figure 2.**
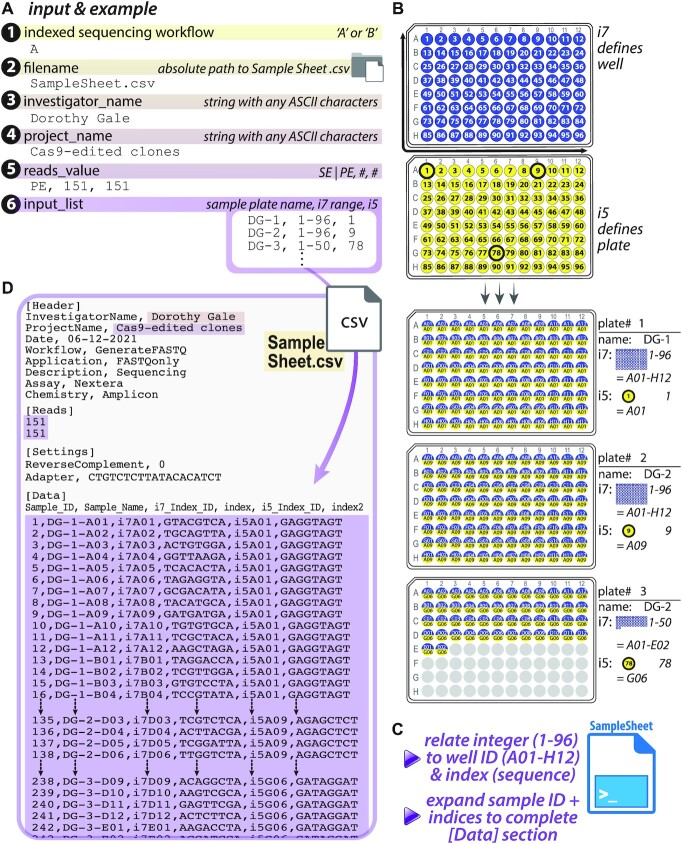
SampleSheet.py: automated Illumina^®^ Sample Sheet construction for sequencing and demultiplexing of up to 9216 barcoded samples. (**A**) SampleSheet.py anticipates user-defined, console-supplied entries for six variables, which define (1) the Illumina^®^ Indexed Sequencing Workflow (‘A’ or ‘B’); (2) the absolute path at which the Sample Sheet file will be created; and subsections of an Illumina^®^ Sample Sheet: two [Header] values (3) ‘InvestigatorName’ & (4) ‘ProjectName’, the [Reads] section (indicating (5) the number of sequencing cycles for single-end or paired-end formats) and (6) a list of sample plate names and i7 + i5 barcode permutations, the principal [Data] output; (**B**) PCR strategy that links amplicons to barcodes, which underlies the relationship that SampleSheet.py creates between an individual sample identity (plate and well ID) and its distinctive i7 + i5 barcode combination. In this strategy, i7 sequence (blue) defines individual wells of a 96-well plate, and is used across plates; i5 (yellow), in contrast, defines up to all wells of a single plate; (**C**) SampleSheet.py performs automated expansion of appropriate sample:index relationships in the Sample Sheet [Data] table, (**D**) output in the Sample Sheet for up to 9216 samples. In the example given, samples arrayed in three 96-well plates have been uniquely labeled, and an input list with three lines of text is expanded into a list of 242 entries, with index sequences accurately presented in the Sample Sheet.

**Figure 3.**
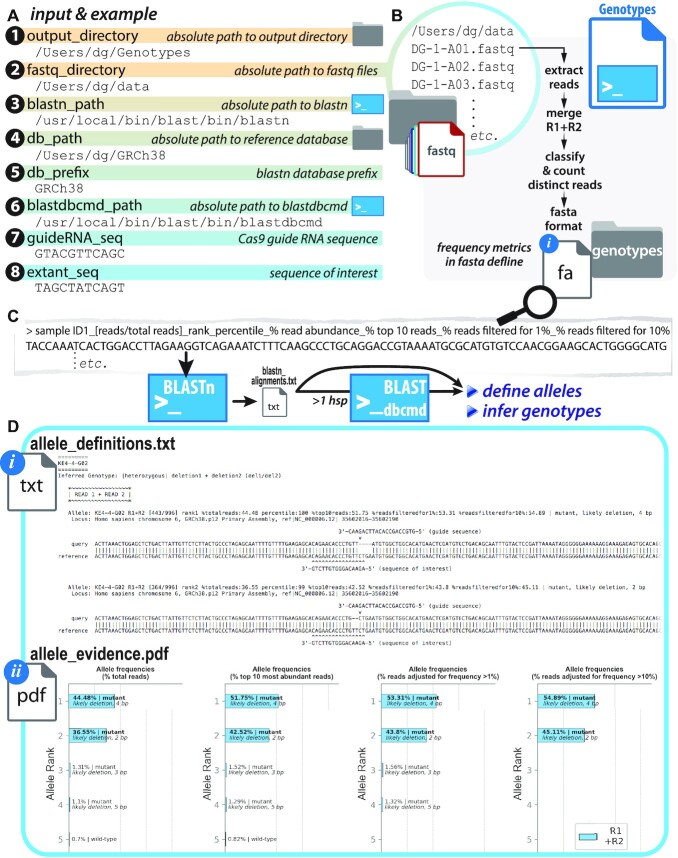
Genotypes.py: allele definition and genotype inference based on read abundance in deeply sequences amplicons. (**A**) Eight user-defined inputs specify locations of three directories and two executable files (BLASTN and BLASTDBCMD), as well as two optional short nucleotide sequences to be mapped onto analyzed sequence outputs—ultimately generating eight output files; (**B**) fastq files are read into Genotypes.py, which classifies merged paired-end reads by relative representation (Python counter function) and defines alleles based on calculated frequency. The top ten most abundant reads (‘ranked alleles’) are labeled with sample ID and abundance/representation and populated into a fasta file (*fasta.fa*), which is (**C**) passed to BLASTN for alignment to a position in the reference genome/sequence provided as a BLASTN database in (A). The output alignment file (*blastn_alignments.txt*) is parsed from html format to populate lists and dictionaries with read-specific metadata (allele identifier, ‘hit’ position in reference sequence, alignment to ‘hit’); these data are the basis of allele type *definition* (deletion, insertion, wild-type, etc.) and subsequent genotype *inference*; reads with alignments that span multiple BLASTN high-scoring pairs (*hsp*’s) are further processed by BLASTDBCMD to reconstitute alleles with potentially long (>∼60 bp) indels; (**D**) eight output files are generated (*allele_definitions.txt, allele_definitions.csv, allele_evidence.pdf* (optional)*, blastn_alignments.txt, fasta.fa, genotypes.txt, population_summary.txt, script_metrics.txt*); portions of the principal output in *allele_definitions.txt* and *allele_evidence.pdf* are shown (see Supplement for further examples).

**Figure 4.**
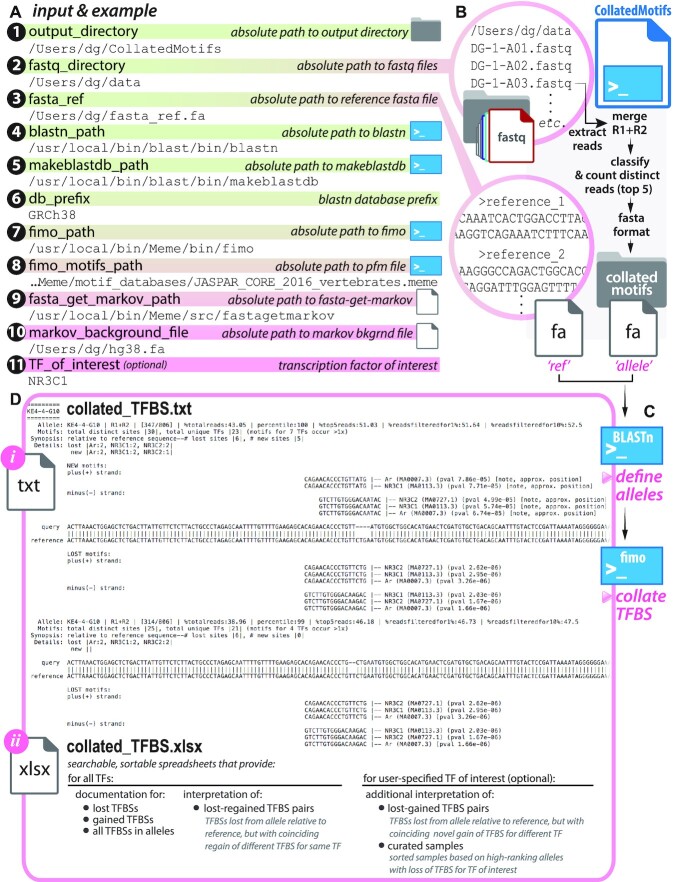
CollatedMotifs.py: identification of altered regulatory motifs in defined alleles, relative to reference sequence. (**A**) Eleven user-defined inputs specify locations of two directories, four executable files (BLASTN, FIMO, MAKEBLASTDB, FASTA-GET-MARKOV), three files (fasta file with reference sequences, text file with TFBS motifs, text file with sequence(s) from which markov background will be defined), a single database prefix string, and a TF of interest (optional); (**B**) fastq files are read into CollatedMotfis.py, which identifies the top 5 most abundant merged paired-end read types for each sample; (**C**) reference sequences in user-provided fasta file and fasta file containing top 5 reads for each sample are provided to BLASTN for alignments and to FIMO for TFBS determinations, (**D**) with alignments and collated TFBS (lost and/or gained) displayed in output files *collated_TFBS.txt* and *collated_TFBS.xlsx*. Due to the gap in the aligned query sequence, when a deletion mutant gains a TFBS, the motif alignment to the query matches only one side within the output and ‘*note, approx. position*’ is displayed next to motif pval.

We developed code to support and expedite a workflow that reports locus-specific alleles, genotypes and associated TFBS differences for up to 9216 clones from a single Illumina^®^ SBS run (Figure 1). *SampleSheet.py* (Figure 2) automates preparation of an Illumina^®^ Sample Sheet, the text document that defines well:barcode assignments for demultiplexing on Illumina^®^ sequencing platforms. *Genotypes.py* (Figure 3) facilitates rapid convergence to genotype from demultiplexed fastq files (21), simplifying identification of cultured clones of interest to archive and examine. *CollatedMotifs.py* (Figure 4) summarizes alterations to TFBS motif matches for each sample (clonal isolate) relative to a reference sequence, capitalizing on public repositories of sequence-selective position frequency matrices for characterized DNA-binding regulatory factors. Natural variation or DNA sequence alterations associated with editing by programmable nucleases (e.g. Cas9) in putative response elements may cause losses or gains of TFBSs, making their annotation in clones important for interpretation of potential functional consequences. Scripts are available as annotated Jupyter notebooks (22) (.ipynb) for interactive use in a web browser and as program files (.py) that can be run at a command-line interface (CLI) at https://github.com/YamamotoLabUCSF, and pre-compiled in Open Virtualization Format for virtualization (e.g. in Oracle VM VirtualBox, https://www.virtualbox.org/) with supporting resources, example input and output files, and test datasets at https://doi.org/10.5281/zenodo.3406861. All scripts require Python 3.7 or greater for operation on Mac OSX, Linux or Windows systems. A full list of dependencies can be found in Supplementary Table S1 and in the README.md file associated with each program at the GitHub repository (https://github.com/YamamotoLabUCSF). The key inputs and outputs of each computational tool are described below. Further descriptions of the tools as well as user guidelines, script operation summaries, and explanations of the contexts and uses of additional output files (outside of the particular experimental examples detailed below) are available in the Supplementary Guidelines.

**Figure 5.**
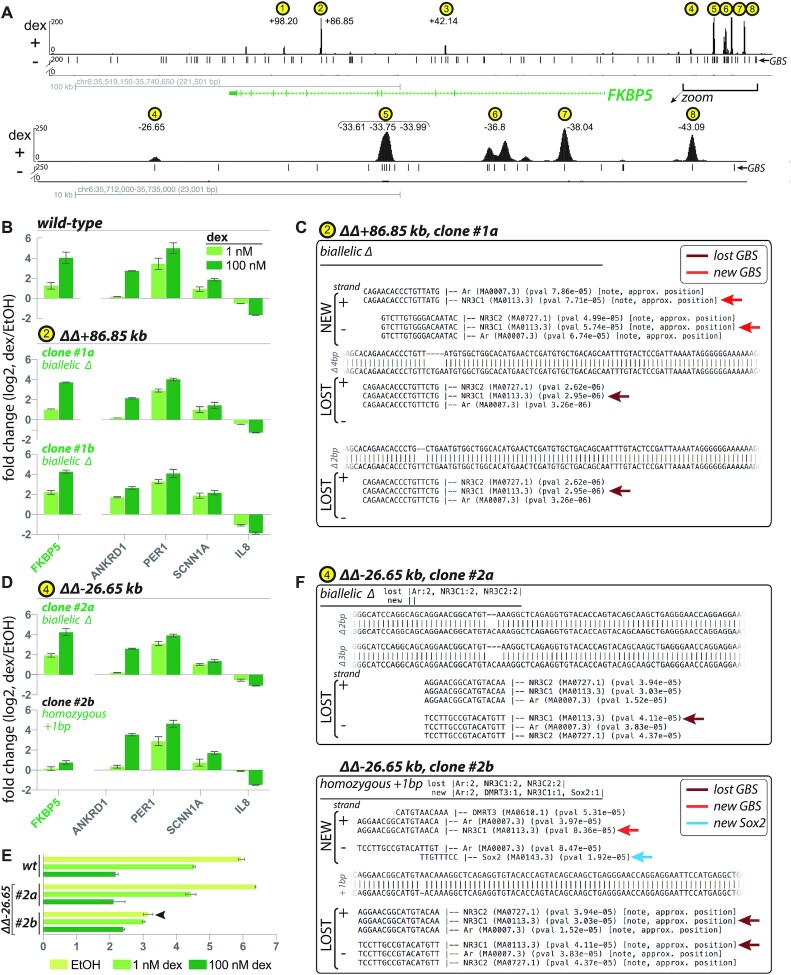
Use case: evaluation of Cas9-altered loci occupied by human glucocorticoid receptor (GR) near a glucocorticoid-regulated gene, *FKBP5*. (**A**) *top panel*, GR ChIP-seq (A549 ± 100 nM dex, 1.5 h) indicating eight intronic and promoter-proximal dex-dependent GR-occupied regions (GORs (yellow circles)) in vicinity of dex-induced *FKBP5*; GOR coordinates defined as peak summit distance from FANTOM5-defined TSS for *FKBP5* transcript variant 1 (RefSeq NM_004117, coordinates chr6:35,688,937 in GRCh38) (36); *lower panel*, zoom-in of region comprising GOR4-8; (**B**) regulatory analysis (fold change (log_2_) of mRNA levels) for five dex-responsive genes (*FKBP5* and four control genes on separate chromosomes, *ANKRD1*, *PER1*, *SCNN1A*, *IL8*) sampled from A549 (wild-type and GOR mutants) ±1 nM and 100 nM dex, 4 h (RT-qPCR, ΔΔCT dex relative to ethanol control, n = 3, mean ± sd), for wild-type compared to two clones with biallelic disruption to the GBS at GOR2 (+86.85 kb) peak summit: Clone #1a (biallelic Δ in GBS) and Clone #1b (biallelic Δ across entire GBS); (**C**) CollatedMotifs.py TFBS annotations in ΔΔ+*86.85 kb* clone #1a show that both alleles lose native GBS (maroon arrow, *lost GBS*), but one allele reconstitutes a novel GBS (red arrow, *new GBS*); (**D**) Clones #2a and #2b (biallelic Δ and homozygous + 1 bp in GBS at GOR4 (-26.65 kb) peak summit) exhibit distinct regulatory consequences for *FKBP5* induction, unique among evaluated genes; (**E**) ΔCT analysis (CT *FKBP5* – CT_geometric mean_ for three reference controls (*GAPDH, HBMS, RPL19*)) indicating basal (EtOH control) and induced (+dex) levels (*n* = 3, mean ± SD) indicates that loss of *FKBP5* dex induction in ΔΔ-*26.65 kb* clone #2b is partly attributable to increased baseline (EtOH) transcript level (arrow) relative to wild-type (*n* = 3, mean ± SD); (**F**) CollatedMotifs.py TFBS annotations in ΔΔ–26.65 kb clones #2a versus #2b show that both clones lose native GBS (maroon arrow, lost GBS), but clone #2b reconstitutes a novel GBS (red arrow, new GBS). Furthermore, clone #2b also exhibits TFBS for novel TFs (e.g. blue arrow, *new Sox2*), suggesting an avenue to further examine—and potentially explain—ostensible mutation-specific regulatory phenotypes for response elements under study.

### Experimental use case

GBS motifs underlying eight GORs (GR ChIP-seq summits) in a putative topological unit encompassing the dex-responsive gene *FKBP5* were targeted for Cas9-mediated mutagenesis in A549 (Figure 5); clonal isolates were recovered by flow-assisted cell sorting into 96-well plates. Target loci were PCR-amplified from lysates in individual wells (PCR1), appended with Illumina flow cell adaptors and custom barcodes to label sample-specific identities (PCR2), and pooled for deep sequencing (paired-end 2 × 150) on an Illumina MiSeq (Figures 1 and 2). SampleSheet.py was used to create a Sample Sheet to demultiplex reads to sample sources (Figure 2). Resulting fastq files were directly supplied as inputs to Genotypes.py (Figure 3) and CollatedMotifs,py (Figure 4) for analysis. See Supplementary Methods for experimental details.

## RESULTS

### Automated assignment of paired-end barcodes to samples: SampleSheet.py

Illumina^®^ SBS workflows entail library construction, cluster generation, sequencing, and data processing, where a single, plain-text, comma-separated (*.csv) file—the Sample Sheet—mediates communication of user preferences and sequencing workflow specifications to the software overseeing sequencing operations and data acquisition. The Sample Sheet documents fundamental run properties (e.g. sequencing cycle number, chemistry) and key links between library identities and barcodes, allowing reads to be demultiplexed into individual fastq files after sequencing. Illumina^®^ provides an excellent whitepaper describing Sample Sheet sections and preparation in Pub. No. 970–2017–004-A (2017).

For small numbers of indices (e.g. tens), samples and their identifying barcodes are easily compiled manually in a text document. For hundreds to thousands of samples, however, manual compilation is time-consuming and prone to error (e.g. typos or mistaken entries that lead to sample:barcode mispairings). SampleSheet.py automates Sample Sheet construction, allowing a Sample Sheet with up to 9216 data entries to be compiled in seconds from a single list of up to 96 short lines of text (Figures 1A and 2), linking sample IDs in 96-well plates to specified i7 (Illumina index 1) and i5 (Illumina index 2) barcode pairs (23). SampleSheet.py draws upon a custom library of 192 8-nucleotide barcode primers compatible with Illumina^®^ sequencing platforms (Supplementary Figure S1)—96 ‘forward’ and 96 ‘reverse’ as defined by sense/antisense to read 1 orientation in the Illumina^®^ MiSeq workflow. Easily ordered and accessed in 96-well plate format (Supplementary Excel file), permutations of the ninety-six i7 (read 1) and ninety-six i5 (read 2) barcodes allow unique labeling of up to 9216 distinct samples. Detailed user guidelines are provided in Supplementary Guidelines (SampleSheet.py).

### SampleSheet.py operations and Sample Sheet output file

SampleSheet.py creates a comma-separated file interpretable by Illumina^®^ software as a Sample Sheet. To establish sample:barcode pairings based on user input, SampleSheet.py prompts users for six values, entered as text at individual Jupyter interface or CLI prompts (Figure 2A, Supplementary Table S2, Supplementary Figures S2, S3A). These values include: (i) Illumina^®^ Indexed Sequencing Workflow (A versus B, Supplementary Figure S4), (ii) absolute path to output directory and filename for Sample Sheet, (iii) Investigator Name, (iv) Project Name, (v) Single-end (SE) or Paired-end (PE) sequencing run with cycle number(s), and (vi) list of sample:barcode relationships.

The key utility of SampleSheet.py is to automate large numbers of sample:barcode relationships in the [Data] table section of a Sample Sheet. In principle, the custom i7 and i5 primers (Supplementary Figure S1) can be used in any combination with one another, but in the workflow described, the i7 barcodes act as well IDs (A01-H12 of a 96-well plate), while the i5 barcodes act as plate IDs (plate 1 through 96); the combinations of 96 i7 by 96 i5 barcodes allow for up to 9216 clones/loci, across ninety-six 96-well plates, to be genotyped in a single experiment (Figures 1A and 2C).

These specified uses of i7 and i5 barcodes are achieved by attention to primer selections when preparing amplicon libraries by two successive PCR reactions (Figure 1A, Supplementary Figures S1 and S5). Samples to be PCR-amplified for amplicon sequencing are arrayed in 96-well plates with each sample containing a unique well ID + plate ID combination. In a first PCR (PCR1), the target locus is amplified from a genetic sample source (such as isolated DNA, cDNA, or crude lysate) using primers each containing a 5' common adaptor and a 3' target-specific sequence. In a second PCR (PCR2), universal well- and plate-position-specific primers containing the i7 adaptor:index1 barcode:common adaptor1 and the i5 adaptor:index2 barcode:common adaptor2 (see Supplementary Excel File) are used to amplify the original PCR1 product (Figures 1A and 2B–C, Supplementary Figures S5, S6). Automation for PCR1/PCR2 is described in Supplementary Figure S7.

Provided with user-specified designations for individual 96-well plate names, the script ultimately outputs unique corresponding sample names for all specified wells, with each unique full sample name articulated as ‘Plate name-Sample well position’ in the Sample Sheet. Plate name(s) and i7, i5 oligo IDs are user-entered at the final console prompt, requiring only a single line of three comma-separated values per 96-well plate (plate name, i7 barcode ID range, i5 barcode ID) to populate up to 96 uniquely named and barcode-identified well entries in a Sample Sheet (Figure 2A, input #6; Supplementary Figure S3A, Supplementary Figures S4–S6). For 9216 entries in Sample Sheet format, only 96 lines of text are required, dramatically reducing labor and error-potential relative to manual entry of 9216 assignments—the key utility of SampleSheet.py. The i7/i5 barcodes and script provided here are equally useful for samples arrayed in a single 96-well plate as for samples arrayed across the maximum capacity at ninety-six 96-well plates (i.e. useful for 1–96 plates). Upon sample:barcode range entry at the command-line, generation and completion of the corresponding Sample Sheet text file is virtually instantaneous (<1 s).

### Allele definition and genotype inference at specific loci of individual clones: Genotypes.py

Whether focused on non-coding or coding genomic sub-regions, allele definition and genotype deduction at experimentally examined loci are common goals of population genetic analyses, including identification of mutant clones downstream of targeted editing. We developed Genotypes.py, a script that converts reads in individual sample-specific fastq files into deduced genotypes for those samples at PCR-amplified (queried) loci (Figures 1B and 3). The script defines alleles based on relative read abundance (frequencies), and infers corresponding sample-specific genotypes. Most similar computational tools describe population-level allele frequencies from deeply sequenced loci (10–17). In addition to population-level allele frequencies (aggregated across multiple samples), Genotypes.py reports predicted genotypes for individual samples (e.g. clones), parsing BLASTN (24) read alignments to reference sequence(s) to define alleles.

BLASTN excels at alignments for sequence spans that differ from one another by insertion/deletion stretches that range between 1–60 bp—a predominant indel range generated during targeted genome editing—making it an excellent choice for alignment-based allele definition at loci targeted by programmable nucleases and PCR-amplified for short-read sequencing. Longer deletion spans (>60 bp) may also arise as products of targeted editing (such as by delivery of two targeted nucleases to distinct sites). Because alignments with gap (indel) spans that exceed ∼60 bp are split into multiple high-scoring alignment pairs by BLASTN, Genotypes.py further evaluates alignment data for reads with high-scoring pairs (hsp's) if the distance spanned by the hsp's is within 1 kb, reasoning that these BLASTN outputs may be representative of longer indel alleles. Genotypes.py invokes BLASTDBCMD to retrieve the reference sequence spanned by the hsp's, and then reconstitutes a predicted indel allele to include in genotype inferences. This feature enables alleles that differ from a reference by even several 100 bp (e.g. long deletions) to be reconstituted from alignments on paired-end reads. We observed that Illumina sequencing chemistry is quite robust to amplicon length variations ranging from 100–800 bp (Supplementary Figure S8). Detailed user guidelines are provided in Supplementary Guidelines (Genotypes.py).

### Genotypes.py operations and output files

Foremost, Genotypes.py converts raw sequencing read data to proposed allele and genotype representations for demultiplexed samples. Its key operations center on discernment of distinct reads, assessment of relative read frequencies, definition of proposed alleles as wild-type or mutant relative to an alignment reference, and genotype hypotheses based on ranked allele abundances. Its key outputs provide, for user evaluation, visually accessible evidence for allele definitions and hypothesized genotypes (Supplementary Figure S9). Genotypes.py prompts users for up to eight values—six required and two optional—entered as text at Jupyter Notebook or CLI prompts (Figure 3, Supplementary Table S3, Supplementary Figure S3B). These include: absolute paths to (i) input and (ii) output directories, (iii) BLASTN executable and (iv) alignment reference database; (v) reference database file prefix; (vi) BLASTDBCMD executable; and (optional) (vii, viii) DNA sub-sequences to display on alignments. The script delivers up to eight output files in a user-defined directory location. Content and uses of four key output files (*allele_definitions.txt*, *genotypes.txt*, *allele_evidence.pdf*, *population_summary.txt*) are described here; additional files generated as intermediates during script processing and to log script operations are described in Supplementary Guidelines, Supplementary Table S4, and Supplementary Figure S9.

### Ranked allele definitions and inferred genotypes

Genotypes.py displays its findings in two text files, *allele_definitions.txt* and *genotypes.txt*; for each sample ID, *allele_definitions.txt* reports genotype, followed by ‘alleles’ (up to ten sequences ranked by relative frequency) identified from merged read 1 (R1) and read 2 (R2) sequences (Figure 3D, Supplementary Figure S9). Three text blocks report frequency metrics, allele specifications and alignments: (i) ‘*Allele’* reports sequence name (fasta defline containing sample ID and frequency metrics) and allele specifications (definition as ‘wild-type’ or ‘mutant’ relative to reference sequence (BLASTN ‘hit’), and if ‘mutant’, further resolution as ‘likely deletion | insertion | substitution | complex indel’, including number of bp altered by the mutation (e.g. ‘likely deletion 8 bp’); (ii) ‘*Locus’* reports details of alignment database ‘hit’, defined by BLASTN database content (typically, locus identifier and coordinates); (iii) ‘*Alignment’* reports allele sequence relative to reference ‘hit’ sequence, with midline (pipe (‘|’) versus gap) reporting matched *vs*. unmatched nucleotide positions. If DNA sub-sequences were provided for annotation, Genotypes.py maps the position(s) of these sequence(s) above (if guide RNA or target binding sequence for a programmable nuclease) or below (if query sequence provided to be tested for presence *vs*. absence in allele) each alignment in which the sequence(s) were identified in an allele or its reference, facilitating interpretation of indels as plausible consequences of programmable nuclease-directed mutagenesis, and/or assessment of query (test) sequences for presence *vs*. ablation/absence. Ranked sequences that occurred at adjusted frequency <10% are demarcated from other alignments with text highlighting their scarceness as likely artefacts for studies probing homogenous clonal isolates (not representing genetic source sequences): ‘*>>>>> remaining alleles occur at frequency <10% <<<<<*’.

Like *allele_definitions.txt*, the file *genotypes.txt* reports allele definitions and alignments for ranked alleles, but reports samples based on inferred genotype class. In other words, a ‘homozygous deletion’ cohort is reported before a ‘homozygous insertion’ cohort, in turn reported before ‘heterozygous’ cohorts, followed by a ‘homozygous wild-type’ cohort and finally, a cohort for which insufficient evidence was recovered to infer genotype (‘unclear or multi-allelic, insufficient representation of allele(s)’) (Supplementary Table S5). This format provides an organized list of identified mutant clones, useful for further experimental processing and long-term storage. In addition to their summation in *allele_definitions.txt* and *genotypes.txt*, allele data compiled in *genotypes_dict* are transferred to a pandas dataframe for output in the spreadsheet-compatible *allele_definitions.csv* file, providing convenient user access to raw data for the sample-specific, frequency-ranked alleles used in genotype deductions (Supplementary Figure S9).

### Display of ranked allele frequencies (optional)

As visual evidence for genotype inferences, Genotypes.py reports merged R1 + R2 ranked sequences in frequency plots printed to *allele_evidence.pdf* (Figure 3D, Supplementary Figure S9), an optional output file that highlights the ranked sequence subset representing alleles most likely to account for sample genotype. For each sample, ranked sequence abundance is rendered as (i) raw frequency (% total reads), (ii) % top 10 reads, (iii) % reads filtered for reads occurring at >1% raw frequency, (iv) % reads filtered for reads occurring at >10% raw frequency. A sizeable fraction of reads that occur at <1% frequency in a fastq file are attributable to template differences introduced by sequencing or PCR artefacts (25), justifying their exclusion in plots (iii) and (iv) and the frequency recalibration of more abundant sequences. Cumulative generation of these plots can be time-intensive (e.g. ∼2 seconds per pdf page depending on system resources), and this code passage is therefore optional in Genotypes.py; after initial user input and just before script operations begin, a user is prompted to specify whether to include (‘Y’) or bypass (‘N’) frequency plot generation and assembly into a pdf file. Together, allele_evidence.pdf, genotypes.txt, and allele_definitions.txt provide complementary metrics that support the genotype inferences delivered by Genotypes.py; moreover, the availability of ten frequency-ranked alleles can help users detect potential copy number variation.

### Population-wide genotype distribution summary

Although Genotypes.py chiefly hypothesizes genotypes for individual samples demultiplexed from a potentially diverse library population, the program also reports aggregate population properties in *population_summary.txt* (Supplementary Figure S9). In ‘Synopsis of Interpretations: Allele Definitions & Genotype Inferences’, the script catalogs (i) the fraction of samples for which a genotype was inferred, (ii) overall genotype properties represented in the sample population (e.g. % samples diploid (1–2 prominent alleles inferred) versus % multiploid (>2 prominent alleles inferred), % homozygous wild-type versus homozygous mutant (subsetted for deletion, insertion, substitution, complex indel), % heterozygous (wt + mutant), subsetted as above, % heterozygous (mutant + mutant), etc.) and (iii) overall alleles represented (e.g. % wild-type alleles, % mutant alleles (deletion, insertion, substitution, complex indel)). In ‘Synopsis of Reads Lost to Analysis’, the script earmarks frequency-ranked reads that were deprecated from genotype inference, due to (i) no hits, or (ii) multiple hits, in the reference database (for sequences with ‘no hits’, a user may wish to use BLAST online to identify non-target sequence(s) detected as amplified from sample source; for sequences with multiple hits, a user may choose to recast (constrain) the reference database to focus target alignment, and/or may choose to redesign primers or PCR conditions to improve specificity in future amplicon libraries for the locus in question). Ranked reads identified for potential allele reconstruction with BLASTDBCMD are also subject to deprecation if their high-scoring pairs (hsp's) overlap or span >1 kb.

Taken together, Genotypes.py outputs provide users with well-documented, evidence-based genotype inferences for deeply sequenced amplicons based on frequency representations among demultiplexed reads in fastq files and comparison to user-provided reference sequence(s), automating genotype definition from one to thousands of loci amplified from individual genomic sample sources.

### Identification of altered TFBS motifs in individual mutant clones: CollatedMotifs.py

Editing efforts targeted to putative genomic response elements demand careful annotation of resulting alleles—specifically, to monitor loss, gain, or ‘reconstitution’ of TFBSs consequent to genetic alteration. Widely available pattern-matching tools enable prediction of TFBSs in a query sequence based on matches to position frequency matrices of known TFs. We developed CollatedMotifs.py to automate identification and comparison of TFBS motifs between sample-specific alleles and a user-supplied reference sequence. Detailed user guidelines are provided in Supplementary Guidelines (CollatedMotifs.py).

### CollatedMotifs.py operations and output files

Like Genotypes.py, CollatedMotifs.py reports hypothesized alleles for demultiplexed NGS datasets, but its sequence alignments are populated with matches to TFBS motifs—specifically, with TFBSs lost or gained in an allele sequence relative to a user-provided reference sequence. Its key operations prepare data resources used by BLASTN (24) and FIMO (26) (such as a BLASTN alignment database and background Markov file from user-defined sequence(s)), and supply visually accessible evidence for allele definitions and associated TFBS comparisons (Supplementary Figure S10). CollatedMotifs.py prompts users for up to eleven inputs entered as text into the Jupyter Notebook or CLI prompts. Ten required inputs include nine absolute paths to directories, files, or executables, and one prefix for alignment database files. The name of one transcription factor (TF) of interest can be included as an optional eleventh input (Figure 4, Supplementary Table S5). The script delivers six output files and three sub-directories in a user-defined location. Content and uses of three key outputs (*fimo_out, fimo_out_ref*, *collated_TFBS.txt, collated_TFBS.xlsx*) are described here; additional files and sub-directories generated as intermediates during script processing and to log script operations are described in Supplementary Guidelines, Supplementary Table S6, and Supplementary Figure S10.

### Comparison of TFBSs in hypothesized alleles relative to the reference sequence: fimo_out and fimo_out_ref

The purpose of CollatedMotifs.py is to provide users with an overview of changes in sequence-based TFBS predictions that occur in sample alleles relative to a user-provided reference (e.g. ‘wild-type’) sequence. After initial allele definition based on read frequency counts and BLASTN alignments to a reference sequence database, the script advances to identification of matches to TFBS motifs in DNA sequences, invoking FIMO. FIMO separately queries two fasta files*—*(i) the reference sequence file provided at the script outset by a user, and (ii) a fasta file (*fasta.fa*) containing sample-specific inferred candidate allele sequences generated by CollatedMotifs.py; FIMO evaluates sequences in these files for TFBS matches to motifs in a user-supplied position frequency matrix (positional weight matrix/PWM). For each of the two fasta files, CollatedMotifs.py directs five FIMO default output files (*cisml.xml, fimo.gff, fimo.html, fimo.tsv, fimo.xml*) to one of two script-generated subdirectories, *fimo_out_ref* or *fimo_out* (Figure 3C). By default, FIMO reports TFBS matches at a p-value threshold of 0.0001 (1e–4), but users can adjust this threshold by adding the flag *–thresh* with a revised value to the script's FIMO operation call (details for this and other flags can be found at http://meme-suite.org/doc/fimo.html), or by using the variant script CollatedMotifs-with_user-set_pval available in the GitHub repository.

### Collated TFBS losses and gains in ranked alleles

The distinctive output of CollatedMotifs.py is the collation of TFBSs for reference sequence(s) and putative alleles. For each sample-associated, ranked allele, CollatedMotifs.py determines the appropriate user-supplied reference sequence with which to pose a comparison (Figure 4). For each sample ID*, collated_TFBS.txt* reports a visual mapping of (i) TFBSs new to each allele above the alignment and (ii) TFBSs lost from each allele below the alignment (e.g. ‘new TFBS’ and ‘lost TFBS’) (Figure 4D, Supplementary Figure S10). All inferred allele alignment data and FIMO-identified TFBSs are archived in *collated_TFBS.xlsx*, an Excel file containing up to eight tabbed worksheets that further organize interpretations for TFBSs lost and/or gained across ranked alleles for every sample. If users input the optional TF of interest, CollatedMotifs.py will add worksheets that specifically display alleles that have lost a TFBS for the specified TF, with or without regain of an alternative TFBS for the same TF or gain of a TFBS for a distinct TF (see Supplementary Figure S10 for details).

The outputs of CollatedMotifs.py are well-documented, evidence-based genotype inferences for deeply sequenced amplicons established on frequency representations among demultiplexed reads in fastq files. By applying comparison of TFBS identification relative to user-provided reference sequence(s), CollatedMotifs.py annotates each allele alignment with TFBSs (and associated FIMO-defined p-value matches to PWMs) collated as ‘lost’ or ‘new’.

### Use case: Cas9-edited disruptions of glucocorticoid receptor-bound loci near a glucocorticoid-regulated gene, *FKBP5*

As a use context and developmental proof of principle, we used the 96 × 96 barcoded primers and the three computational tools described here to identify and characterize mutants among thousands of Cas9-treated clones in the human adenocarcinoma cell line A549, specifically seeking disruptions of glucocorticoid receptor-occupied regions (GORs) near the glucocorticoid-responsive gene *FKBP5* (Gencode v32 gene ENSG00000096060.14). *FKBP5* is strongly glucocorticoid-induced in many human cell types examined; in A549 cells, the *FKBP5* gene body is characterized by promoter-proximal and intronic (27) GORs (Figure 5A).

Glucocorticoid resistance in humans—associated with recurring lifetime vulnerability to major depressive disorder (MDD) and other brain diseases—is potentially associated with higher induced levels of *FKBP5* (28,29). Several GORs proximal to *FKBP5* house GR binding site (GBS) motifs with high evolutionary conservation across 100 vertebrates examined, suggesting early emergence of these sequences in the vertebrate lineage and negative selection against changes to these GBSs occurring across vertebrate lineages and timespans approaching or exceeding 400 million years (Supplementary Figure S11).

We examined consequences of individual disruptions of eight GORs in a 1.5 Mb genomic region (GRCh38/chr6:34,950,000–36,450,000), in which *FKBP5* occurs as the only dexamethasone-responsive gene within a putative topological domain comprising ∼400 kb (chr6:35,339,500–35,740,000, and encompassing the genes *PPARD, FANCE, RPL10A, TEAD3, TULP1, ARMC12*). *FKBP5* mRNA is induced ∼15-fold within 4 h of dexamethasone (dex, a pharmaceutical glucocorticoid) exposure (100 nM) (Figure 5C), the only gene body affected >1.5-fold in this region (L. Pack & K. Yamamoto, unpublished; (30)). In brief, Cas9 sgRNA sequences were cloned downstream of a U6 promoter in a puromycin-selectable vector expressing *Streptococcus pyogenes* Cas9, producing functional Cas9 RNPs *in vivo* when transfected into cells (RRID:Addgene_62988, procedure detailed in Supplementary Methods); after puromycin selection, single cells from Cas9-treated cell populations were FACS-isolated into individual wells of ninety-six 96-well plates for arrayed clonal expansion and amplicon barcoding (Supplementary Methods, Supplementary Figures S5 and S7). Amplicons from 96-well plates were pooled and sequenced on an Illumina MiSeq with excellent (>99%) barcode pair detection across 9216 samples (Supplementary Figures S12 and S13). A single list of 96 plate:barcode relationships was prepared in plain text format to present as [Data] input to SampleSheet.py; SampleSheet.py rendered the Sample Sheet used for sequencing and demultiplexing in 0.05 s (<2 min total user interaction with script).

After sequencing and demultiplexing reads into 9216 × 2 (18,432 R1 + R2) fastq files containing sample read content, fastq files were submitted to Genotypes.py in batches corresponding to locus and sgRNA (i.e. individual editing scenarios, 384–480 samples/768–960 fastq files per batch). On a typical 2017 MacBook Pro laptop with 16 GB RAM and a 4-core CPU, allele definitions and inferred genotypes were completed for each batch and returned in *allele_definitions.txt* and *genotypes.txt* within 1.2 min (mean), with population statistics completed in *population_summary.txt* in <20 s (total genotype deduction and text file reports completed within 2 min); altogether, genotypes for 9216 samples were complete within 35 min. Visual evidence in the form of frequency plots (*allele_evidence.pdf*) was completed within 6 h.

Despite low overall editing frequencies (1–10% altered alleles across individual editing experiments, Supplementary Figure S14) and generally small indels at edited loci (e.g. generally < 5 bp, which would be undetectable by typical gel-based fragment size analysis), Genotypes.py readily identified clonal cell lines with biallelic disruptions to the targeted GBS motif(s) for five of the eight GORs. We selected clones based on mutant genotypes at target GORs, and evaluated consequences to *FKBP5* regulation by RT-qPCR (Supplementary Methods). Interestingly, most tested ablations did not significantly impact dex-induced *FKBP5* mRNA accumulation (4 h) as measured by RT-qPCR. For example, consider the GBS that underlies the GR ChIP-seq peak summit at GOR *+*86.85 kb, a GBS with high vertebrate conservation and able to drive dex-induced luciferase reporter expression when cloned upstream of a minimal promoter and transfected into A549 cells. Clones with either small (<5 bp) or larger (>60 bp) biallelic disruptions within (small indels) or spanning (large deletions) the GBS displayed little effect on dex-responsive *FKBP5* mRNA induction (Figure 5B and C; Supplementary Figure S14).

The strong evolutionary conservation of the GBS sequence implies that GOR *+*86.85 kb is functional in certain biological settings, although regulatory activity displayed on a transiently transfected reporter (Supplementary Figure S14, (4)) does not accurately predict activity in the normal chromosomal context. The more likely explanations are either assay context or physiological context. The former recognizes that we are measuring activity at a single time point after treatment with a single dex concentration, whereas activity might be evident in kinetic, time-course or dose-response assays. Physiological context acknowledges that we are seeking GOR activity in cultured A549 cells, whereas function may reside in other cell types, in response to alternative glucocorticoid ligands, in combination with other signaling pathways, or in an organismal context.

Additional interpretations emerged when we batch-processed fastq files (384–480 files/batch) in CollatedMotifs.py as for Genotypes.py. Within 5 min per 96-well plate, *collated_TFBS.txt* and *collated_TFBS.xlsx* files mapped lost or gained matches for up to 519 vertebrate TFs (JASPAR/JASPAR_CORE_2016_vertebrates, MEME) relative to inferred allele sequences; files documenting TFBS for 9216 samples were complete within 8 hrs. We found that despite the biallelic loss of the native GBS targeted at GOR *+*86.85 kb, the sequence change at one of the alleles reconstituted a novel, alternative GBS (Figure 5B and C, clone #1a). Moreover, we found in other examples that particular *FKBP5-*associated GOR alterations produced distinct regulatory consequences correlated with differential loss and/or gain of binding sites for non-GR TFBS motif matches. In one example, a mutant with bi-allelic deletions at GOR –26.65 kb appeared unaffected for *FKBP5* induction at 1 nM and 100 nM dexamethasone (Figure 5D, clone #2a), whereas another mutant homozygous for a single-bp insertion (+1 bp) at the same GOR showed nearly ablated *FKBP5* induction (Figure 5D, clone #2b; Supplementary Figure S14). Closer analysis revealed that the mutant with ablated *FKBP5* induction produced a unique gain-of-function that resulted in elevated pre-dex *FKBP5* transcript levels (Figure 5E).

Further evaluation with CollatedMotifs.py revealed that although both clone #2a and #2b lost the native GOR summit GBS as a consequence of Cas9 editing (Figure 5F, maroon arrows (‘lost GBS’) in top (clone #1) and lower (clone #2) panels), the homozygous insertion in clone #2 uniquely reconstituted a novel GBS (Figure 5F, red arrow (‘new GBS’) in lower panel). Moreover, clone #2b acquired sequence matches to Sox2 and DMRT3 binding motifs consequent to the +1 bp insertion within this GOR (Figure 5F, lower panel). Identification of the ‘new Sox2’ site (Figure 5F blue arrow in lower panel) associated with an *FKBP5* transcriptional phenotype is particularly interesting, as Sox2—a TF typically associated with stemness—is neomorphically overexpressed in many lung carcinomas (relative to adjacent normal lung tissue), including A549 (31,32).

These results highlight that even simple Cas9 edits can be associated with distinct transcription regulatory consequences, potentially illuminated by mutation-specific TFBS alterations that could render distinct functionalities (e.g. amorphic, neomorphic or inconsequential outcomes) to response elements under evaluation. Similarly, in other examples, small indels that successfully ablated the GBS native to the GOR summit commonly introduced a novel alternative GBS (across five *FKBP5* GORs and fourteen guide RNAs for which edited alleles were recovered, 14–60% of alleles that lost a native GBS reconstituted a novel substitute GBS, Supplementary Figure S15). As noted below, such novel GBSs produce altered contexts that may prove informative in studies that explore GBS sequence-selective GRE mechanisms.

## DISCUSSION

We have described three Python programs (https://github.com/YamamotoLabUCSF), which will be of value to researchers who prepare amplicons for targeted SBS on Illumina^®^ platforms: SampleSheet.py, Genotypes.py, and CollatedMotifs.py. These tools were developed in particular to facilitate analysis of edits targeted by programmable nucleases to genomic response elements—chromosomal regions that confer transcriptional regulation, each containing clusters of TFBSs potentially occupied by context-specific combinations of DNA sequence-specific TFs. The tools could also be useful in population studies that explore natural selection, sequence diversity, and allelic TFBS/co-TFBS coevolution.

### A tool set for TFBS analysis

Several other useful computational and experimental tools aid in the identification, processing, and analysis of altered genomic sequences. Dual-index barcoding system workflows (9,33) as well as computational analysis software packages (10–17) allow for aggregate analyses of populations of cells with sequence diversity by next generation sequencing. To our knowledge, however, the suite of programs and experimental procedures presented here is the only pipeline to use a dual-index deep sequencing approach to examine genomic sequence variations for up to 9216 samples for alterations in TFBS motifs in a single experiment. Identifying changes in TFBS landscape is an essential step in directly linking sequence variation with potential mechanism and phenotypic outcome. ENCODE (34) catalogues genome-wide positions for tens of thousands of these TFBS-rich putative response elements, but few such loci have been functionally validated *in vivo*. In principle, editing enables genetic analysis of sub-regional function within and across candidate response elements, including query of individual TFBSs; automated collation of lost and gained TFBSs can inform selection of clones for analysis, as well as guide hypotheses for further study.

### Tools to probe potential response element function

To validate the functionality of the suite of tools, we used a CRISPR/Cas9 approach, targeting GORs in A549 cells and generating hundreds of independent mutant clones. Although the specific position of a Cas9 cut can be predicted, the exact nature of the resulting indel cannot. It is therefore important to screen a large number of real edits to identify potentially informative mutant clones. Using the programs described here, we found that small (e.g. 1–5 bp) genomic edits in candidate response elements frequently changed not only the GR recognition sequence(s) targeted for genetic evaluation, but also, recognition motifs for other TFs. In some cases, small indels transfigured one GR recognition sequence (GBS) for another (Figure 5); precise sequence conservation of GBSs in many putative GREs implies that even simple transfigurations may be illuminating when examined in more complex cellular or signaling contexts. These results demonstrate that it is essential to define in detail the edits that occur in candidate response elements. Recent innovations in Cas9-directed editing, such as prime editing (35), which stipulates both target site and intended edit, should prove helpful, as sequence alterations delivered to candidate response elements can be pre-screened *in silico* for collateral TFBS alterations using CollatedMotifs.py.

### Applications to genome editing

Editing frequencies by programmable nucleases can vary dependent on locus, cell type, editing technology (e.g. TALENs versus CRISPR/Cas9), guide RNA sequence (for RNA-directed nucleases), and delivery mechanism (e.g. lipofection, nucleofection or viral delivery of DNA expression constructs or protein/ribonucleoprotein complexes), commonly demanding the screening of hundreds or thousands of candidates to identify rare clonal populations that harbor desired/desirable alleles, genotypes and zygosities (e.g. homozygous or biallelic mutants). Whereas small indels (1–10 bp, or allelic differences without base loss or gain) are not easily resolved by fragment analysis (such as by amplicon gel electrophoresis) or enzymatic assays, larger indels can be flagged by electrophoretic amplicon size differences; canvassing hundreds or thousands of amplicons is not amenable to high throughput, however, without specialized technologies such as the ZAG DNA Analyzer System (Agilent). Moreover, fragment analysis does not return allele identities, whereas the basis of screening in a deep sequencing approach is genotype definition. Genotypes.py excels at allele definition for a broad range of indel sizes: e.g. indels spanning ≤60 bp (intrinsic to the BLASTN algorithm) as well as indels approaching 100’s of bp (drawing on reference sequence spans retrieved by BLASTDBCMD), accomplishing genotype definition in concert with rare-clone screening.

### Employment of these resources outside of genome editing

While we employed our three new computational tools to a Cas9-editing workflow targeting candidate genomic response elements, the scripts are applicable to any scenario calling for locus-specific assignment of allele definitions and genotype inferences to individual members of a potentially diverse population, e.g. sequencing of single or multiple loci amplified from cell lines, tumor biopsies, cell-free DNA samples, viral passages, or individuals in a population. Illumina^®^ SBS is broadly amenable to paired-end sequencing of amplicons that canvas alleles with larger indel size variation than those described here (Supplementary Figure S8). We envision that SampleSheet.py and the 96 × 96 i5/i7 barcoded primers may be of broadest utility to users who sequence pooled, PCR-amplified material from large populations of discrete entities and wish to back-track sequence properties to their sources; that Genotypes.py may be useful for those who need rapid distillation of allele definitions and hypothesized genotype(s) for samples of known biological origin (i.e. with reference sequences available for alignment); and that CollatedMotifs.py may be of utility for users interested in an overview of TFBS differences resulting from genetic differences in experimental samples relative to a reference sequence, potentially aiding understanding of molecular phenotypes (hypothesis generation) or prioritization of clone choice/selection for further experimental analysis.

Open source program files, annotated Jupyter Notebooks, and Open Virtualization Format file for all code are available for download, enabling users to edit and tailor for customized goals, preferences and applications.

## DATA AVAILABILITY

Code for SampleSheet.py, Genotypes.py, and CollatedMotifs.py are available as annotated Jupyter Notebook (.ipynb) and program (.py) files at https://github.com/YamamotoLabUCSF, and pre-compiled in an Open Virtualization Format directory for virtualization at https://doi.org/10.5281/zenodo.3406861. Sample datasets analyzed in the current study are available as examples in the Zenodo repository https://doi.org/10.5281/zenodo.3406861. These resources include the list of sample:barcode assignments, fastq files, GRCh38 reference genome, fasta file of reference sequences, and TFBS file with position frequency matrices underlying these examples, along with sample output files; users can recapitulate generation of the Sample Sheet that demultiplexed reads from these clones as a test of SampleSheet.py, and for a subset of fastq files (corresponding to Cas9 edits targeted to GOR +86.85 kb) can recapitulate generation of the inferred genotypes and associated files as a test of Genotypes.py, and can recapitulate comparison of TFBS motifs as a test of CollatedMotifs.py. Raw fastq and processed (bigWig) GR ChIP-seq datasets from which *FKBP5* GORs were identified in A549 are available in the NCBI Gene Expression Omnibus (GSE163398).

## Supplementary Material

gkab700_Supplemental_Files

## Notes

Present address: Matthew T. Knuesel, HudsonAlpha Institute for Biotechnology, 601 Genome Way Northwest, Huntsville, AL 35806, USA.
