# Supplementary material for: Computational resources to define alleles and altered regulatory motifs at genomically edited candidate response elements
Source: Nucleic Acids Res. 2021 Aug 20;49(16):9117–31. doi: 10.1093/nar/gkab700 (PMC8450113; doi:10.1093/nar/gkab700)
Supplement: gkab700_Supplemental_Files [file gkab700_supplemental_files.zip › SupplementaryMaterial.pdf]

Supplementary Material

Computational resources to define alleles and altered regulatory motifs at genomically edited candidate response elements

Corresponding authors: Keith R. Yamamoto, Kirk Ehmsen

Tel: +1 415 476-8445; Fax: +1 415 514-4112;
Email: yamamoto@ucsf.edu, kirk.ehmsen@gmail.com

Supplementary Guidelines

A. *Overview: where & how can these code resources be accessed?*

The three computational resources (SampleSheet.py, Genotypes.py, CollatedMotifs.py) are available for download from open-access repositories at **GitHub** (Jupyter Notebook, Python program file) or **Zenodo** (Open virtualization format for virtual machine, *e.g.*, Oracle VM VirtualBox).

A.1. To access **Jupyter Notebook** (.ipynb) or **Python program** (.py, command-line) files for Mac or Windows OS, visit:

> **https://github.com/YamamotoLabUCSF**
>
> Detailed instructions for **System Setup** (Python 3 and Jupyter Notebook installation, GitHub repository access/file download, creation of Python virtual environment containing Python package(s) required for code operation) and **Code Launch** are provided in the **README.md** file associated with each code repository. **Sample test files** (input files to test each code tool) are available for download from Zenodo ('ExampleTestFiles' directory) at: **https://zenodo.org/record/3406861 (DOI 10.5281/zenodo.3406861)**

A.2. To access an **Open virtualization appliance** (.ova, virtual machine) with program dependencies pre-installed for Mac or Windows OS (titled, '**Alleles_and_altered_motifs**'), visit:

> **https://zenodo.org/record/3406861 (DOI 10.5281/zenodo.3406861)**
>
> **Alleles_and_altered_motifs** is an Open virtualization appliance (.ova) file that composes a virtual machine (VM), pre-packaged with SampleSheet.py, Genotypes.py, CollatedMotifs.py, and their dependencies (*e.g.*, BLASTN, BLASTDBCMD, MAKEBLASTDB, FIMO, FASTA-GET-MARKOV).
>
> *Note: if you wish to use CollatedMotifs.py on a machine running Windows OS, the open virtualization format is the best choice because FIMO (MEME suite) is not natively supported by Windows.*

- First download, install and launch Oracle VM VirtualBox (free, https://www.virtualbox.org/) or another virtualization software.
- To open 'Alleles_and_altered_motifs' in Oracle VM VirtualBox, select "Import Appliance" from "File" menu and follow prompts.
- Client-specific setting recommendations:
    - *Motherboard:* Base Memory default is 4 GB. User should set this to at least 4 GB, or double the size of your largest fastq dataset. Note, this RAM will not be available to the host computer while the VM is running.
    - *Processor:* default is 2 CPU. The VM is compatible with multiple CPUs, but the VM CPU number should not exceed n-1 (1 CPU lower than the total host system CPU number).
    - *General note:* the .ova file is ~20 GB, but will require 40 GB free space on the host computer to run.
    - *Note to Mac users*: name the VM and select USB 3 compatibility in Settings during import process.
- User and Password:
    - *User (automatic):* Altered Motif / *Password:* Motifs

B. *Usage notes*

These guidelines offer notes regarding (a) Python library dependencies, (b) other dependencies, (c) detailed instructions for user inputs, and (d) brief 'core operation' comments for each of the three computational tools, *SampleSheet.py*, *Genotypes.py*, and *CollatedMotifs.py*.

B.1. SampleSheet.py

B.1.a. SampleSheet.py—Python dependencies

SampleSheet.py relies on users to input integer value(s) (1-96) that correspond to specific well position(s) (A01-H12) of 96-well plates, ultimately linked to unique i7 and i5 barcode sequences used to label DNA samples for sequencing (see **Fig. 2, Supp. Fig. 1, Supp. Fig. 5**). The script offers '*plateviews*'—96-well plate perspectives on relationships between i7, i5 barcode sequences and barcode stock plate well positions—to help establish appropriate relationships between user input and desired specification of barcode sequence(s) in a Sample Sheet (see **Supp. Fig. 6**).

In command-line interface (CLI) format, *display of these console plateviews is optional, but if display is preferred, this requires prior installation of* ***PrettyTable***, a Python library that supports visual representation of tabular data (freely downloaded from the Python Package Index (PyPI) at https://pypi.org/project/PrettyTable/ or from GitHub at https://github.com/jazzband/prettytable); in Jupyter Notebook format, *plateviews are automatically presented* as images returned after user-definition of variables.

In CLI format, SampleSheet.py checks for PrettyTable installation in the system path; a user can choose to bypass PrettyTable at a console prompt that queries whether the user wishes to progress in the script in its absence if PrettyTable cannot be found. Users are encouraged to create a **local Python virtual environment** into which PrettyTable is installed and in which SampleSheet.py/SampleSheet.ipynb can be run (guidelines for creating this virtual environment using the **SampleSheet_requirements.txt** can be found under 'System Setup' in the SampleSheet repository's README.md file).

B.1.b. SampleSheet.py—other dependencies

None.

B.1.c. SampleSheet.py—user inputs and specification of sequencing run properties

SampleSheet.py prompts users for six values, entered as text at individual Jupyter interface or CLI prompts (**Fig. 2a**, **Supp. Table 2**, **Supp. Fig. 2**, **Supp. Fig. 3a**). These values include: 1) Illumina® Indexed Sequencing Workflow (A *vs*. B), 2) absolute path to output directory and filename for Sample Sheet, 3) Investigator Name, 4) Project Name, 5) Single-end (SE) or Paired-end (PE) sequencing run with cycle number(s), and 6) list of sample:barcode relationships.

> *B.1.c.i. Barcode sequence orientation in SampleSheet—Workflow 'A' vs. 'B'.* Illumina® indexed sequencing uses two different paired-end Indexed Sequencing Workflows, depending on sequencer (**Supp. Fig. 4**). In *Workflow A*, index 2 is sequenced before read 2 resynthesis, meaning that i7 is sequenced as the reverse complement and i5 is sequenced on the forward strand (applicable to NovaSeq 6000, MiSeq, HiSeq 2500, HiSeq 2000); in *Workflow B*, index 2 is sequenced *after* read2 synthesis, creating the reverse complement of both index1 (i7) and index2 (i5) (applicable to iSeq100, miniSeq, NextSeq, HiSeq X, HiSeq 3000, 4000).

This distinction requires attention to barcode sequence entry in Sample Sheet fields 'index1' and 'index2'; users of SampleSheet.py must verify the index sequencing workflow on the sequencer on which they will load their libraries, as SampleSheet.py fills barcode sequences based on specified workflow (**Fig. 2a**, **input #1**; **Supp. Fig. 3a-1**; see **Supp. Fig. 4** for schematic of Workflows A & B).

B.1.c.ii. Absolute path to file output. Users are next prompted for a text string that specifies the absolute path to a location where the Sample Sheet file will be created (**Fig. 2a**, **input #2**; **Supp. Fig. 3a-2**); this string must be entered as a series of directory name(s) beginning at the file system root (*e.g.*, /Users on Mac, C:\ on Windows operating systems), ending in the file name to be created by the script (*e.g.*, SampleSheet.csv). Console prompts specify that regardless of the operating system (OS, *i.e.*, Mac, Linux, or Windows), directory names must be separated by forward slashes (/); functions in the Python *operating system* module generate OS-appropriate paths from the user-provided string.

The remaining five user-entered values populate Python variables that are printed to Sample Sheet sections to customize content (detailed below as *workflow specifications* and *sample:barcode assignments*). Sample Sheets require three sections—denoted in the *.csv file by the bracketed strings [Header], [Reads], [Data]—and optionally include additional sections (*e.g.*, [Settings], [Manifests]). SampleSheet.py generates Sample Sheets that use four of these sections: [Header], [Reads], [Settings], [Data] (**Supp. Fig. 2**).

B.1.c.iii-vi. Workflow specifications. **[Header]**—[Header] and [Settings] demarcate lines of comma-separated key:value pairs that encode metadata for the sequencing experiment; each key denotes a metadatum type and each value encodes a corresponding metadatum. SampleSheet.py prints values for eight metadata keys under [Header]: *IEMFileVersion, Investigator Name, Project Name, Date, Workflow, Application, Description*, and *Chemistry*. Values for two keys (*Investigator Name, Project Name*) are user-supplied at console prompts during script operation (**Fig. 2a, inputs #3-4**; **Supp. Fig. 3a-3**), with Date value auto-generated based on the system's present calendar time. Five keys default to values appropriate for amplicon sequencing on Illumina® instruments

(*"IEMFileVersion,4", "Workflow,GenerateFASTQ", "Application,FASTQ Only", "Assay,Nextera", "Description, Sequencing",* and *"Chemistry, Amplicon"*).

[Reads]—The number of nucleotide-step extension and imaging cycles to be completed by the sequencer is specified by numeric values in lines of a Sample Sheet's [Reads] section: a single line (*e.g.*, 151) communicates that 150 cycles of base acquisition (following a +1 phasing cycle) will be completed to generate a single read (single-end run); two lines (*e.g.*, 151 \n 151, where '\n' represents newline character) communicates that 150 cycles will be completed in two directions to generate forward and reverse-complement reads (paired-end run). Users enter the read cycle number(s) to be printed in the Sample Sheet [Reads] section as a single line of comma-separated text (two or three values) at the console prompt, specifying single-end or paired-end run (SE or PE) and cycle number(s) (single number for SE run, two numbers for PE run) (**Fig. 2a, input #5**; **Supp. Fig. 3a-4**). For example, for a single-end run with 35 read cycles (plus a single phasing cycle), a user would enter *SE, 36*. For a paired-end run with 150 read cycles in each direction, a user would enter *PE, 151, 151*.

[Settings]—The 96x96 oligonucleotides—having 8-nt index sequences embedded in SampleSheet.py data objects—are designed with Nextera sequences flanking the target read sequence (**Supp. Fig. 1**) (1), meaning that the adapter sequence *5'-CTGTCTCTTATACACATCT-3'* defines the end of amplicon read sequences. SampleSheet.py populates the optional [Settings] section of the Sample Sheet with the key:value pair (*"Adapter,CTGTCTCTTATACACATCT")* that signals adapter trimming to take place during fastq processing. *"ReverseComplement,0"* specifies that read sequences are returned as sequenced, not as reverse complements.

B.1.c.vii. Sample:barcode assignments. **[Data]**—The final prompt for user input requests a list of sample names (*e.g.*, overarching sample names uniquely assigned to each 96-well plate) that are comma-separated from integers that specify i7 and i5 barcode assignments to wells (**Fig. 2a, input #6**; **Supp. Fig. 3a-5**). Each line defines a plate name that will be shared across associated well IDs (*e.g.*, A01-H12) , range of i7 barcodes, and single i5 barcode that uniquely encompass up to 96 barcoded samples in arrayed format. For example, in the following six lines of text,

DG-1, 1-96, 1

DG-2, 1-96, 9
DG-3, 1-50, 78
DG-4, 1-96, 7
DG-5, 1-96, 22
DG-6, 1-68, 34

'DG-1' represents a sample plate name, '1-96' represents the range of i7 barcodes used in PCR2 (*i.e.*, all 96 i7 primers/barcodes were used (A01-H12), each uniquely labeling a distinct and corresponding well (A01-H12) within the sample plate), and '1' represents the single i5 primer/barcode (from i5 source plate well A01) used to label all wells within this sample plate. SampleSheet.py anticipates for i7 primers to be repeatedly used across 96-well plates to specify individual wells within each sample plate, and an i5 primer to be uniquely assigned to each sample plate to specify the overarching source of i7-labeled wells within each plate. In CLI format, input can be entered line-by-line by a user until a list of entries is complete (a single newline keystroke advances for entry of next sample and barcode range; two consecutive newline keystrokes complete list entry advance the script), or as a single block copied and pasted from advance preparation in a text editor. In Jupyter Notebook format, input must be entered with each line entry separated from others by a semicolon character (';', see Jupyter Notebook Markdown (https://github.com/YamamotoLabUCSF/SampleSheet) for details).

B.1.c. SampleSheet.py core operation comments

To facilitate mapping of the relationships between the numbers 1-96, well IDs between A01-H12, and corresponding barcode sequences, the script displays console table-views of the i7 and i5 barcode sequences ("plateviews"),. These plateviews provide resources to help construct accurate sample ID:barcode assignments at the key [Data] user input step in the program (**Supp. Fig. 1**). Screen captures of the console plateviews meant to facilitate barcode ID entry can also be found in **Supp. Fig. 6** (CLI and Jupyter Notebook formats); see *B.1.a—Python dependencies* for additional detail.

SampleSheet.py understands relationships between i7, i5 well ID and barcode sequence, with expectation of i7 barcodes defining wells and i5 barcode defining plate (**Fig. 2b,c**). From this minimal syntax for sample plate IDs, i7, and i5 barcodes, for example, the six lines of text in the example above are converted to 502 entries in Sample Sheet format; each sample plate is 'expanded' to delineate up to 96 individual wells

based on the minimal information provided in the i7/i5 range(s) provided as input (*e.g.*, DG-1-A01, DG-1-A02, … DG-1-H12, etc.) (**Fig. 2c-d**).

B.2. Genotypes.py

B.2.a. Genotypes.py—Python dependencies

Generation of read frequency statistics requires installation of Python **NumPy** and **SciPy** libraries (https://www.scipy.org/scipylib/download.html, https://pypi.org/project/numpy/). Generation of a PDF file with allele frequency plots (optional) requires Python **fpdf** and **PyPDF2** packages (https://pypi.org/project/fpdf/, https://pypi.org/project/PyPDF2/); allele frequency plots can be optionally bypassed during script operation (in which case the fpdf and PyPDF2 dependencies can be ignored). Users are encouraged to create a **local Python virtual environment** into which these Python dependencies are installed and in which Genotypes.py/Genotypes.ipynb can be run (guidelines for creating this virtual environment using the **Genotypes_requirements.txt** can be found under 'System Setup' in the Genotypes repository's README.md file).

B.2.b. Genotypes.py—other dependencies

- **BLASTN** is required for read alignments to a reference sequence database (see *B.2.c.iii* below) (2);
- A **reference sequence alignment database** (set of six files with a common prefix (*e.g.*, 'GRCh38') and extensions .nhr, .nin, .nog, .nsd, .nsi, .nsq) is required by BLASTN for sequence alignment; an alignment database for specific reference sequence(s) can be user-generated in advance from a custom fasta file containing one or more fasta-formatted sequence(s), or a database for a full organismal genome or transcriptome (*e.g.*, human genome assembly GRCh38 (NCBI Genome Reference Consortium, h38)) can be generated in advance from a fasta file containing the entire organismal sequence collection (see *B.2.c.iv* and *B.2.c.v* below). A pre-existing alignment database for human genome assembly GRCh38 (NCBI Genome Reference Consortium, h38) can be found in 'ExampleTestFiles' at: https://zenodo.org/record/3406861, DOI: 10.5281/zenodo.3406861;

- **BLASTDBCMD** is required for sequence retrieval from the reference sequence database (above), to enable hypothesized allele reconstitutions between high-scoring pairs (hsp's) within a 1-kb span, (hsp's output by BLASTN; see *B.2.c.vi* below).

B.2.c. Genotypes.py—user inputs

Genotypes.py prompts users for up to eight values—six required and two optional—entered as text at Jupyter Notebook or CLI prompts (**Fig. 3**, **Supp. Table 3**). These include: absolute paths to 1) input and 2) output directories, 3) BLASTN executable, and 4) alignment reference database; 5) reference database file prefix; 6) absolute path to BLASTDBCMD executable; and (optional) 7-8) DNA sub-sequences to display on alignments. At the outset of Genotypes.py, a user can choose whether to enter input values at 'coached' prompts ('Prompt') or in a single entry ('List') that is parsed by the script into appropriate variables. Coached prompts provide descriptive context for properties of each input, with values entered one at a time by a user. List entry conveniently allows a single list of the variables to be pasted into the input prompt all at once.

B.2.c.i. Absolute path to file output. Users are first prompted to enter the location of a directory for output files (absolute path to target destination, empty of files) (**Fig. 3a, input #1**; **Supp. Fig. 3b-1**). The directory can either pre-exist (as long as it is empty), or does not have to pre-exist (the script will create the directory designated by the absolute path if it does not yet exist). Up to eight files (six .txt, one .pdf [optional], one .csv) will ultimately be generated in this directory as script output (**Supp. Table 4**).

B.2.c.ii. Absolute path to file input. The script then requests location of the source file directory—a directory populated with fastq files containing reads derived from amplicon sequencing (fastq files can be supplied to Genotypes.py compressed (with *.gz suffix) or decompressed (with *.fastq extension)) (**Fig. 3a, input #2**; **Supp. Fig. 3b-2**). Fastq files can be pre-processed by read quality-filtering tools (*e.g.*, *sickle* (3), *cutadapt* (4), *trimmomatic* (5), *fastp*(6)), or delivered to the script directly without pre-processing.

B.2.c.iii. Absolute path to BLASTN executable. Genotypes.py aligns (up to) the top 10 reads (abundance defined by frequency) from each fastq file to a reference genome using BLASTN, requiring local pre-installation of BLASTN (2) (**Fig. 3a,**

input #3; **Supp. Fig. 3b-3**; freely available for download with the BLAST+ suite at https://www.ncbi.nlm.nih.gov/guide/howto/run-blast-local/).

B.2.c.iv. Absolute path to reference sequence (e.g., genome) database. BLASTN requires a local reference sequence database for alignment operations, typically a set of six files with a common prefix (*e.g.*, 'GRCh38') and extensions .nhr, .nin, .nog, .nsd, .nsi, .nsq, generated from a single fasta file containing one or more entries (for example, in the case of a database source file GRCh38.p13_genomic.fna, 457 fasta entries; RefSeq ID: GCF_000001405.39; https://www.ncbi.nlm.nih.gov/assembly/GCF_000001405.39) (7) (**Fig. 3a, input #4**; **Supp. Fig. 3b-4**). A user-specified genome database (or customized sequence database) is a single directory containing these six files, and can be made by supplying a fasta file containing the target sequence(s) from which BLASTN will seek alignments to MAKEBLASTDB, a CLI program available in the BLAST+ download suite (usage guidelines described in the BLAST Command Line Applications User Manual , https://www.ncbi.nlm.nih.gov/books/NBK279688/). *Note:* A pre-existing alignment database for human genome assembly GRCh38 (NCBI Genome Reference Consortium, h38) can be found in 'ExampleTestFiles' at: https://zenodo.org/record/3406861, DOI: 10.5281/zenodo.3406861.

B.2.c.v. Prefix common to the six files that compose the alignment reference database. The alignment reference database comprises six files with a common prefix; the script requests this prefix from the user (**Fig. 3a, input #5**; **Supp. Fig. 3b-5**). See **Supp. Table 3** ("Database prefix") or **Supp. Fig. 10** ("alignment database" files (example from CollatedMotifs.py)) for examples (in the CollatedMotifs.py database example, the prefix would be *FKBP5_GOR+86.848kb*).

B.2.c.vi. Absolute path to BLASTDBCMD executable. Genotypes.py relies on the BLASTN 'basic local alignment' algorithm for read alignments to a reference genome. Because BLASTN excels at local alignment, deletions exceeding ~60 nt are separated into two separate high-scoring pairs (*hsp*'s: associated with the same accession ID from the BLASTN database, but distinguished by separate database coordinate spans and alignment query, hit, and midline fields in the BLASTN output) (2). In addition to channeling conventional BLASTN alignment hits (with

a single hsp identified for a Ranked Allele / “read”) into code steps that accomplish allele definition and genotype inferences, Genotypes.py also specifically identifies reads with alignments having >1 hsp; based on defined criteria (non-overlap of hsp’s and total coordinate span across hsp’s (including any gap between them) within 1 kb), Genotypes.py calls upon BLASTDBCMD to retrieve the corresponding DNA sequence spanned by the hsp’s (end-to-end) and reconstitutes hypothesized alleles as single patched alignments that would otherwise be split as >1 alignment pair (missing intervening reference sequence). These ‘reconstituted’ alignments and associated alleles are pooled with the “single-hit, single-hsp” alignment cohort available directly from BLASTN, allowing alleles ranging from mismatches and short indels (BLASTN strengths) to long indels (100’s of bp, if sequencing amplicon has straddled a deletion or insertion relative to reference sequence) to be channeled into the allele definition and genotype deduction steps of Genotypes.py (BLASTDBCMD: **Fig. 3a, input #6**; **Supp. Fig. 3b-6**; freely available for download with the BLAST+ suite at https://www.ncbi.nlm.nih.gov/guide/howto/run-blast-local/).

B.2.c.vii & viii. Optional DNA subsequence(s). Users have the option to supply one or more short nucleotide sequences to be mapped/superimposed above or below allele alignments, if matches are found in the aligned nucleic acid sequences. A Jupyter Notebook or CLI prompt first asks whether a user will supply entries for one or both of up to two optional subsequence inputs; these include: 1) ‘guide RNA sequence’ (5’→3’, in DNA form, excluding PAM), if user wishes to display position of guide RNA used in Cas9 editing effort (can be useful to gauge the plausibility of an allelic difference relative to reference sequence as being consequence of Cas9-induced break) (**Fig. 3a, input #7; Supp. Fig. 3b-7**), and 2) ‘test sequence’ (5’→3’) (also referred to as ‘extant sequence’ or ‘sequence of interest’) if user wishes to query for presence or absence of a specific subsequence (such as the original TFBS targeted for ablation) in an allele identified by deep sequencing (relative to a reference, *e.g.*, wild-type, allele) (**Fig. 3a, input #8**; **Supp. Fig. 3b-8**).

B.2.d. Genotypes.py—core operation comments

B.2.d.i. Fasta.fa—Core operations begin with fastq file processing, channeling the top ten ranked read types and their quantified frequency metrics to a fasta text file

(*fasta.fa*), the input for BLASTN alignments. These frequency-ranked reads are "*Ranked (candidate) Alleles*" (referred to as "Ranked Alleles")—up to ten ranked read types are presented to Genotypes.py for alignment, allele definition, and contribution to genotype inference based on allele type and relative abundance. The rationale for ten ranked alleles is to facilitate user corroborations of inferred genotypes, because common read types will occur at much higher abundance than PCR or sequencing artifacts, allowing a clear 'drop-off' in ranked allele frequencies between likely representative alleles (homozygous, heterozygous, or multizygous) and trivial reads unreflective of genotypic source material. For each sample, every read sequence is collected in a temporary Python list (*read_lines*), evaluated by the Python Counter function to identify the top ten most represented reads and their frequency metrics expressed in five ways: 1) read count/total sample reads (ranked allele reads over total sample reads), 2) read percentile rank relative to other reads, 3) % read abundance (raw), 4) % read abundance relative to reads that occur at >1% frequency, 5) % read abundance relative to reads that occur at >10% frequency (**Fig. 3b**). The fasta description line (defline) for each read sequence ingrains both sample ID and frequency metrics (sample ID = Sample_Name defined in Sample Sheet [Data] section and fastq file name, see *B.1.c.vii. Sample:barcode assignments*), embedding values used in upcoming script operations to assess read contribution to genotype inference (defline structure: *>samplename-plate-well_R1orR2_[read count/total reads for sample]_rank#_% of all reads_percentile rank relative to all reads_% of all reads adjusted for reads that occur >1%_% of all reads adjusted for reads that occur >10%*) (**Fig. 3c**). This defline is the ranked allele 'name'/identifier tracked in all Genotypes.py processing steps ahead.

<u>Notes</u>:

- *Genotypes.py does not filter for Phred (Q) quality score across reads or for minimal read lengths. Users are encouraged to assess quality score distribution across reads in fastq files using FASTQC* (8)*, and if desired, trim or filter reads based on desired criteria (such as average >Q20 across read) using a program such as sickle, cutadapt, trimmomatic, or fastp.*
- *Even though Genotypes.py initially collects up to ten frequency-ranked reads ("Ranked Alleles"), one or more Ranked Alleles may be deprecated (removed from allele definition and decommissioned from contribution to genotype*

inference) during subsequent script operations if 1) the 'read' does not align to the reference genome (no hit) or maps to multiple hits (BLASTN), 2) the read has >1 hsp (BLASTN) but is not amenable to BLASTDBCMD sequence retrieval because the hsp alignments overlap one another, 3) the read has >1 hsp but the hsp's span >1 kb (Genotypes.py assigns this as an implausible reconstruction, as the script does not anticipate amplicons >1 kb in length to have been submitted to Illumina sequencing). Identities of deprecated Ranked Alleles are available in the output file, population_summary.txt.

B.2.d.ii. Blastn_alignments.txt—The script passes *fasta.fa* to BLASTN using Python's System Command function, accessing user-specified paths to the BLASTN executable (*input #3*), the reference sequence database directory (*input #4+input#5*), and *blastn_alignments.txt* with output settings *-gapopen 1 -gapextend 1 -outfmt 5*.

Genotypes.py populates the alignment content of *blastn_alignments.txt* into a series of Python list objects, in which alignment data are parsed and reformatted (*e.g.*, filtered of queries flagged by 'No hits found' and queries that identified multiple hits ('<Hit num>' >1) in the reference database). To account for high-scoring pairs (hsp's) that may span a sizeable indel (*e.g.*, >~60 bp) and are split by BLASTN into separate alignments, Genotypes.py specifically earmarks reads with >1 hsp that 1) occur within 1 kb of one another (end-to-end), and 2) do not overlap (allowing reconstitution by referral to the reference sequence they span); Genotypes.py recruits these reads for allele construction using BLASTDBCMD to retrieve the intervening reference sequence to reconstitute an hsp-spanning alignment (see *B.2.c.vi. Absolute path to BLASTDBCMD executable*). Alignments from both BLASTN and BLASTDBCMD-aided reconstruction are then pooled for allele definition and genotype deduction. Query sequences that belong to the same sample ID (sequences among the top ten ranked reads for a sample) are grouped and assigned to unique sample ID in a dictionary, *alignmentoutput_dict2*.

Genotypes.py draws from *alignmentoutput_dict2* to populate its core dictionary, *genotypes_dict*, in which data are ultimately evaluated for sample allele definitions and genotype deduction. For each sample, *genotypes_dict* compiles four subdictionaries assigned to each ranked sequence (candidate allele): subdictionary 1 records 'allele_name' (fasta defline), 'chr+build', 'locusID',

‘coordinates’, and ‘alignment’; subdictionary 2 records ‘allele_type’ (*e.g.*, wild-type, mutant) and ‘allele_specs’ (‘specifications’, *e.g.*, likely deletion, insertion, substitution, indel), subdictionary 3 records guide RNA sequence(s) with match position in reference sequence, and subdictionary 4 records DNA test sequence(s) with match position in reference sequence. Finally, inferred genotype—based on ranked allele type and specification for alleles with >10% adjusted frequency—is assigned to each sample ID (*e.g.*, homozygous wild-type, homozygous deletion, heterozygous deletion, multi-allelic, etc.). These key outputs are recorded in allele_definitions.txt, genotypes.txt, allele_evidence.pdf, and allele_definitions.csv (**Supp. Fig. 9**, **Supp. Table 4**). Population-level summaries of aggregate allele types and genotype inferences assigned across all samples is available in *population_summary.txt* (**Supp. Fig. 9**, **Supp. Table 4**).

B.2.d.iii. Script_metrics.txt—Genotypes.py logs script operation parameters in *script_metrics.txt*, preserving *i)* operating system information (name, platform, RAM (GB), physical CPU/effective CPU, Python executable), *ii)* user-defined variables (output_directory, fastq_directory, blastn_path, db_path, db_prefix, blastdbcmd_path, guideRNA_seq, extant_seq), fastq file properties (*e.g.*, Illumina run ID(s), # of fastq files processed and their size and read distribution), *iii)* file output information (output directory, files and their sizes), and *iv)* script operation times (*e.g.*, start time, fasta processing time, alignments processing time, inference processing time, frequency plots compilation time, etc.) (**Supp. Fig. 9**).

B.3. CollatedMotifs.py

B.3.a. CollatedMotifs.py—Python dependencies

Generation of read frequency statistics requires installation of Python **NumPy** and **SciPy** libraries. Users are encouraged to create a **local Python virtual environment** into which these dependencies are installed and in which CollatedMotifs.py/CollatedMotifs.ipynb can be run (guidelines for creating this virtual environment using the **CollatedMotifs_requirements.txt** can be found under ‘System Setup’ in the CollatedMotifs repository’s README.md file).

B.3.b. CollatedMotifs.py—other dependencies

Note: the MEME suite (9) is incompatible with Windows OS. Windows users are encouraged to use the **Alleles_and_altered_motifs.ova** open virtualization format file found in "Resources to accompany 'Computational resources to define alleles and altered regulatory motifs at genomically edited candidate response elements'", at https://zenodo.org/record/3406861, DOI: 10.5281/zenodo.3406861).

- **BLASTN** is required for read alignments with a reference sequence database (see *B.3.c.iv* below) (2);
- **MAKEBLASTDB** is required for automatic construction of a reference sequence alignment database based on user-provided reference sequence(s) (see *B.3.c.v* and *B.3.c.vi* below);
- **FIMO** is required for TFBS motif searches in inferred alleles (see *B.3.c.vii* below) (10);
- A **TFBS motif database (MEME format)** is required to specify TFBS motif matches identified and scored (p-value) by FIMO (see *B.3.c.viii* below);
- **FASTA-GET-MARKOV** is required to estimate a Markov model of sequence probabilities (background model), from which p-value scores for TFBS motif matches are assigned by FIMO (see *B.3.c.ix* below);
- A fasta-formatted **reference sequence for FASTA-GET-MARKOV background model generation** is required, from which FASTA-GET-MARKOV will generate a Markov model provided to FIMO for p-value assignments to TFBS motif matches (see *B.3.c.x* below).

B.3.c. CollatedMotifs.py—user inputs

CollatedMotifs.py prompts users for eleven values (ten required, one optional)—nine absolute paths to directories, files, or executables, plus one prefix for alignment database files and (optional) the unique name of a transcription factor (TF) of interest that occurs in the positional weight matrix file provided to FIMO—entered as text at Jupyter Notebook or CLI prompts (**Fig. 4**, **Supp. Table 5**).

B.3.c.i. Absolute path to output directory. Users are first prompted to enter the location of a directory for output sub-directories and files (absolute path to target destination, empty of directories and files) (**Fig. 4a, input #1**; **Supp. Fig. 3c-1**). The directory can either pre-exist (as long as it is empty), or will be created by the script if it does not yet exist. Three sub-directories (*alignment_database,*

fimo_out and *fimo_out_ref*) and six files will ultimately be generated in this directory as script output (**Supp. Table 6**).

B.3.c.ii. Absolute path to fastq files. The script then requests location of the source file directory—a directory populated with sample-specific fastq files containing reads to be processed for sequence content and matches to TFBS motifs (**Fig. 4a, input #2**; **Supp. Fig. 3c-2**). Fastq files can be pre-processed by read quality-filtering tools (*e.g.*, *sickle* (3), *cutadapt* (4), *trimmomatic* (5), *fastp*(6)), or delivered to the script directly without pre-processing.

B.3.c.iii. Absolute path to reference fasta file. CollatedMotifs.py relies on user-supplied reference sequence(s) to evaluate read (allele) sequence properties, specifically to 1) run sequence alignments and 2) compare TFBSs. Users supply reference sequence(s) (≥1) in NCBI fasta format in a single fasta-formatted text file (**Fig. 4a, input #3**; **Supp. Fig. 3c-3**). The span of each user-supplied reference sequence ideally should correspond to the full genomic amplicon prepared for deep sequencing (borders defined by 5'-most complementarity of primers to target sequence), although the script can accommodate reference sequence spans that exceed the borders of the sequenced amplicons (*e.g.*, padded on 3' and/or 5' end(s)); *CRITICAL*: each associated fasta defline should be designated by a *useful name or string* that can be matched in entirety in fastq sample name(s) scheduled to be compared to the fasta reference sequence in question (for example, for sample fastq filenames containing the shared prefix 'DG-1' (*e.g.*, DG-1-A01, DG-1-A02, … DG-1-H12), the fasta defline for the reference sequence assigned to DG-1 reads should appear as, '>DG-1'). Any extra characters in the fasta defline (*e.g.*, >DG-1*_reference*) will lead the script to be unable to pair with the sample(s) for TFBS comparisons ('*_reference*' does not occur in the fastq filename, so script will not recognize this fasta entry as the reference match to DG-1 samples for TFBS comparison). CollatedMotifs.py provides these sequences to MAKEBLASTDB to generate a custom sequence database for alignments, and also to FIMO, to generate reference-specific TFBS lists for allele comparisons.

B.3.c.iv. Absolute path to BLASTN executable. CollatedMotifs.py aligns the top 5 reads (abundance defined by frequency) from each fastq file to a reference

sequence database (*alignment_database*) using BLASTN (2) (available for download with BLAST+ suite) (**Fig. 4a, input #4**; **Supp. Fig. 3c-4**).

B.3.c.v. Absolute path to MAKEBLASTDB executable. BLASTN requires a local sequence database for alignment operations, a set of six files generated from a single fasta file containing one or more entries (see reference sequence database entry in Genotypes.py description for further detail, *B.2.c.iv. Absolute path to reference sequence (e.g., genome) database*) (7). Whereas a user of Genotypes.py prepares a genome sequence database using the CLI program MAKEBLASTDB in advance of script operation, in CollatedMotifs.py users provide the absolute path to the MAKEBLASTDB executable within BLAST+ suite (**Fig. 4a, input #5**; **Supp. Fig. 3c-5**); the script invokes MAKEBLASTDB to generate a database from the sequences in the user-supplied *reference fasta file* (*input #3* / see *B.3.c.iii*). For database example, see **Supp. Fig. 10a:** *alignment_database*.

B.3.c.vi. Prefix common to the six files that compose the alignment reference database. The alignment reference database comprises six files with a common prefix; the script requests a user-defined (custom) prefix to be assigned to these files by MAKEBLASTDB (**Fig. 4a, input #6**; **Supp. Fig. 3c-6**). See **Supp. Table 3** or **Supp. Fig. 10** for examples (in the CollatedMotifs.py database example, the prefix would be *FKBP5_GOR+86.848kb*).

B.3.c.vii. Absolute path to FIMO executable. To identify matches to TFBS motifs, CollatedMotifs.py invokes FIMO ('Find Individual Motif Occurrences'), a program available in the MEME suite of motif-based sequence analysis tools (MEME 5.0.5 download available at http://meme-suite.org/doc/download.html, FIMO background available at http://meme-suite.org/doc/fimo-tutorial.html) (**Fig. 4a**, **input #7**; **Supp. Fig. 3c-7**) (9, 10). Users supply the absolute path to the local installation of the FIMO executable. Note that in Windows OS, MEME suite programs require virtualization, and CollatedMotifs.py must be run from within a hypervisor (*e.g.*, Oracle VirtualBox available at https://www.virtualbox.org). The Open Virtualization Format file preloaded with SampleSheet.py, Genotypes.py, CollatedMotifs.py and all of their dependencies is available at https://zenodo.org/record/3406861, DOI: 10.5281/zenodo.3406861.

B.3.c.viii. Absolute path to FIMO motif file. The TFBS search program FIMO uses a plain-text file containing position frequency matrices for one or more TFs (MEME format), the basis of TFBS identification in user-supplied reference sequences and fastq-supplied (sample 'query') sequences. Users can download a directory of motif database files at http://meme-suite.org/doc/download.html (**Fig. 4a, input #8**; **Supp. Fig. 3c-8**). Dozens of files listing position frequency matrices experimentally defined for TFs in eubacteria, archaea, and eukaryotic groups are available in the *motif_databases* directory of a MEME suite download. We used *JASPAR/JASPAR_CORE_2016_vertebrates.meme*, from the 2016 (6th) release of the public database JASPAR (11), as the motif reference file in the example case (containing position frequency matrices for 519 vertebrate TFs).

B.3.c.ix. Absolute path to FASTA-GET-MARKOV executable. FIMO requires a background model from which to assess statistical significance of sequence matches to position frequency matrices; CollatedMotifs.py invokes FASTA-GET-MARKOV, a program available within MEME suite download, to generate a background model for FIMO operations on evaluated sequences. FASTA-GET-MARKOV generates a background Markov model from a user-supplied *reference fasta file* (*input #10* / see *B.3.c.x*) (further description at http://meme-suite.org/doc/fasta-get-markov.html) (**Fig. 4a, input #9**; **Supp. Fig. 3c-9**).

B.3.c.x. Absolute path to file supplied to FASTA-GET-MARKOV for background model generation. FASTA-GET-MARKOV produces a background model file from a user-supplied, fasta-formatted nucleotide sequence file (**Fig. 4a, input #10**; **Supp. Fig. 3c-10**; also see *input #9 / B.3.c.ix*). We used the full human genome sequence (GRCh38.p13 Primary Assembly) as the fasta reference file supplied to FASTA-GET-MARKOV for background model generation (RefSeq Accession ID: GCF_000001405.39; filename:
GCF_000001405.39_GRCh38.p13_genomic.fna;
https://www.ncbi.nlm.nih.gov/assembly/GCF_000001405.39).

B.3.c.xi. Identity of a transcription factor (TF) of interest, to focus specialized analysis on samples and ranked alleles in which TFBS for this TF are predicted

to have been lost. Users can specify an individual TF (optional) for which TFBS are examined in ranked alleles, specifically to interpret whether loss of a TFBS for the specified TF may positionally coincide with 1) gain of a distinct TFBS for the same TF (a scenario we term 'regain': the altered nucleotide sequence attributable to local mismatch and/or indel leads to loss of a TFBS for the TF, but nevertheless a new match to the TF motif as identified by FIMO is identified at the altered position), or 2) gain of a TFBS for a different TF entirely (a scenario we term 'gain'). These interpretations occur in the output file *collated_motifs.xlsx*. Users supply a unique name identifier of the TF of interest that unambiguously matches the name of the TF as it occurs in the MEME format FIMO database supplied in *B.3.c.viii*. "NR3C1" is used in our example to designate the motif for GR: *MA0113.3 NR3C1*. Note that the standardized Entrez gene name must be supplied—not the stable ID (for instance, "MA0113") or the stable ID with version number (for instance, MA0113.3"). Also note that whereas "NR3C1" unambiguously identifies GR, using a truncated name such as "NR3C" would include both GR (*MA0113.3 NR3C1*) and MR (*MA0727.1 NR3C2*) in the analyses.

B.3.d. CollatedMotifs.py—core operation comments

B.3.d.i. Alignment_database and markov_background.txt—Unlike Genotypes.py, CollatedMotifs.py generates the BLASTN alignment database inline, generating a database derived solely from the sequences provided in the reference fasta file (*input #3 / B.3.c.iii*), with six file names prefixed by a custom string (*input #6 / B.3.c.vi*) (*alignment_database,* see **Supp. Fig. 10**). A background Markov file (*markov_background.txt*) is generated from user-defined sequences (*input #10 / B.3.c.x*), to be provided to FIMO during TFBS match operations. MAKEBLASTDB and FASTA-GET-MARKOV are invoked using the Python System Command function.

B.3.d.ii. Fasta.fa—Following entry of user input (*B.3.c. CollatedMotifs.py—user inputs*), the script proceeds to operations that overlap with the fastq→fasta steps in Genotypes.py, linking frequency metrics to ranked sequences in *fasta.fa* (**Fig. 3a-c**, **Fig. 4b-c**). Read 1 (R1) and read 2 (R2) sequences for each sample are merged based on common cluster ID in R1 and R2 fastq files (**Fig. 4b**). Merged

(R1+R2) sequences are channeled into a temporary Python list and filtered for the five most abundant reads, then populated to *fasta.fa* with fasta defline linking sample ID and frequency metrics to sequences (see *B.2.d. Genotypes.py—core operation comments* for further detail). If more than five ranked read ('allele') types are desired, users can readily customize the number of ranked reads output by CollatedMotifs.py by editing the integer argument provided to the function "counter.most_common(5)" from '5' to the desired value (such as '10': counter.most_common(10)"). This function occurs at lines 947 & 951 in CollatedMotifs.py (v1.0).

B.3.d.iii. Blastn_alignments.txt—CollatedMotifs.py passes *fasta.fa* to BLASTN, which generates alignments to sequence content in *alignment_database* (**Fig. 4c**). Alignments in *blastn_alignments.txt* are filtered, such that ranked alleles with 'no hits' or 'multiple hits' are excluded from further analysis, ultimately yielding a dictionary that contains unique sample IDs (keys) linked to tuples (values) comprising the suite of sample ID-derived sequence(s) that aligned to unique loci in *alignment_database*. Data regarding the samples and read types that were filtered out ('no hits' or 'multiple hits') are provided in *population_summary.txt*. As in Genotypes.py, CollatedMotifs.py allows hsp's identified by BLASTN to be reconstructed as hypothesized alleles, if the hsp's meet qualifying criteria (see *B.2.c.vi* for qualifying criteria); rather than requiring BLASTDBCMD to recover the reference sequence span between hsp's to facilitate reconstruction of allele alignment to the reference, however, CollatedMotifs.py refers back to the user-supplied reference fasta file to recover the sequence span between hsp's.

B.3.d.iv. FIMO outputs—CollatedMotifs.py draws on one (*fimo.tsv*) of five FIMO output files that present TFBS identification outputs in distinct formats. Its contents are read into reference- or allele- respective dictionaries, *dict_ref_TFBS* and *dict_allele_TFBS*. A compilation dictionary, *dict_allele_TFBS_synopsis*, ultimately assembles each sample ID (key) linked to a dictionary (value) containing ranked alleles that each point to further subdictionaries: *i)* 'TFs' summarizes the transcription factors with TFBS identified for each allele, *ii)* 'gained' and *iii)* 'lost' list TFBS that are novel or absent in the allele relative to 'all_sites' in *dict_ref_TFBS_synopsis* for the corresponding reference sequence.

For each sample ID in *dict_allele_TFBS_synopsis, collated_TFBS.txt* reports a visual mapping of *i)* TFBSs new to each allele above the alignment (retrieved from *alignmentsoutput_dict2*), and *ii)* TFBSs lost from each allele below the alignment (*e.g.,* 'new TFBS' and 'lost TFBS') (**Fig. 4d**, **Supp. Table 6**, **Supp. Fig. 10**).

B.3.d.v. Collated_motifs.xlsx—In addition to the visual mapping of TFBSs gained and lost for each allele (relative to reference) in *collated_TFBS.txt*, CollatedMotifs.py documents all FIMO-identified TFBSs and interpretations of lost-regained & lost-gained TFBS pairs for every ranked allele across all samples in *collated_motifs.xlsx*, an Excel file with 8 searchable, sortable worksheets (**Fig. 4d**, **Supp. Table 6**, **Supp. Fig. 10** (*see Supp. Fig. 10c in particular for details*).

B.3.d.vi. Script_metrics.txt—Like Genotypes.py, CollatedMotifs.py logs script operation parameters in *script_metrics.txt*, specifically preserving *i)* operating system information, *ii)* user-defined variables, *iii)* fastq file properties, *iv)* position frequency matrix file properties (TF metadata), *v)* file output information, and *vi)* script operation times (*e.g.*, start time, MAKEBLASTDB and FASTA-GET-MARKOV processing time, fasta processing time, alignments processing time, FIMO processing time, etc.) (**Supp. Fig. 10**).

Supplementary Tables

Supplementary Table 1. Public sources for software dependencies.

Software	Source
BLAST+ suite (NCBI) (2)	ftp://ftp.ncbi.nlm.nih.gov/blast/executables/blast+/LATEST/
MEME suite (9)	http://meme-suite.org/doc/download.html
Python 3.0 or higher	https://www.python.org/downloads/
Jupyter Notebook	https://jupyter.org/
[alternatively, Anaconda with Jupyter Notebook]	https://www.anaconda.com/products/individual
Python PrettyTable library	https://pypi.org/project/PrettyTable/ *or from GitHub,* https://github.com/jazzband/prettytable
Python NumPy, SciPy libraries	https://www.scipy.org/scipylib/download.html https://pypi.org/project/numpy/
Python fpdf, PyPDF2 libraries	https://pypi.org/project/fpdf/ https://pypi.org/project/PyPDF2/
Oracle VM VirtualBox *(if using virtual machine)*	https://www.virtualbox.org/wiki/Downloads
alleles_and_altered_motifs.ova *(virtual machine)*	https://doi.org/10.5281/zenodo.3406861

Table Key:

External dependencies
Python & Jupyter Notebook
Python dependencies
Virtualization

Supplementary Table 2. SampleSheet.py inputs.

User input	Purpose	Example
Illumina Dual Indexed Sequencing Workflow		
Workflow<br>A<br>*vs.*<br>B	Specify whether i5 and i7 indices are reported during paired-end (PE) sequencing as 'forward' or 'reverse complement' sequences	A
Absolute path to Sample Sheet file name		
Output directory & Sample Sheet file name	Define directory in which Sample Sheet file (with designated name) will be created	/Users/name/IlluminaNGS/SampleSheet.csv (MacOS/Linux)<br>or<br>C:\Users\name\IlluminaNGS\SampleSheet.csv (Windows)
Sample Sheet sections		
[Header]:<br>Investigator Name, Project Name	Customize investigator name and project name to appear in [Header] section	Dorothy Gale, Cas9-edited clones
[Reads]:<br>SE *vs.* PE, and # of cycles for R1/R2	Specify cycle number for R1 (SE) or R1 and R2 (PE)	PE, 151, 151
[Data]:<br>Sample:barcode relationships	Specify relationship between sample name common to wells of a 96-well plate, and i7/i5 indices	*CLI:*<br>DG-1, 1-96, 1<br>DG-2, 1-96, 9<br>DG-3, 1-50, 78<br><br>*Jupyter Notebook:*<br>DG-1, 1-96, 1; DG-2, 1-96, 9; DG-3, 1-50, 78

Supplementary Table 3. Genotypes.py inputs.

User input	Purpose	Example
Absolute paths		
Output directory	Directory in which output files will be created	/Users/name/Documents/Genotypes (MacOS/Linux) or C:\Users\name\Documents\Genotypes (Windows)
Fastq (input) directory	Directory that will supply fastq file(s) corresponding to sample IDs	/Users/name/Documents/data (MacOS/Linux) or C:\Users\name\Documents\data (Windows)
BLASTN installation (executable)	NCBI software that scans a nucleotide sequence database for 'hit(s)' that align with query sequence(s), returning hit coordinates and relative alignment between hit and query	/usr/local/bin/blast/bin/blastn (MacOS/Linux) or C:\windows\system32\blast\bin\blastn (Windows)
BLASTN database (directory)	Directory created from MAKEBLASTDB operation on a single fasta file containing reference sequence entries, for alignment of sequenced reads	/Users/name/Documents/blastn_database (MacOS/Linux) or C:\Users\name\Documents\blastn_database (Windows)
BLASTDBCMD installation (executable)	NCBI software that retrieves sequence from a nucleotide sequence database when presented with appropriate accession code (available within database) and coordinates	usr/local/bin/blast/bin/blastdbcmd (MacOS/Linux) or C:\windows\system32\blast\bin\blastdbcmd (Windows)

Database prefix		
Short text string provided as prefix common to alignment database files	Prefix common to all alignment database files (.nhr, .nin, .nog, .nsd, .nsi, .nsg)	GRCh38
Short DNA sequences (optional)		
Guide RNA sequence(s)	Nucleotide sequence for guide RNA(s) used in Cas9 editing effort (in DNA format, 5'→3', excluding PAM)	ATCCAGTTCTCCAGTCTCCC, GCGAGCTCGTGTCTGTGACG
Query DNA sequence(s)	Nucleotide sequence for DNA subsequence(s) to test for presence/absence in inferred alleles	TACTCAATATCGATC, CGGGAGCCCGAG

Supplementary Table 4. File outputs of Genotypes.py.

File name	Purpose	Notes
fasta.fa	*1)* Populates with fasta-formatted sequence entries for most abundant reads (top 10, merged read1+read2) belonging to each sample ID (fastq file) among the input fastq files; each sequence defline records sample ID (*e.g.*, source plate & well number) and sequence frequency metrics; *2)* input to BLASTN for sequence alignments	
blastn_alignments.txt	Output of BLASTN operation on fasta.fa entries relative to BLASTN sequence database	This can be a large (multi-GB) file, accounting for hard drive free space needs and/or requiring operation on a server
allele_definitions.txt	Output of script operation on blastn_alignments.txt (allele definitions and inferred genotypes)	Samples are returned in order of Sample ID name
allele_evidence.pdf	Plots of allele abundance, for (up to) 10 'ranked' alleles; visual representation of frequency evidence used by Genotypes.py as basis of sample genotype	This file is optional. Creation of this file is time-intensive (*e.g.*, 2-3 min/sample, depending on RAM) and the output file can be large (multi-MB or GB); user can therefore optionally include *vs.* bypass code block that generates this file (prompt occurs during script operation to make this decision)
genotypes.txt	Output of script operation on blastn_alignments.txt (allele definitions and inferred genotypes), sorted based on nature of inferred genotype	Samples are returned in order of inferred genotype (specifically, in the following order: *1)* homozygous deletions, *2)* homozygous insertions, *3)* homozygous indels, *4)* homozygous substitutions, *5)* biallelic deletions, *6)* biallelic insertions, *7)* biallelic indels, *8)* biallelic substitutions, *9)* biallelic 'other', *10)* heterozygous

		deletions, *11)* heterozygous insertions, *12)* heterozygous indels, *13)* heterozygous substitutions, *14)* multizygous, *15)* homozygous wild-type, *16)* genotype unclear);<br>here, *wild-type* refers to the sequence as it occurs in the user-provided alignment database
allele_definitions.csv	Comma-separated file cataloguing data acquired for each of (up to) the top 10 most abundant reads/sample (*Ranked Alleles*: rank, underlying individual read abundance, alignment to reference sequence, inferred allele type, inferred genotype for corresponding sample source)	Can be opened in Excel or imported as a Python dataframe object for further user-customized evaluation/processing; deprecated Ranked Alleles are not logged in this file (but are logged in population_summary.txt) (see *B.2.d.i 'Notes'* for definition of deprecated Ranked Alleles, and *population_summary.txt*, next row in this table)
population_summary.txt	Documents *1)* # of samples processed for genotype inference, % 'called' *vs.* 'uncalled' for inference; *2)* representation of allele and genotype 'categories' population-wide; *3)* deprecated ranked alleles: frequency-ranked reads with 'no hits' in the reference database, 'multiple hits' in the reference database, or (if associated with >1 high-scoring alignment pair), "overlapping hsp's" or hsp's spanning >1 kb (deprecated=excluded from genotype inference analysis)	Aside from providing population summaries for genotypes, this file provides data for further investigation of the amplicons recovered by PCR; sample names/alleles flagged as having 'no hits' or 'multiple hits' among read(s) can be investigated in blastn_alignments.txt and/or fasta.fa to evaluate explanations for multiple alignments and/or to pursue further investigation of the read sequence(s) in question (*e.g.*, conventional web-based nucleotide BLAST against a wide number of genomes)
script_metrics.txt	Documents inputs to script operation (fastq files) and records performance parameters (operation processing times)	

Supplementary Table 5. CollatedMotifs.py inputs. Note that in Windows OS, MEME suite (9) programs (FIMO and FASTA-GET-MARKOV) require virtualization and CollatedMotifs.py must be run from within a hypervisor (*e.g.*, Oracle VirtualBox; Open Virtualization Format file available at https://zenodo.org/record/3406861, DOI: 10.5281/zenodo.3406861).

**User input**	**Purpose**	**Example**
Absolute paths		
Output directory	Directory in which output files will be created	/Users/name/Documents/CollatedMotifs (MacOS, Linux)<br>or<br>C:\Users\name\Documents\CollatedMotifs (Windows)
Fastq (input) directory	Directory that will supply fastq file(s) corresponding to sample IDs	/Users/name/Documents/data (MacOS, Linux)<br>or<br>C:\Users\name\Documents\data (Windows)
Fasta reference file	Fasta file containing reference sequence(s) as source of TFBS for comparison in alleles; *CRITICAL:* reference sequences must be named (defline) such that defline name can be found in sample name(s) for TFBS comparison (see *B.3.c.iii*); *i.e.*, string search of defline finds match in sample name(s) slated for comparison to the indicated reference sequence	/Users/name/Documents/fasta_ref.fa (MacOS, Linux)<br>or<br>C:\Users\name\Documents\fasta_ref.fa (Windows)
BLASTN installation (executable)	NCBI software that scans a nucleotide sequence database for 'hit(s)' that align with query	/usr/local/bin/blast/bin/blastn (MacOS, Linux)<br>or<br>C:\windows\system32\blast\bin\blastn (Windows)

	sequence(s), returning hit coordinates and relative alignment between hit and query	
MAKEBLASTDB installation (executable)	NCBI software that creates an alignment database from a fasta file containing reference sequence(s)	/usr/local/bin/blast/bin/makeblastdb (MacOS, Linux)<br>or<br>C:\windows\system32\blast\bin\makeblastdb (Windows)
FIMO installation (executable)	MEME suite software that identifies matches to TFBS motifs in sequences provided in fasta format	/usr/local/bin/Meme/bin/fimo<br>(MacOS, Linux)<br>or<br>C:\windows\system32\Meme\bin\fimo<br>(Windows)
FIMO position frequency matrix file (MEME format)	File used by FIMO as a basis of TFBS match searches	/usr/local/bin/Meme/motif_databases/JASPAR/JASPAR_CORE_2016_vertebrates.meme<br>(MacOS, Linux)<br>or<br>C:\windows\system32\Meme\motif_databases\JASPAR\JASPAR_CORE_2016_vertebrates.meme<br>(Windows)
FASTA-GET-MARKOV installation (executable)	MEME suite software that generates a Markov background statistical model for TFBS motif matches called by FIMO	/usr/local/bin/Meme/src/fastagetmarkov<br>(MacOS, Linux)<br>or<br>C:\windows\system32\Meme\src\fastagetmarkov<br>(Windows)
Fasta file as background for FASTA-GET-MARKOV	File containing sequence(s) used by FASTA-GET-MARKOV to generate a background statistical model for TFBS match searches	/Users/name/GRCh38.fa<br>(MacOS, Linux)<br>or<br>C:\Users\name\GRCh38.fa<br>(Windows)

Database prefix		
Short text string provided as prefix common to alignment database files	Prefix common to all alignment database files (.nhr, .nin, .nog, .nsd, .nsi, .nsg)	GRCh38
TF of interest		
Short text string provided as transcription factor (TF) for focused analysis of loss among sample ranked alleles	Text string must be standardized Entrez gene name for TF of interest	NR3C1

Supplementary Table 6. File outputs of CollatedMotifs.py. The script generates 6 separate files, plus three directories (generated by MAKEBLASTDB and FIMO).

**File name**	**Purpose**	**Notes**
Files		
fasta.fa	*1)* Populates with fasta-formatted sequence entries for most abundant reads (top 5, merged read1+read2) belonging to each sample ID (fastq file) among the input fastq files; each sequence defline records sample ID (*e.g.*, source plate & well number) and sequence frequency metrics; *2)* input to BLASTN for sequence alignments	
blastn_alignments.txt	Output of BLASTN operation on fasta.fa entries relative to BLASTN sequence database	This can be a large (multi-GB) file, accounting for hard drive free space needs and/or requiring operation on a server
markov_background.txt	Output of FASTA-GET-MARKOV on user-provided fasta file of sequences; background model for expected nucleotide frequencies	
collated_TFBS.txt	Output of script operation on blastn_alignments.txt (allele definitions) and fimo.tsv (tables of TFBS identified by FIMO for alleles and reference sequence(s)). Presents a collation of 'lost' and 'new' TFBS for alleles relative to reference sequence, in context of sequence alignment	Primary data file
collated _TFBS.xlsx	Multi-worksheet Excel file cataloguing TFBS data acquired for each of (up to) the top 5 most abundant reads/sample (including annotation of TFBSs as 'lost' or 'gained' in allele, relative to TFBSs identified in reference sequence)	Can be opened in Excel, or individual worksheets can be imported as Python dataframe object(s) for further user-customized evaluation/processing (*see Supp. Fig. 10 for details of worksheet names and contents*)
script_metrics.txt	Documents inputs to script operation (fastq files, position frequency file, etc.) and records	

	performance parameters (operation processing times)	
Directories		
alignment_database	BLASTN alignment database composed of six files (.nhr, .nin, .nog, .nsd, .nsi, .nsq), generated by MAKEBLASTDB from user-supplied reference sequence(s) in fasta format	Files in this database share a user-supplied prefix provided as a short text string during user input
fimo_out	Directory of six files created by FIMO operations on fasta.fa and markov_background.txt (cisml.xml, fimo.gff, fimo.html, fimo.tsv, fimo.xml)	Fimo.tsv in fimo_out contains sequence matches to TFBS motifs (with p-value) for each allele; its contents are the basis for the collation of TFBS loss/gain evaluated by CollatedMotifs.py, relative to the cognate data for reference sequence(s) in fimo_out_ref
fimo_out_ref	Directory of six files created by FIMO operations on user-supplied fasta file containing reference sequence(s), and markov_background.txt (cisml.xml, fimo.gff, fimo.html, fimo.tsv, fimo.xml)	

Supplementary Figures

Supplementary Figure S1. 96x96 array of Illumina® sequencing platform-compatible i7 and i5 barcode primers.

(a) 192 primers (ninety-six “i7/index 1” and ninety-six “i5/index 2”) are easily ordered in 96-well plate format from commercial oligonucleotide suppliers (*e.g.*, IDT, Coralville, IA), and can be used to label amplicons in up to 9,216 unique combinations for dual-indexed (paired-end) sequencing;

(b) up to 9,216 samples prepared for 96x96 dual barcoding can be arrayed in up to ninety-six 96-well plates or up to twenty-four 384-well plates (for example, see **Supp. Fig.** 7);

(c) i7 and i5 barcode sequences identified according to position in a 96-well plate when ordered from the .csv template provided (**Supp. Excel file**); 8-bp barcode sequence indicated 5’→3’ as it appears in the primer (i7 or i5), or 5’→3’ as it appears in the reverse complement (rev comp) frequently recovered as the index sequence in an Illumina® sequencing workflow (1).

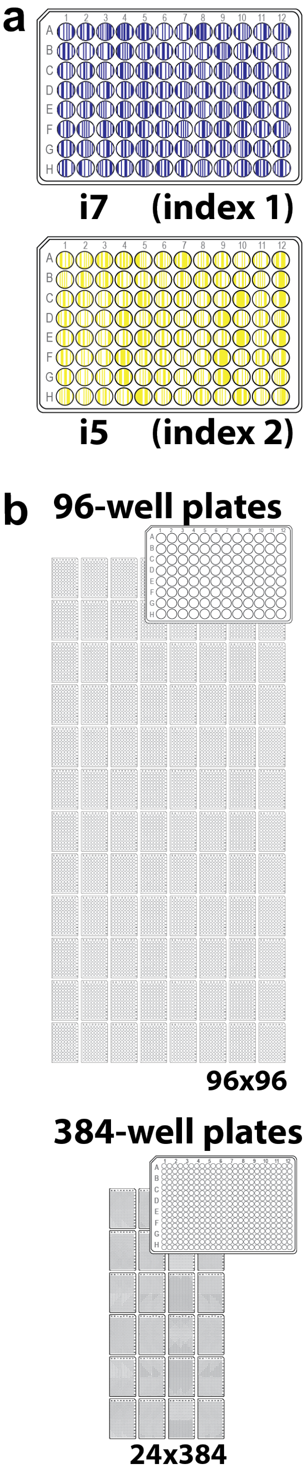

c

i7				i5			
well	name	i7	rev. comp.	well	name	i5	rev. comp.
A1	read2_bc_1_TGACGTAC	TGACGTAC	GTACGTCA	A1	read1_bc_1_GAGGTAGT	GAGGTAGT	CTCCATCA
A2	read2_bc_2_TAACTGCA	TAACTGCA	TGCAGTTA	A2	read1_bc_2_GCTTAACT	GCTTAACT	CGAATTGA
A3	read2_bc_3_TCCACAGT	TCCACAGT	ACTGTGGA	A3	read1_bc_3_GCAATTCT	GCAATTCT	CGTTAAGA
A4	read2_bc_4_TCTTAACC	TCTTAACC	GGTTAAGA	A4	read1_bc_4_TCCTCACT	TCCTCACT	AGGAGTGA
A5	read2_bc_5_TAGTGTGA	TAGTGTGA	TCACACTA	A5	read1_bc_5_AGTTAGCT	AGTTAGCT	TCAATCGA
A6	read2_bc_6_TACCTCTA	TACCTCTA	TAGAGGTA	A6	read1_bc_6_TCATGGCT	TCATGGCT	AGTACCGA
A7	read2_bc_7_TATGTCGC	TATGTCGC	GCGACATA	A7	read1_bc_7_AGCCTAAT	AGCCTAAT	TCGGATTA
A8	read2_bc_8_TGCATGTA	TGCATGTA	TACATGCA	A8	read1_bc_8_AACACTCT	AACACTCT	TTGTGAGA
A9	read2_bc_9_TCATCATC	TCATCATC	GATGATGA	A9	read1_bc_9_AGAGCTCT	AGAGCTCT	TCTCGAGA
A10	read2_bc_10_TGCACACA	TGCACACA	TGTGTGCA	A10	read1_bc_10_GGCCATAT	GGCCATAT	CCGGTATA
A11	read2_bc_11_TGTAGCGA	TGTAGCGA	TCGCTACA	A11	read1_bc_11_TAGAACGT	TAGAACGT	ATCTTGCA
A12	read2_bc_12_TCTAGCTT	TCTAGCTT	AAGCTAGA	A12	read1_bc_12_GGTTGAAT	GGTTGAAT	CCAACTTA
B1	read2_bc_13_TGGTCCTA	TGGTCCTA	TAGGACCA	B1	read1_bc_13_GATCCTAT	GATCCTAT	CTAGGATA
B2	read2_bc_14_TCCAACGA	TCCAACGA	TCGTTGGA	B2	read1_bc_14_TGGTCTCT	TGGTCTCT	ACCAGAGA
B3	read2_bc_15_TAGGACAC	TAGGACAC	GTGTCCTA	B3	read1_bc_15_TCCAGTCT	TCCAGTCT	AGGTCAGA
B4	read2_bc_16_TATACGGA	TATACGGA	TCCGTATA	B4	read1_bc_16_TAGCGTAT	TAGCGTAT	ATCGCATA
B5	read2_bc_17_TAAGCGTA	TAAGCGTA	TACGCTTA	B5	read1_bc_17_GCGATCAT	GCGATCAT	CGCTAGTA
B6	read2_bc_18_TCGTTGTA	TCGTTGTA	TACAACGA	B6	read1_bc_18_AGCTACAT	AGCTACAT	TCGATGTA
B7	read2_bc_19_TCTCCTTC	TCTCCTTC	GAAGGAGA	B7	read1_bc_19_ACTCATCT	ACTCATCT	TGAGTAGA
B8	read2_bc_20_TGATGCTT	TGATGCTT	AAGCATCA	B8	read1_bc_20_TTGCGAGT	TTGCGAGT	AACGCTCA
B9	read2_bc_21_TGATCTCA	TGATCTCA	TGAGATCA	B9	read1_bc_21_AAGCGACT	AAGCGACT	TTCGCTGA
B10	read2_bc_22_TGTCTAGA	TGTCTAGA	TCTAGACA	B10	read1_bc_22_TGCACGAT	TGCACGAT	ACGTGCTA
B11	read2_bc_23_TATGAGCA	TATGAGCA	TGCTCATA	B11	read1_bc_23_TATGCGGT	TATGCGGT	ATACGCCA
B12	read2_bc_24_TGGAGTTA	TGGAGTTA	TAACTCCA	B12	read1_bc_24_TCGTCGAT	TCGTCGAT	AGCAGCTA
C1	read2_bc_25_TGTGTGTC	TGTGTGTC	GACACACA	C1	read1_bc_25_ACACTTGT	ACACTTGT	TGTGAACA
C2	read2_bc_26_TGCTACAA	TGCTACAA	TTGTAGCA	C2	read1_bc_26_TACGGCAT	TACGGCAT	ATGCCGTA
C3	read2_bc_27_TCGTAGAC	TCGTAGAC	GTCTACGA	C3	read1_bc_27_TCTCGTGT	TCTCGTGT	AGAGCACA
C4	read2_bc_28_TGTAATGC	TGTAATGC	GCATTACA	C4	read1_bc_28_GCAGATGT	GCAGATGT	CGTCTACA
C5	read2_bc_29_TATGGAAC	TATGGAAC	GTTCCATA	C5	read1_bc_29_TCCGTTGT	TCCGTTGT	AGGCAACA
C6	read2_bc_30_TGGTATTC	TGGTATTC	GAATACCA	C6	read1_bc_30_TTCGCAGT	TTCGCAGT	AAGCGTCA
C7	read2_bc_31_TCAAGTGA	TCAAGTGA	TCACTTGA	C7	read1_bc_31_GGAATAGT	GGAATAGT	CCTTATCA
C8	read2_bc_32_TCTTGTGT	TCTTGTGT	ACACAAGA	C8	read1_bc_32_GCAACAAT	GCAACAAT	CGTTGTTA
C9	read2_bc_33_TCTGTAGT	TCTGTAGT	ACTACAGA	C9	read1_bc_33_ACTGCGAT	ACTGCGAT	TGACGCTA
C10	read2_bc_34_TCCTGAGA	TCCTGAGA	TCTCAGGA	C10	read1_bc_34_TGAGTACT	TGAGTACT	ACTCATGA
C11	read2_bc_35_TGCACGAT	TGCACGAT	ATCGTGCA	C11	read1_bc_35_ACACGAAT	ACACGAAT	TGTGCTTA
C12	read2_bc_36_TACGTGGA	TACGTGGA	TCCACGTA	C12	read1_bc_36_GTGGCAAT	GTGGCAAT	CACCGTTA
D1	read2_bc_37_TCATGGAT	TCATGGAT	ATCCATGA	D1	read1_bc_37_GTCATCCT	GTCATCCT	CAGTAGGA
D2	read2_bc_38_TCTGCGAA	TCTGCGAA	TTCGCAGA	D2	read1_bc_38_ACCGTACT	ACCGTACT	TGGCATGA
D3	read2_bc_39_TGAGACGA	TGAGACGA	TCGTCTCA	D3	read1_bc_39_GTACTACT	GTACTACT	CATGATGA
D4	read2_bc_40_TCGTAAGT	TCGTAAGT	ACTTACGA	D4	read1_bc_40_TAGGTCCT	TAGGTCCT	ATCCAGGA
D5	read2_bc_41_TAATCCGA	TAATCCGA	TCGGATTA	D5	read1_bc_41_GTTACTGT	GTTACTGT	CAATGACA
D6	read2_bc_42_TAGACCAA	TAGACCAA	TTGGTCTA	D6	read1_bc_42_GCCTTAGT	GCCTTAGT	CGGAATCA
D7	read2_bc_43_TCCGCTAT	TCCGCTAT	ATAGCGGA	D7	read1_bc_43_AACCATGT	AACCATGT	TTGGTACA
D8	read2_bc_44_TGGTTACA	TGGTTACA	TGTAACCA	D8	read1_bc_44_GATGACAT	GATGACAT	CTACTGTA
D9	read2_bc_45_TAGCCTGT	TAGCCTGT	ACAGGCTA	D9	read1_bc_45_AGAAGACT	AGAAGACT	TCTTCTGA
D10	read2_bc_46_TGCGACTT	TGCGACTT	AAGTCGCA	D10	read1_bc_46_TCAAGCAT	TCAAGCAT	AGTTCGTA
D11	read2_bc_47_TCGAACTC	TCGAACTC	GAGTTCGA	D11	read1_bc_47_TGGATGCT	TGGATGCT	ACCTACGA
D12	read2_bc_48_TGAAGAGT	TGAAGAGT	ACTCTTCA	D12	read1_bc_48_ACTTGCCT	ACTTGCCT	TGAACGGA
E1	read2_bc_49_TAGGTCTT	TAGGTCTT	AAGACCTA	E1	read1_bc_49_GTTGTGAT	GTTGTGAT	CAACACTA
E2	read2_bc_50_TGGATGGT	TGGATGGT	ACCATCCA	E2	read1_bc_50_TATCTCGT	TATCTCGT	ATAGAGCA
E3	read2_bc_51_TACCGTTC	TACCGTTC	GAACGGTA	E3	read1_bc_51_TGTAAGGT	TGTAAGGT	ACATTCCA
E4	read2_bc_52_TCTCACTA	TCTCACTA	TAGTGAGA	E4	read1_bc_52_ACGTTGGT	ACGTTGGT	TGCAACCA
E5	read2_bc_53_TGTTGGAC	TGTTGGAC	GTCCAACA	E5	read1_bc_53_AGGAGGAT	AGGAGGAT	TCCTCCTA
E6	read2_bc_54_TCGCCATA	TCGCCATA	TATGGCGA	E6	read1_bc_54_GAGCAGAT	GAGCAGAT	CTCGTCTA
E7	read2_bc_55_TGATAAGC	TGATAAGC	GCTTATCA	E7	read1_bc_55_TCTCTACT	TCTCTACT	AGAGATGA
E8	read2_bc_56_TCAGTCAC	TCAGTCAC	GTGACTGA	E8	read1_bc_56_GGATTCAT	GGATTCAT	CCTAAGTA
E9	read2_bc_57_TATCACGT	TATCACGT	ACGTGATA	E9	read1_bc_57_GACCTTCT	GACCTTCT	CTGGAAGA
E10	read2_bc_58_TAAGAACC	TAAGAACC	GGTTCTTA	E10	read1_bc_58_ACGGTTAT	ACGGTTAT	TGCCAATA
E11	read2_bc_59_TGCTGATC	TGCTGATC	GATCAGCA	E11	read1_bc_59_AGATGCGT	AGATGCGT	TCTACGCA
E12	read2_bc_60_TATTGTCC	TATTGTCC	GGACAATA	E12	read1_bc_60_TGACACCT	TGACACCT	ACTGTGGA
F1	read2_bc_61_TCAGAGTT	TCAGAGTT	AACTCTGA	F1	read1_bc_61_ACTAACGT	ACTAACGT	TGATTGCA
F2	read2_bc_62_TAAGGTCA	TAAGGTCA	TGACCTTA	F2	read1_bc_62_GAATGCCT	GAATGCCT	CTTACGGA
F3	read2_bc_63_TCACTTCC	TCACTTCC	GGAAGTGA	F3	read1_bc_63_GTGTACGT	GTGTACGT	CACATGCA
F4	read2_bc_64_TCCTTCTT	TCCTTCTT	AAGAAGGA	F4	read1_bc_64_AAGTCAGT	AAGTCAGT	TTCAGTCA
F5	read2_bc_65_TAGATTGC	TAGATTGC	GCAATCTA	F5	read1_bc_65_TTCCTGGT	TTCCTGGT	AAGGACCA
F6	read2_bc_66_TGACTCGT	TGACTCGT	ACGAGTCA	F6	read1_bc_66_ACCACCAT	ACCACCAT	TGGTGGTA
F7	read2_bc_67_TGGCATAA	TGGCATAA	TTATGCCA	F7	read1_bc_67_GAGTCCAT	GAGTCCAT	CTCAGGTA
F8	read2_bc_68_TCTATGGC	TCTATGGC	GCCATAGA	F8	read1_bc_68_AGCTCTGT	AGCTCTGT	TCGAGACA
F9	read2_bc_69_TCCGATTA	TCCGATTA	TAATCGGA	F9	read1_bc_69_GGTCTTGT	GGTCTTGT	CCAGAACA
F10	read2_bc_70_TAACGAGA	TAACGAGA	TCTCGTTA	F10	read1_bc_70_AATGTGCT	AATGTGCT	TTACACGA
F11	read2_bc_71_TACAGACC	TACAGACC	GGTCTGTA	F11	read1_bc_71_GTCTGGAT	GTCTGGAT	CAGACCTA
F12	read2_bc_72_TACCGGAT	TACCGGAT	ATCCGGTA	F12	read1_bc_72_GCGTATAT	GCGTATAT	CGCATATA
G1	read2_bc_73_TGTCGCAT	TGTCGCAT	ATGCGACA	G1	read1_bc_73_TGACTGAT	TGACTGAT	ACTGACTA
G2	read2_bc_74_TGCGTTCA	TGCGTTCA	TGAACGCA	G2	read1_bc_74_TGTCCAGT	TGTCCAGT	ACAGGTCA
G3	read2_bc_75_TCATACCA	TCATACCA	TGGTATGA	G3	read1_bc_75_TTGTGCCT	TTGTGCCT	AACACGGA
G4	read2_bc_76_TCTGTCCA	TCTGTCCA	TGGACAGA	G4	read1_bc_76_ACAGACCT	ACAGACCT	TGTCTGGA
G5	read2_bc_77_TCTTGCAA	TCTTGCAA	TTGCAAGA	G5	read1_bc_77_GATCAAGT	GATCAAGT	CTAGTTCA
G6	read2_bc_78_TACCTAGT	TACCTAGT	ACTAGGTA	G6	read1_bc_78_GATAGGAT	GATAGGAT	CTATCCTA
G7	read2_bc_79_TATCGGTA	TATCGGTA	TACCGATA	G7	read1_bc_79_GTAGTCGT	GTAGTCGT	CATCAGCA
G8	read2_bc_80_TCACCAAT	TCACCAAT	ATTGGTGA	G8	read1_bc_80_TCACCTAT	TCACCTAT	AGTGGATA
G9	read2_bc_81_TCGAGGAA	TCGAGGAA	TTCCTCGA	G9	read1_bc_81_AGTGGTAT	AGTGGTAT	TCACCATA
G10	read2_bc_82_TACCATGA	TACCATGA	TCATGGTA	G10	read1_bc_82_GCTAGAGT	GCTAGAGT	CGATCTCA
G11	read2_bc_83_TGTGGATA	TGTGGATA	TATCCACA	G11	read1_bc_83_TTAACGCT	TTAACGCT	AATTGCGA
G12	read2_bc_84_TACAAGTC	TACAAGTC	GACTTGTA	G12	read1_bc_84_TCAGGAGT	TCAGGAGT	AGTCCTCA
H1	read2_bc_85_TACTCCAT	TACTCCAT	ATGGAGTA	H1	read1_bc_85_AACTGGCT	AACTGGCT	TTGACCGA
H2	read2_bc_86_TATCTGAC	TATCTGAC	GTCAGATA	H2	read1_bc_86_TACAGAGT	TACAGAGT	ATGTCTCA
H3	read2_bc_87_TAGTGCTC	TAGTGCTC	GAGCACTA	H3	read1_bc_87_TTGCATCT	TTGCATCT	AACGTAGA
H4	read2_bc_88_TGAATGAC	TGAATGAC	GTCATTCA	H4	read1_bc_88_GAACGTGT	GAACGTGT	CTTGCACA
H5	read2_bc_89_TCTTAGGA	TCTTAGGA	TCCTAAGA	H5	read1_bc_89_AAGTACCT	AAGTACCT	TTCATGGA
H6	read2_bc_90_TACGCATC	TACGCATC	GATGCGTA	H6	read1_bc_90_GGTACCAT	GGTACCAT	CCATGGTA
H7	read2_bc_91_TGGAAGCA	TGGAAGCA	TGCTTCCA	H7	read1_bc_91_AATCGCAT	AATCGCAT	TTAGCGTA
H8	read2_bc_92_TCTGACAT	TCTGACAT	ATGTCAGA	H8	read1_bc_92_GGTAATCT	GGTAATCT	CCATTAGA
H9	read2_bc_93_TGAGCAAC	TGAGCAAC	GTTGCTCA	H9	read1_bc_93_GATTCGCT	GATTCGCT	CTAAGCGA
H10	read2_bc_94_TGGATATC	TGGATATC	GATATCCA	H10	read1_bc_94_AGCATCGT	AGCATCGT	TCGTAGCA
H11	read2_bc_95_TCGCTAAC	TCGCTAAC	GTTAGCGA	H11	read1_bc_95_GTTCACCT	GTTCACCT	CAAGTGGA
H12	read2_bc_96_TGTACCTC	TGTACCTC	GAGGTACA	H12	read1_bc_96_AGGCAAGT	AGGCAAGT	TCCGTTCA

Supplementary Figure S2. Overview of customized Illumina® Sample Sheet sections populated by SampleSheet.py.

The Illumina® Sample Sheet is indispensable for accurate demultiplexing of pooled reads to individual, sample-specific fastq files. Four sections of the Illumina® Sample Sheet are customized by user input at the SampleSheet.py interface: *InvestigatorName, ProjectName, [Reads]* (single-end/SE or paired-end/PE; cycle #(s) for read1, read2), and contents of the *[Data]* table. *Date* is auto-generated using system's current calendar date.

Should users wish to customize default key values, Illumina® publications can be consulted for further details and metadata key:value options; default parameters may be readily customized by editing the appropriate field content of the SampleSheet *.csv file output, or contents of the script itself (*e.g.*, Jupyter Notebook section labeled, 'IV. Create Sample Sheet and populate with content').

[Header]
InvestigatorName, ***user-supplied***
ProjectName, ***user-supplied***
Date, ***auto-generated***
Workflow, GenerateFASTQ
Application, FASTQonly
Description, Sequencing
Chemistry, Amplicon

[Reads]
user-supplied

[Settings]
ReverseComplement, 0
Adapter, CTGTCTCTTATACACATCT

[Data]
Sample_ID, Sample_Name, i7_Index_ID, index, i5_Index_ID, index2
user-supplied

Supplementary Figure S3. Command-line and Jupyter Notebook interface (console) views of user prompts

(examples shown are from command-line interface)

(a) SampleSheet.py;
(b) Genotypes.py;
(c) CollatedMotifs.py.

(a) SampleSheet.py

1.

```
--------------------------------------------
Illumina Indexed Sequencing Workflow (A or B)
--------------------------------------------

Illumina Indexed Sequencing for dual-indexed (Paired End) runs uses one of two different Workflows (A or B),
defined by whether (A) both index sequences (i7 and i5) are sequenced by primers that anneal to the 'Read 1' strand,
or (B) i7 index is sequenced by a primer that anneals to the 'Read 1' strand, whereas i5 index is sequenced
by a primer that anneals to the 'Read 2' strand.

Awareness of the different Workflows for sequencing the indices is critical, because Workflow determines whether
the 5'->3' index sequence (i5 or i7) is returned as a 'forward' sequence that reads just like the index sequence
as it occurs in indexing primers during library preparation, or whether the 5'->3' index sequence is returned as a
'reverse complement' relative to the index sequence as it occurs in indexing primers during library preparation.
Fundamentally, Workflow determines the 5'->3' nucleotide sequences entered for i7 and i5 indices in an Illumina
Sample Sheet, essential for faithful demultiplexing of samples.

As of December 2020, Illumina sequencing instruments use the two dual-indexed Workflows as follows:

  Workflow A: MiSeq, NovaSeq 6000, HiSeq 2500, HiSeq 2000
  -----------------------------------------------------
  --> i7 index sequence is recovered as 'reverse complement' relative to i7 sequence as it occurs in primers used
  for library construction.
  --> i5 index sequence is recovered as it occurs in primers used for library construction.

  Workflow B: iSeq100, MiniSeq, NextSeq, HiSeqX, HiSeq 4000, HiSeq 3000
  -------------------------------------------------------------------
  --> i7 index sequence is recovered as 'reverse complement' relative to i7 sequence as it occurs in primers used
  for library construction.
  --> i5 index sequence is recovered as 'reverse complement' relative to i5 sequence as it occurs in primers used
  for library construction.

Please confirm the Workflow appropriate for your sequencing application.

Note, if your application is strictly single-indexed (Single End run using only i7 indices), choose Workflow 'A'.

Enter 'A' or 'B' to specify the Workflow, and therefore the index sequence orientations, appropriate for your Sample
Sheet:
```

2.

```
--------------------------------------------------------------------------
Sample Sheet file name and location (absolute path to future .csv filename)
--------------------------------------------------------------------------

***** Enter the name of the .csv file you'd like to create as your Sample Sheet, with an absolute path to its
location.*****

The .csv file should not exist yet -- it will be created as an output of this script.

To create the .csv file in a directory where you can find it, enter an *absolute path*
to where you would like this file to be created, using only forward slashes ('/') to indicate directory separations.

Example: if your target file name is 'SampleSheet.csv', and you'd like to create that file
in a directory that is accessed with an absolute path of '/Users/myname/Illumina/SampleSheet.csv' (Mac) or
'C:\Users\myname\Illumina\SampleSheet.csv' (PC), enter '/Users/myname/Illumina/SampleSheet.csv' (Mac) or
'C:/Users/myname/Illumina/SampleSheet.csv' (PC) at the command line prompt. Replace 'myname, etc.' with the
appropriate intervening directory identifiers. Do *not* flank your entry with quotation marks (') at the command-line.

Alternatively, simply enter a target file name (e.g., 'SampleSheet.csv') and run this script from
within a directory where you'd like to output this file.

-----> File name and path:
```

3.

```
----------------------------------------------------------------
Sample Sheet inputs: [Header], [Reads], and [Data] specifications
----------------------------------------------------------------

...................................................................
***** [Header] details: specify InvestigatorName & ProjectName *****

To specify InvestigatorName & ProjectName, enter text for each directly at the command line,
separated by a comma ('InvestigatorName, ProjectName').

When both text entries are entered, press ‘Enter’ again to proceed in the script.
To skip text entries for these fields, simply press ‘Enter’ until the next prompt appears (if you skip
entry now, 'NA' will be entered in these fields in the output file).

Example: if your InvestigatorName is 'Dorothy Gale' and ProjectName is 'Sequences', enter
'Dorothy Gale, Sequences'.

-----> [Header] details. InvestigatorName & ProjectName:
```

4.

```
...........................................................................................................
***** [Reads] details: specify whether sequencing is Single-End or Paired-End, and the number of cycles *****

To specify Single-End vs. Paired-End format and number of cycles, enter text directly at the command line,
on a single line. Indicate Single-End (SE) or Paired-End (PE), followed by the number of cycles for each read.
Separate values by comma(s).

When text is entered, press ‘Enter’ again to proceed in the script.

Examples:
If you are performing a Paired-End run with 151 cycles in reads 1 & 2, enter
'PE, 151, 151'.

If you are performing a Single-End run with 151 cycles in read 1, enter
'SE, 151'.

----> [Reads] details:
```

5.

```
.................................................................................
***** [Data] details: specify relationships between sample names and barcodes. *****

You will now be asked to enter a text-only table of plate names with their corresponding
i7 barcode range and i5 barcode, for dual-indexed (Paired-End) sequencing.

Specifics:
  * Each line corresponds to samples barcoded across a single 96-well plate.
  * Each line is expanded in the output Sample Sheet based on the 'plate name' and specified number of barcoded
    sample wells; individual wells are assigned unique sample IDs in the Sample Sheet based on their unique
    combination of 'plate name' and 'i7 barcode' well ID.
  * 'Plate name' identifies a single 96-well plate identifier and must be unique.
  * Any letter, digit, and punctuation characters are acceptable in names, excluding underscores ('_') which must *not*
    be used.
  * 'i7 barcode' identifies an individual well (entry will be a numeric range, any # range up to '1-96')
  * 'i5 barcode' identifies all wells in a single plate (entry will be a single number, any # in '1' to '96')
  * For i7 and i5 barcode identifiers, use only the *range* (e.g., '1-96') or *number* (e.g., '1'-'96') that corresponds
    to a given index. Refer to i5 and i7 96-well plate sequences (displayed earlier as console PLATEVIEWS),if needed.
  * Fields (plate name, i7 index range, i5 index) are comma-separated.
  * You may manually enter or paste up to 96 lines that specify sample name-barcode relationships.
  * If entering lines individually at the command line, press Enter at the end of each line to move on to the next line.
  * When you press Enter twice, the prompt will consider your data entry complete.

Example: imagine that you have 310 samples barcoded across four 96-well plates (some plates containing 96 samples, some
plates containing fewer than 96 samples). Plate 1 used unique i7 barcodes '1-96' ('i7A01-i7H12') across 96 samples + i5
barcode '1' ('i5A01') for all 96 samples; Plate 2 used the same i7 barcode range + i5 barcode '9' ('i5A09') for its 96
samples, Plate 3 used unique barcodes '1-50' ('i7A01-i7E02') across 50 samples + i5 barcode '78' ('i5G06') for all 50
samples; Plate 4 used unique barcodes '1-68' ('i7A01-i7F08') across 68 samples + i5 barcode '34' ('i5C10') for all 68
samples.

You would enter text, line by line at the command line, that resembles this:
    DG-1, 1-96, 1
    DG-2, 1-96, 9
    DG-3, 1-50, 78
    DG-4, 1-68, 34

When you're done entering plates and their indices, press 'Enter' again to proceed in the script.

-----> [Data] details:
```

(b) Genotypes.py

List *vs.* Prompt

```
--------------------------------------------------------------------
User-specified input: choice of coached prompts vs. single-list entry
--------------------------------------------------------------------

Values for the user-specified input indicated above can be entered at individually coached command-line prompts,
or as a single list of variables provided in a single command-line entry without coached prompts.

To proceed with input at individual command-line PROMPTS, type 'Prompt' and press Enter;
To proceed with input provided as a single LIST in one command-line entry, type 'List' and press Enter:
```

Prompt

1.

```
--------------------------------------------
Location of OUTPUT DIRECTORY for output files
--------------------------------------------

The script generates 8 separate files, all in the directory you indicate here.  It is important that this directory
either not exist prior to running the script, or if it does exist, it must be *empty* of any files with the names to be
created below.  These files are:

    1. fasta.fa

    2. blastn_alignments.txt
        (output of BLASTN operation on fasta.fa)

    3. allele_definitions.txt
        (output of script operation on blastn_alignments.txt, samples returned in order of processing)

    4. allele_evidence.pdf (optional)
        (output of script operation on blastn_alignments.txt, plot of calculated read/allele frequencies)

    5. genotypes.txt
        (output of script operation on blastn_alignments.txt, samples returned in order of genotype inference)

    6. population_summary.txt
        (output of script operation on genotypes.txt)

    7. allele_definitions.csv
        (allele metrics (frequency representations) and definitions for each sample, in spreadsheet format)

    8. script_metrics.txt
        (summary/analysis of script operation metrics)

        Notes:
        * These files do not exist before the script is run. The files are made by the script.
        * The primary data outputs for genotypes are found in:
            allele_definitions.txt, allele_evidence.pdf, genotypes.txt & population_summary.txt


At this prompt, indicate an absolute path to a ** directory ** that will be created by the script as the location
for output files.  This directory should not exist yet -- it will be created as an output of this script, and will
be populated with the file outputs of this specific instance of the script operation.

Example: if you'd like to create a directory ('Genotypes') in an existing directory ('Illumina'), accessed
with absolute path of '/Users/myname/Illumina/Genotypes' (Mac) or 'C:\Users\myname\Illumina\Genotypes'
(Windows), enter '/Users/myname/Illumina/Genotypes' at the command line prompt. Replace 'myname' with the
appropriate intervening directory identifiers. Do *not* flank your entry with quotation marks (') at the
command line.

Alternatively, simply enter a desired directory name (e.g., 'Genotypes') and run this script from
within a directory where you'd like to create this new directory.

-----> Output directory name and path:
```

2.

```
-----------------------------------------------------------------------------
Location of INPUT FILES (single directory containing demutiplexed fastq files)
-----------------------------------------------------------------------------

You will now be asked to enter the path to the directory containing the fastq files
to be processed as Genotypes.py input.

Example: if your fastq input files are named file1.fastq, file2.fastq, etc. and are found in a directory
named 'Sequences' with absolute path of '/Users/myname/Sequences' (Mac) or 'C:\Users\myname\Sequences' (PC),
enter '/Users/myname/Sequences' at the command line prompt.

When you're done entering the fastq file location, press 'enter' again to proceed in the script.

-----> Directory name and path:
```

3.

```
-----------------------------
Location of BLASTN EXECUTABLE
-----------------------------

This script uses BLASTN (NCBI) to align reads from your fastq files to a reference sequence database
(such as a genome database or sequence database).
Please indicate the absolute path to the BLASTN executable.

Example: if your BLASTN executable is found at absolute path /Users/myname/blastn, type '/Users/myname/blastn'
and press Enter.

-----> Path to BLASTN executable:
```

4.

```
-----------------------------------------------
Location of BLASTN ALIGNMENT DATABASE DIRECTORY
-----------------------------------------------

Because this script uses BLASTN (NCBI) to align reads from your fastq files a reference sequence database,
an alignment reference database is needed. This reference database consists of a single directory containing
six files (.nhr, .nin, .nog, .nsd, .nsi, .nsg), generated by the program MAKEBLASTDB (NCBI) from a custom file
containing sequences in fasta format (or available for some genomes as downloads from NCBI).

Please indicate the absolute path to the directory you are using as your reference sequence database.

Example: if your reference sequence database is found at absolute path /Users/myname/database, type
'/Users/myname/database' and press Enter.

-----> Path to BLASTN alignment reference sequence database:
```

5.

```
------------------------------------------------
PREFIX common to BLASTN ALIGNMENT DATABASE FILES
------------------------------------------------

A BLASTN reference sequence database consists of six files in a single directory, with each of the six
files sharing a common prefix (usually determined by the name of the fasta file provided to MAKEBLASTDB
during database generation).

Please indicate the common prefix for files of the reference sequence database.

-----> Prefix for alignment reference sequence database files:
```

6.

```
--------------------------------
Location of BLASTDBCMD EXECUTABLE
--------------------------------

This script uses BLASTDBCMD (NCBI) to retrieve DNA sequence spans between coordinates in a reference sequence database*
(such as a genome database or sequence database) (*in cases where BLASTN has split an alignment into >1 local high-
scoring pair (hsp)).

Please indicate the absolute path to the BLASTDBCMD executable.

Example: if your BLASTDBCMD executable is found at absolute path /Users/myname/blastdbcmd,
type '/Users/myname/blastdbcmd' and press Enter.

-----> Path to BLASTDBCMD executable:
```

7-8.

```
---------------------------------------------------------------
Optional: Nucleotide sequence(s) to identify in output alignments
---------------------------------------------------------------

Some applications of 'allele definition' and 'genotype inference' may call for identification of the presence
or absence of a specific anticipated sub-sequence (few nucleotides), and/or for the mapping of the location of
a sub-sequence if present in the sequence alignment ('sequence of interest').

Genotypes.py allows for the optional testing of sub-sequences.

If you would like to specify subsequences, type 'Yes' and press Enter.
Otherwise, if you do not wish to specify subsequences, type 'No' and press Enter.

-----> 'Yes' or 'No' to sub-sequence specification:
```

7.

```
-----------------------------------------------------------------------------------------------
Nucleotide sequence(s): guide RNA annealing sites and/or test for presence/absence of sub-sequence
-----------------------------------------------------------------------------------------------

............................................................
***** guide RNA details: specify guide RNA sequence(s) *****

To specify guide RNA sequence(s), enter text for each directly at the command line,
separated by a comma ('x,y').

Please specify guide RNA sequence(s) [excluding PAM]:

When text entries are entered, press ‘enter’ again to proceed in the script.
To skip text entries for these fields, simply press ‘enter’ until the next prompt appears.

Examples:
    If your single guide RNA sequence is 'ATCCAGTTCTCCAGTCTCCC', enter: 'ATCCAGTTCTCCAGTCTCCC'.
    If you have two guide RNA sequences and they are 'ATCCAGTTCTCCAGTCTCCC' and 'GCGAGCTCGTGTCTGTGACG',
    enter: 'ATCCAGTTCTCCAGTCTCCC, GCGAGCTCGTGTCTGTGACG'.

-----> guide RNA sequence(s):
```

8.

```
..................................................................................
***** query DNA sequence(s): specify sequence(s) to test for presence or absence *****

To specify query DNA sequence(s), enter text for each directly at the command line,
separated by a comma ('x,y').

Please specify short DNA sequence to test for presence vs. ablation.

When text entries are entered, press ‘enter’ again to proceed in the script.
To skip text entries for these fields, simply press ‘enter’ until the next prompt appears.

Examples:
    If your single query sequence is 'TACTCAATATCGATC', enter: 'TACTCAATATCGATC'.
    If you have two query sequences and they are 'TACTCAATATCGATC' and 'CGGGAGCCCGAG', enter:
    'TACTCAATATCGATC, CGGGAGCCCGAG'.

-----> query DNA sequence(s):
```

List

```
----------------------------------
User-specified input (list format)
----------------------------------
Please paste a single list of input values directly at the command line prompt, specifying the following 7 values.
Press 'Enter' twice to complete.

1-Location of OUTPUT DIRECTORY for output files
2-Location of INPUT FILES (directory containing fastq files)
3-Location of BLASTN EXECUTABLE
4-Location of BLASTN ALIGNMENT DATABASE DIRECTORY
5-Prefix common to BLASTN sequence database files
6-Location of BLASTDBCMD EXECUTABLE
7-Optional guide RNA sequence(s) to identify in output alignments
8-Optional sub-sequence(s) to identify in output alignments
```

Include or Bypass frequency plot generation (allele_evidence.pdf)

```
Genotypes.py is ready to process fastq files. Before script operations begin, please indicate whether visual
plots of allele frequencies should be rendered and delivered in an output file, allele_evidence.pdf.

Note that production of allele_evidence.pdf can require hours of processing time, although the output timing of
key text files with allele definitions and genotype inferrals (e.g., allele_definitions.txt, genotypes.txt,
allele_definitions.csv, population_summary.txt) will not be affected.

To PROCEED with script operations that INCLUDE allele_evidence.pdf, type 'Y';

To BYPASS script operations that generate allele_evidence.pdf, type 'N':
```

(c) CollatedMotifs.py

List *vs.* Prompt

```
--------------------------------------------------------------------
User-specified input: choice of coached prompts vs. single list entry
--------------------------------------------------------------------

Values for the user-specified input indicated above can be entered at individually coached command-line prompts
(default), or as a single list of variables provided in a single command-line entry without coached prompts.

To proceed with input at individual command-line PROMPTS, type 'Prompt' and press Enter;
To proceed with input provided as a single LIST in one command-line entry, type 'List' and press Enter:

-----> List or Prompt:
```

Prompt

1.

```
---------------------------------------------
Location of OUTPUT DIRECTORY for output files
---------------------------------------------

This script produces 5 output files in the user-specified output directory, plus three directories:
two directories and subsidiary files created by FIMO (fimo_out and fimo_out_ref) and one directory
and subsidiary files created by MAKEBLASTDB (alignment_database).

CollatedMotifs.py output files include:

    1. fasta.fa

    2. blastn_alignments.txt
        (output of BLASTN operation on fasta.fa)

    3. markov_background.txt
        (output of FASTA-GET-MARKOV operation on user-supplied fasta reference file)

    4. collated_TFBS.txt
        (output of script operation on FIMO-generated .tsv files in fimo_out and fimo_out_ref)

    5. script_metrics.txt (summary/analysis of script operation metrics [metadata])

        Note:
        * These files do not exist before the script is run. The files are made by the script.
        * The primary data outputs for TFBS comparisons are found in collated_TFBS.txt

At this prompt, indicate an absolute path to a ** directory ** that will be created by the script as the location
for output files.  This directory should not exist yet -- it will be created as an output of this script, and will
be populated with the file outputs of this specific instance of the script operation.

Use only forward slashes ('/') as directory separators.

Example: if you'd like to create a directory ('CollatedMotifs') in an existing directory ('Illumina'), accessed
with absolute path of '/Users/myname/Illumina/CollatedMotifs' (Mac) or 'C:\Users\myname\Illumina\CollatedMotifs'
(Windows), enter '/Users/myname/Illumina/CollatedMotifs' at the command line prompt. Replace 'myname' with the
appropriate intervening directory identifiers. Do *not* flank your entry with quotation marks (') at the
command-line.

Alternatively, simply enter a desired directory name (e.g., 'CollatedMotifs') and run this script from
within a directory where you'd like to create this new directory.

-----> Output directory name and path:
```

2.

```
------------------------------------------------------------------------------
Location of INPUT FILES (single directory containing demutiplexed fastq files)
------------------------------------------------------------------------------

You will now be asked to enter the path to the directory containing the fastq files
to be processed as CollatedMotifs.py input.

Example: if your fastq input files are named file1.fastq, file2.fastq, etc. and are found in a directory
named 'Sequences' with absolute path of '/Users/myname/Sequences' (Mac) or 'C:\Users\myname\Sequences' (PC),
enter '/Users/myname/Sequences' at the command line prompt.

When you're done entering the fastq file location, press 'Enter' again to proceed in the script.

-----> Directory name and path:
```

3.

```
-----------------------------------------
Location of FIMO REFERENCE SEQUENCES FILE
-----------------------------------------

This script aligns and compares your top sample read sequence(s) to a defined reference sequence,
as its basis for determining distinct vs. common TFBS motifs. Please indicate the absolute path to a
fasta file containing reference sequence(s).

Use only forward slashes ('/') as directory separators.

**Important**: Each fasta entry definition line (defline) should be named such that the defline name
matches a unique descriptor (character string) that occurs in the fastq file names for samples that
will be aligned and compared to the corresponding fasta entry. For all ranked alleles for a given sample,
the fastq file name is incorporated into the allele names; the script then relies on a character string
to match between the allele name and an entry in the fasta reference file, to understand which reference
sequence to use for alignment and comparison of TFBSs that occur between the allele and the reference.

Example: if you have samples screened by PCR amplification across three distinct loci (Locus1, Locus2,
and Locus3), the fastq file names might be named Locus1_A01.fastq, Locus1_A02.fastq, etc.; Locus2_A01.fastq,
Locus2_A02.fastq, etc.; Locus3_A01.fastq, Locus3_A02.fastq, etc.

For the fasta reference sequences, you would designate deflines for the three different reference sequences such
that the deflines are character strings with diagnostic matches to character strings that occur in the
corresponding sample fastq file names (such as 'Locus1', 'Locus2', 'Locus3' for the example sample sets above.
Prepare the reference sequences in fasta format, saved in a single text file.

    >Locus1
    GATCGACTAGAGCGAGCATTCATCATATCACGAGTAGCATCGACGTGCACGATCGATCGTAGCTAGCTAGTCATGCATGCATGCTAGATTCGAGCATGCATGCTAC
    >Locus2
    AGTAGCTGTGATGCTAGTCATCTAGCTAGCAGCGTAGCTAGCGATCGATCTAGAGCCGATCGATCGAGCATCTAGCTATCAGCGGCGGGATCATCTATCTACGGG
    >Locus3
    CGATGCAGCGCGATCGAGCGCGATCGATATTAGCATGCGCAGCTAGCTAGCTGGCGATCGATGCATGCTAGCTGTGTCAGTCGACGATCACACGATCACACTGTGTG

When you're done entering the list of reference sequences, press 'Enter' again to proceed in the script.

-----> Path to fasta file containing reference sequences:
```

4.

```
-----------------------------
Location of BLASTN EXECUTABLE
-----------------------------

This script uses BLASTN (NCBI) to align reads from your fastq files to a reference sequence database.
Please indicate the absolute path to the BLASTN executable.

Example: if your BLASTN executable is found at absolute path /Users/myname/blastn, type '/Users/myname/blastn'
and press Enter.

-----> Path to BLASTN executable:
```

5.

```
----------------------------------
Location of MAKEBLASTDB EXECUTABLE
----------------------------------

Because this script uses BLASTN (NCBI) to align reads from your fastq files to a reference sequence database,
a compatible reference sequence database is required. This script uses MAKEBLASTDB (NCBI) to generate
a reference sequence database from the reference sequences in the fasta file you provided earlier.

Please indicate the absolute path to the MAKEBLASTDB executable.

Example: if your MAKEBLASTDB executable is found at absolute path /Users/myname/makeblastdb,
type '/Users/myname/makeblastdb' and press Enter.

-----> Path to MAKEBLASTDB executable:
```

6.

```
---------------------------------------------
Prefix for files in BLASTN ALIGNMENT DATABASE
---------------------------------------------

Because this script uses BLASTN (NCBI) and an alignment reference database, a common prefix identifier for the six
database files generated by MAKEBLASTDB is needed.

Please indicate a prefix to assign to each of the database files.

Example: if your alignment reference was generated by MAKEBLASTDB from a fasta file called GRCh38.fa,
the alignment database files will have been assigned the prefix 'GRCh38'; you would type 'GRCh38'
and press Enter.

-----> Prefix for alignment reference sequence database files:
```

7.

```
---------------------------
Location of FIMO EXECUTABLE
---------------------------

This script uses FIMO from the MEME suite of sequence analysis tools as its basis for determining distinct vs.
common TFBSs.
Please indicate the absolute path to the FIMO installation.

Example: if your FIMO executable is found at absolute path /Users/myname/fimo, type '/Users/myname/fimo'
and press Enter.

-----> Path to FIMO executable:
```

8.

```
----------------------------
Location of FIMO MOTIFS FILE
----------------------------

This script uses FIMO from the meme suite of sequence analysis tools as its basis for determining distinct vs.
common TFBS motifs.
Please indicate the absolute path to the FIMO motifs file (containing position frequency matrix/matrices).

When you're done entering the location of the motifs file, press Enter.

-----> Path to FIMO motifs file:
```

9.

```
---------------------------------------------
Location of FIMO FASTA-GET-MARKOV EXECUTABLE
---------------------------------------------

This script uses FIMO from the MEME suite of sequence analysis tools as its basis for determining distinct vs.
common TFBSs.
Please indicate an absolute path to the location of the FASTA-GET-MARKOV executable.

When you're done entering the location of the executable, press Enter.

-----> Path to FASTA-GET-MARKOV executable:
```

10.

```
-------------------------------------------------------------
Location of FIMO FASTA-GET-MARKOV BACKGROUND REFERENCE FILE
-------------------------------------------------------------

This script uses FIMO from the MEME suite of sequence analysis tools as its basis for determining distinct vs.
common TFBSs.
Please indicate an absolute path to the location of the fasta file you will use as your background reference
(on which FASTA-GET-MARKOV will operate to generate a markov background file).

When you're done entering the location of the reference sequence, press Enter.

-----> Path to background reference file:
```

11.

```
-------------------------------------------------
TRANSCRIPTION FACTOR (TF) of interest (optional)
-------------------------------------------------

This script collates lost and gained TFBS for sample-associated allele(s) relative to a reference sequence;
if detailed analysis of alleles that have lost TFBS matches for a specific transcription factor (TF) are desired,
the identity of an individual TF of interest can be provided (optional).

If you would like the script to further analyze alleles for TFBS matches to a specific TF, please indicate the
TF here.  Otherwise, press Enter.

Important: Use only the standardized Entrez gene name for the TF of interest (such as NR3C1), rather than the
matrix model stable ID (for example, MA0113 for NR3C1) or stable ID with version number (for example, MA0113.3
for NR3C1).

Example: if you are interested in losses of TFBS for the TF NR3C1, you would type 'NR3C1' and press Enter.
```

List

```
----------------------------------
User-specified input (list format)
----------------------------------

Please paste a single list of input values directly at the command line prompt, specifying the following 10 values.
Press 'Enter' twice to complete.

1-Location of OUTPUT DIRECTORY for output files
2-Location of INPUT FILES (directory containing fastq files)
3-Location of REFERENCE FASTA FILE
4-Location of BLASTN EXECUTABLE
5-Location of MAKEBLASTDB EXECUTABLE
6-Prefix common to BLASTN sequence database files
7-Location of FIMO EXECUTABLE
8-Location of POSITION FREQUENCY MATRIX FILE
9-Location of FASTA-GET-MARKOV EXECUTABLE
10-Location of MARKOV BACKGROUND FILE
11-Identity of TRANSCRIPTION FACTOR (TF) of interest (optional)
```

Supplementary Figure S4. Illumina® Sequencing by Synthesis (SBS) workflows A & B: distinct orders of operation determine whether i7 & i5 indices are read in the same orientation as the sequenced read or in antiparallel fashion (reverse complement).

Dual-indexed (paired-end) DNA sequencing on Illumina® instruments can use one of two Workflows (A or B), defined based on whether re-synthesis of complementary DNA template strands (for read 2) takes place before (*Workflow A)* or after (*Workflow B*) i5 (index 2) sequencing. Awareness of the Workflow customary to a sequencing application is important in Sample Sheet [Data] entry, because Workflow determines whether an index sequence is provided to demultiplexing software as the 'forward' sequence or its 'reverse complement'. In the MiSeq application reported as an Example Case Use (**Fig. 5**), we used the Illumina® MiSeq (MiSeq Reagent Kit v2: PE, 2x150 bp)—an instrument that applies *Workflow A*. In the Sample Sheet [Data] table, we therefore chose 'Workflow A' at the appropriate SampleSheet.py console prompt, and the script automatically populated i7 barcode sequences as *reverse complements* relative to barcode sequences as they appear in the i7 primers used in library construction, and i5 barcode sequences as *forward* orientations relative to barcode sequences as they appear in the i5 primers used in library construction (**Supp. Fig. 1c, Supp. Fig. 3b**).

(a) As of June 2021, Illumina® MiSeq, NovaSeq 6000, HiSeq 2500, and HiSeq 2000 instruments use Workflow A, where i7 index sequence is recovered as 'reverse complement' relative to i7 sequence as it occurs in primers used for library construction and i5 index sequence is recovered as it occurs in primers used for library construction;

(b) iSeq100, MiniSeq, NextSeq, HiSeqX, HiSeq 4000, and HiSeq 3000 instruments use Workflow B, where both i7 and i5 index sequences are recovered as 'reverse complements' relative to i7 and i5 sequences as they occur in primers used for library construction.

*See **Supp. Fig. 5b** for definitions of color-coded amplicon subregions.*

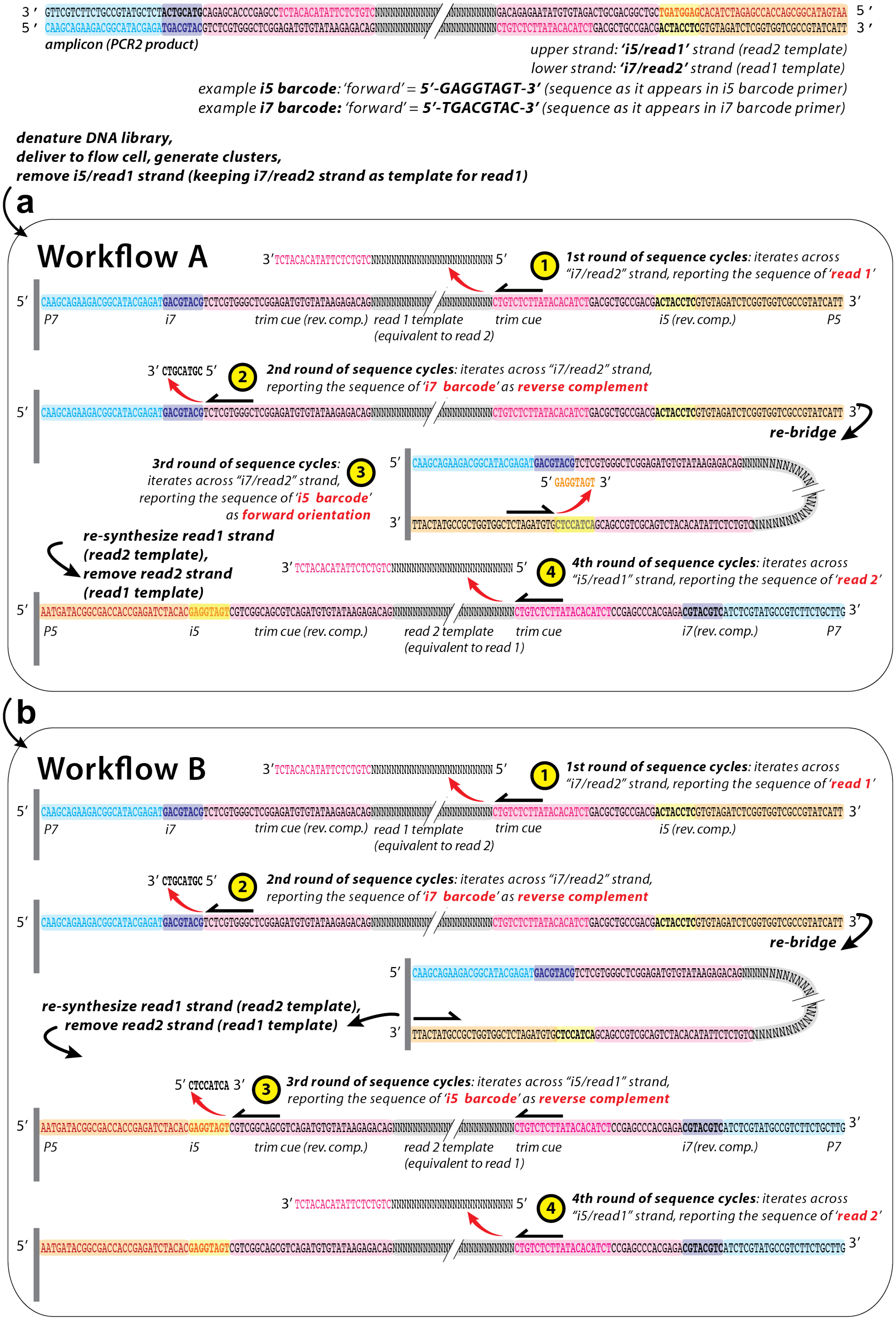
3′
5′
amplicon (PCR2 product)
upper strand: 'i5/read1' strand (read2 template)
lower strand: 'i7/read2' strand (read1 template)
example i5 barcode: 'forward' = 5′-GAGGTAGT-3′ (sequence as it appears in i5 barcode primer)
example i7 barcode: 'forward' = 5′-TGACGTAC-3′ (sequence as it appears in i7 barcode primer)
denature DNA library,
deliver to flow cell, generate clusters,
remove i5/read1 strand (keeping i7/read2 strand as template for read1)
a
Workflow A
1st round of sequence cycles: iterates across "i7/read2" strand, reporting the sequence of 'read 1'
P7
i7
trim cue (rev. comp.)
read 1 template (equivalent to read 2)
trim cue
i5 (rev. comp.)
P5
3′ CTGCATGC 5′
2nd round of sequence cycles: iterates across "i7/read2" strand, reporting the sequence of 'i7 barcode' as reverse complement
re-bridge
3rd round of sequence cycles: iterates across "i7/read2" strand, reporting the sequence of 'i5 barcode' as forward orientation
5′ GAGGTAGT 3′
re-synthesize read1 strand (read2 template), remove read2 strand (read1 template)
4th round of sequence cycles: iterates across "i5/read1" strand, reporting the sequence of 'read 2'
P5
i5
trim cue (rev. comp.)
read 2 template (equivalent to read 1)
trim cue
i7 (rev. comp.)
P7
b
Workflow B
1st round of sequence cycles: iterates across "i7/read2" strand, reporting the sequence of 'read 1'
P7
i7
trim cue (rev. comp.)
read 1 template (equivalent to read 2)
trim cue
i5 (rev. comp.)
3′ CTGCATGC 5′
2nd round of sequence cycles: iterates across "i7/read2" strand, reporting the sequence of 'i7 barcode' as reverse complement
re-bridge
re-synthesize read1 strand (read2 template), remove read2 strand (read1 template)
5′ CTCCATCA 3′
3rd round of sequence cycles: iterates across "i5/read1" strand, reporting the sequence of 'i5 barcode' as reverse complement
P5
i5
trim cue (rev. comp.)
read 2 template (equivalent to read 1)
trim cue
i7 (rev. comp.)
P7
4th round of sequence cycles: iterates across "i5/read1" strand, reporting the sequence of 'read 2'

Supplementary Figure S5. Overview of PCR1 & 2 for amplicon sequencing.

Library preparation for Illumina® sequencing often involves *i)* fragmentation of a DNA or cDNA sample to 100-1000 bp, with 5' and 3' (P5 & P7) adapter ligation, or *ii)* combined fragmentation and ligation ('tagmentation'; Nextera workflows), followed by PCR-amplification and clean-up. In amplicon sequencing as described here, sequencing libraries are prepared by two-step PCR (PCR1 + PCR2).

(a) **PCR1** produces the target amplicon (future read 1 and read2 zones) with flanking sequence that introduces Nextera adaptor sequences ('trim cues'). PCR1 requires custom-designed oligos specific to a target locus, with specific sample source as DNA template (*e.g.*, genomic DNA lysate). *Note:* for design of amplicons to sequence across region(s) targeted for mutagenesis triggered by programmable nucleases, it is recommended to design the amplicon to approximately straddle the position of the anticipated incision;

(b) **PCR2** uses a small amount of PCR1 amplicon as input, and i5 and i7 barcode oligos (96x96 possibilities for dual indexing) as primers that add P5 and i5 index on one end of the product amplicon, and P7 and i7 index on the other end of the product amplicon.

PCR2 product can be pooled from multiple independent samples, and after SPRI clean-up from unincorporated primers, the pooled amplicons represent a library ready to be quantified and applied to an Illumina® flow cell for deep sequencing. Denatured library templates anneal to surface-bound oligos complementary to the P7 & P5 library adapters, and are locally amplified into physically separate clusters by bridge amplification; after sequencing, data processing includes demultiplexing of barcoded reads, trimming of adaptor sequences from reads ('trim cues') if adaptor template was encroached during sequencing, and documentation of quality scores for each sequenced nucleotide, all output to individual fastq files (Illumina® Pub. No. 770-2012-008-B (2017)).

Note regarding P5, P7, & Nextera adapter sequences (in concordance with Illumina® Document #1000000002694 v10, Feb. 2019): *Oligonucleotide sequences © 2018 Illumina®, Inc. All rights reserved. Derivative works created by Illumina® customers are authorized for use with Illumina® instruments and products only. All other uses are strictly prohibited.*

a

PCR 1

‘read2 oligo’ [aka ‘rev’]
(sequence contiguous with 5’->3’ bases that occur in this oligo is future read2/future read1 template)

5 ’ – TCGGAGATGTGTATAAGAGACAG TTACTGCTGTGCACTCTCTTTCC – 3 ’
Nextera read2 addition *target-specific sequence (example is for FKBP5 GOR +86 kb)*

‘read1 oligo’ [aka ‘fwd’]
(sequence contiguous with 5’->3’ bases that occur in this oligo is future read1/future read2 template)

5 ’ – TCAGATGTGTATAAGAGACAG ACTTAAACTGGAGCTCTGACTTATTGTTC – 3 ’
Nextera read1 addition *target-specific sequence (example is for FKBP5 GOR +86 kb)*

amplicon product:

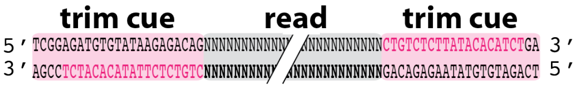

expected size (bp):

+23 bp [target amplicon length] +21 bp = [target amplicon length] +44 bp

b

PCR 2

‘i7 oligo’ [aka ‘read2_bc_1_TGACGTAC’]
(sequence contiguous with 5’->3’ bases that occur in this oligo is future read2/future read1 template)

5 ’ – CAAGCAGAAGACGGCATACGAGATTGACGTACGTCTCGTGGGCTCGGAGATGTGTATAAGAGACAG – 3 ’
P7 i7
P7 & i7 addition *PCR1 amplicon product overlap*

‘i5 oligo’ [aka ‘read1_bc_1_GAGGTAGT’]
(sequence contiguous with 5’->3’ bases that occur in this oligo is future read1/future read2 template)

5 ’ – AATGATACGGCGACCACCGAGATCTACACGAGGTAGTTCGTCGGCAGCGTCAGATGTGTATAAGAGACAG – 3 ’
P5 i5
P5 & i5 addition *PCR1 amplicon product overlap*

amplicon product:

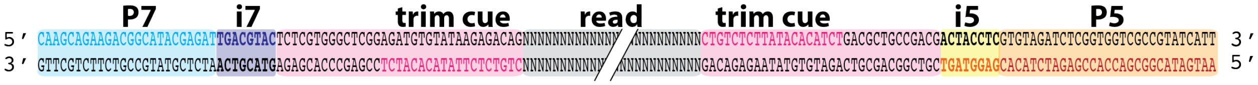

expected size (bp):

+42 bp [PCR1 amplicon length] +48 bp = [PCR1 amplicon length] +90 bp
([target amplicon length]+134 bp)

Supplementary Figure S6. Command line interface 'plateviews' of barcode sequences (i7 & i5).

CLI Plateviews of i7 & i5 primer sets available to users of SampleSheet.py, with PrettyTable installation. The integers 1-96 above the barcode ID (*e.g.*, i7B03) designate barcode identifiers, and are the barcode labels specified by a user as input when prompted. For example, the integer '15' entered at the console specifies 'i7B03' (barcode sequence 'GTGTCCTA' in Workflow A). SampleSheet.py anticipates i7 barcodes to specify up to 96 individual wells of a plate, and i5 barcodes to specify individual plates (up to 96 separate 96-well plates). Sequences displayed (and rendered in Sample Sheet [Data] fields) correspond to 'forward' or 'reverse complement' orientations, defined by Workflow A or B.

Command line interface:

Workflow A: i5 'forward', i7 'reverse complement'

Please note, each 8-bp barcode sequence as displayed in this table is the sequence to be used in a Workflow A Sample Sheet barcode field.
The displayed sequence is the reverse complement of the barcode sequence as it occurs in the i7 primer.

	1	2	3	4	5	6	7	8	9	10	11	12
A	1<br>i7A01<br>GTACGTCA	2<br>i7A02<br>TGCAGTTA	3<br>i7A03<br>ACTGTGGA	4<br>i7A04<br>GGTTAAGA	5<br>i7A05<br>TCACACTA	6<br>i7A06<br>TAGAGGTA	7<br>i7A07<br>GCGACATA	8<br>i7A08<br>TACATGCA	9<br>i7A09<br>GATGATGA	10<br>i7A10<br>TGTGTGCA	11<br>i7A11<br>TCGCTACA	12<br>i7A12<br>AAGCTAGA
B	13<br>i7B01<br>TAGGACCA	14<br>i7B02<br>TCGTTGGA	15<br>i7B03<br>GTGTCCTA	16<br>i7B04<br>TCCGTATA	17<br>i7B05<br>TACGCTTA	18<br>i7B06<br>TACAACGA	19<br>i7B07<br>GAAGGAGA	20<br>i7B08<br>AAGCATCA	21<br>i7B09<br>TGAGATCA	22<br>i7B10<br>TCTAGACA	23<br>i7B11<br>TGCTCATA	24<br>i7B12<br>TAACTCCA
C	25<br>i7C01<br>GACACACA	26<br>i7C02<br>TTGTAGCA	27<br>i7C03<br>GTCTACGA	28<br>i7C04<br>GCATTACA	29<br>i7C05<br>GTTCCATA	30<br>i7C06<br>GAATACCA	31<br>i7C07<br>TCACTTGA	32<br>i7C08<br>ACACAAGA	33<br>i7C09<br>ACTACAGA	34<br>i7C10<br>TCTCAGGA	35<br>i7C11<br>ATCGTGCA	36<br>i7C12<br>TCCACGTA
D	37<br>i7D01<br>ATCCATGA	38<br>i7D02<br>TTCGCAGA	39<br>i7D03<br>TCGTCTCA	40<br>i7D04<br>ACTTACGA	41<br>i7D05<br>TCGGATTA	42<br>i7D06<br>TTGGTCTA	43<br>i7D07<br>ATAGCGGA	44<br>i7D08<br>TGTAACCA	45<br>i7D09<br>ACAGGCTA	46<br>i7D10<br>AAGTCGCA	47<br>i7D11<br>GAGTTCGA	48<br>i7D12<br>ACTCTTCA
E	49<br>i7E01<br>AAGACCTA	50<br>i7E02<br>ACCATCCA	51<br>i7E03<br>GAACGGTA	52<br>i7E04<br>TAGTGAGA	53<br>i7E05<br>GTCCAACA	54<br>i7E06<br>TATGGCGA	55<br>i7E07<br>GCTTATCA	56<br>i7E08<br>GTGACTGA	57<br>i7E09<br>ACGTGATA	58<br>i7E10<br>GGTTCTTA	59<br>i7E11<br>GATCAGCA	60<br>i7E12<br>GGACAATA
F	61<br>i7F01<br>AACTCTGA	62<br>i7F02<br>TGACCTTA	63<br>i7F03<br>GGAAGTGA	64<br>i7F04<br>AAGAAGGA	65<br>i7F05<br>GCAATCTA	66<br>i7F06<br>ACGAGTCA	67<br>i7F07<br>TTATGCCA	68<br>i7F08<br>GCCATAGA	69<br>i7F09<br>TAATCGGA	70<br>i7F10<br>TCTCGTTA	71<br>i7F11<br>GGTCTGTA	72<br>i7F12<br>ATCCGGTA
G	73<br>i7G01<br>ATGCGACA	74<br>i7G02<br>TGAACGCA	75<br>i7G03<br>TGGTATGA	76<br>i7G04<br>TGGACAGA	77<br>i7G05<br>TTGCAAGA	78<br>i7G06<br>ACTAGGTA	79<br>i7G07<br>TACCGATA	80<br>i7G08<br>ATTGGTGA	81<br>i7G09<br>TTCCTCGA	82<br>i7G10<br>TCATGGTA	83<br>i7G11<br>TATCCACA	84<br>i7G12<br>GACTTGTA
H	85	86	87	88	89	90	91	92	93	94	95	96

Please note, each 8-bp barcode sequence as displayed in this table is the sequence to be used in a Workflow A Sample Sheet barcode field.
The displayed sequence is identical to the barcode sequence as it occurs in the i5 primer.

	1	2	3	4	5	6	7	8	9	10	11	12
A	1<br>i5A01<br>GAGGTAGT	2<br>i5A02<br>GCTTAACT	3<br>i5A03<br>GCAATTCT	4<br>i5A04<br>TCCTCACT	5<br>i5A05<br>AGTTAGCT	6<br>i5A06<br>TCATGGCT	7<br>i5A07<br>AGCCTAAT	8<br>i5A08<br>AACACTCT	9<br>i5A09<br>AGAGCTCT	10<br>i5A10<br>GGCCATAT	11<br>i5A11<br>TAGAACGT	12<br>i5A12<br>GGTTGAAT
B	13<br>i5B01<br>GATCCTAT	14<br>i5B02<br>TGGTCTCT	15<br>i5B03<br>TCCAGTCT	16<br>i5B04<br>TAGCGTAT	17<br>i5B05<br>GCGATCAT	18<br>i5B06<br>AGCTACAT	19<br>i5B07<br>ACTCATCT	20<br>i5B08<br>TTGCGAGT	21<br>i5B09<br>AAGCGACT	22<br>i5B10<br>TGCACGAT	23<br>i5B11<br>TATGCGGT	24<br>i5B12<br>TCGTCGAT
C	25<br>i5C01<br>ACACTTGT	26<br>i5C02<br>TACGGCAT	27<br>i5C03<br>TCTCGTGT	28<br>i5C04<br>GCAGATGT	29<br>i5C05<br>TCCGTTGT	30<br>i5C06<br>TTCGCAGT	31<br>i5C07<br>GGAATAGT	32<br>i5C08<br>GCAACAAT	33<br>i5C09<br>ACTGCGAT	34<br>i5C10<br>TGAGTACT	35<br>i5C11<br>ACACGAAT	36<br>i5C12<br>GTGGCAAT
D	37<br>i5D01<br>GTCATCCT	38<br>i5D02<br>ACCGTACT	39<br>i5D03<br>GTACTACT	40<br>i5D04<br>TAGGTCCT	41<br>i5D05<br>GTTACTGT	42<br>i5D06<br>GCCTTAGT	43<br>i5D07<br>AACCATGT	44<br>i5D08<br>GATGACAT	45<br>i5D09<br>AGAAGACT	46<br>i5D10<br>TCAAGCAT	47<br>i5D11<br>TGGATGCT	48<br>i5D12<br>ACTTGCCT
E	49<br>i5E01<br>GTTGTGAT	50<br>i5E02<br>TATCTCGT	51<br>i5E03<br>TGTAAGGT	52<br>i5E04<br>ACGTTGGT	53<br>i5E05<br>AGGAGGAT	54<br>i5E06<br>GAGCAGAT	55<br>i5E07<br>TCTCTACT	56<br>i5E08<br>GGATTCAT	57<br>i5E09<br>GACCTTCT	58<br>i5E10<br>ACGGTTAT	59<br>i5E11<br>AGATGCGT	60<br>i5E12<br>TGACACCT
F	61<br>i5F01<br>ACTAACGT	62<br>i5F02<br>GAATGCCT	63<br>i5F03<br>GTGTACGT	64<br>i5F04<br>AAGTCAGT	65<br>i5F05<br>TTCCTGGT	66<br>i5F06<br>ACCACCAT	67<br>i5F07<br>GAGTCCAT	68<br>i5F08<br>AGCTCTGT	69<br>i5F09<br>GGTCTTGT	70<br>i5F10<br>AATGTGCT	71<br>i5F11<br>GTCTGGAT	72<br>i5F12<br>GCGTATAT
G	73<br>i5G01<br>TGACTGAT	74<br>i5G02<br>TGTCCAGT	75<br>i5G03<br>TTGTGCCT	76<br>i5G04<br>ACAGACCT	77<br>i5G05<br>GATCAAGT	78<br>i5G06<br>GATAGGAT	79<br>i5G07<br>GTAGTCGT	80<br>i5G08<br>TCACCTAT	81<br>i5G09<br>AGTGGTAT	82<br>i5G10<br>GCTAGAGT	83<br>i5G11<br>TTAACGCT	84<br>i5G12<br>TCAGGAGT
H	85<br>i5H01<br>AACTGGCT	86<br>i5H02<br>TACAGAGT	87<br>i5H03<br>TTGCATCT	88<br>i5H04<br>GAACGTGT	89<br>i5H05<br>AAGTACCT	90<br>i5H06<br>GGTACCAT	91<br>i5H07<br>AATCGCAT	92<br>i5H08<br>GGTAATCT	93<br>i5H09<br>GATTCGCT	94<br>i5H10<br>AGCATCGT	95<br>i5H11<br>GTTCACCT	96<br>i5H12<br>AGGCAAGT

Workflow B: i5 'reverse complement', i7 'reverse complement'

Please note, each 8-bp barcode sequence as displayed in this table is the sequence to be used in a Workflow B Sample Sheet barcode field.
The displayed sequence is the reverse complement of the barcode sequence as it occurs in the i7 primer.

	1	2	3	4	5	6	7	8	9	10	11	12
A	1<br>i7A01<br>GTACGTCA	2<br>i7A02<br>TGCAGTTA	3<br>i7A03<br>ACTGTGGA	4<br>i7A04<br>GGTTAAGA	5<br>i7A05<br>TCACACTA	6<br>i7A06<br>TAGAGGTA	7<br>i7A07<br>GCGACATA	8<br>i7A08<br>TACATGCA	9<br>i7A09<br>GATGATGA	10<br>i7A10<br>TGTGTGCA	11<br>i7A11<br>TCGCTACA	12<br>i7A12<br>AAGCTAGA
B	13<br>i7B01<br>TAGGACCA	14<br>i7B02<br>TCGTTGGA	15<br>i7B03<br>GTGTCCTA	16<br>i7B04<br>TCCGTATA	17<br>i7B05<br>TACGCTTA	18<br>i7B06<br>TACAACGA	19<br>i7B07<br>GAAGGAGA	20<br>i7B08<br>AAGCATCA	21<br>i7B09<br>TGAGATCA	22<br>i7B10<br>TCTAGACA	23<br>i7B11<br>TGCTCATA	24<br>i7B12<br>TAACTCCA
C	25<br>i7C01<br>GACACACA	26<br>i7C02<br>TTGTAGCA	27<br>i7C03<br>GTCTACGA	28<br>i7C04<br>GCATTACA	29<br>i7C05<br>GTTCCATA	30<br>i7C06<br>GAATACCA	31<br>i7C07<br>TCACTTGA	32<br>i7C08<br>ACACAAGA	33<br>i7C09<br>ACTACAGA	34<br>i7C10<br>TCTCAGGA	35<br>i7C11<br>ATCGTGCA	36<br>i7C12<br>TCCACGTA
D	37<br>i7D01<br>ATCCATGA	38<br>i7D02<br>TTCGCAGA	39<br>i7D03<br>TCGTCTCA	40<br>i7D04<br>ACTTACGA	41<br>i7D05<br>TCGGATTA	42<br>i7D06<br>TTGGTCTA	43<br>i7D07<br>ATAGCGGA	44<br>i7D08<br>TGTAACCA	45<br>i7D09<br>ACAGGCTA	46<br>i7D10<br>AAGTCGCA	47<br>i7D11<br>GAGTTCGA	48<br>i7D12<br>ACTCTTCA
E	49<br>i7E01<br>AAGACCTA	50<br>i7E02<br>ACCATCCA	51<br>i7E03<br>GAACGGTA	52<br>i7E04<br>TAGTGAGA	53<br>i7E05<br>GTCCAACA	54<br>i7E06<br>TATGGCGA	55<br>i7E07<br>GCTTATCA	56<br>i7E08<br>GTGACTGA	57<br>i7E09<br>ACGTGATA	58<br>i7E10<br>GGTTCTTA	59<br>i7E11<br>GATCAGCA	60<br>i7E12<br>GGACAATA
F	61<br>i7F01<br>AACTCTGA	62<br>i7F02<br>TGACCTTA	63<br>i7F03<br>GGAAGTGA	64<br>i7F04<br>AAGAAGGA	65<br>i7F05<br>GCAATCTA	66<br>i7F06<br>ACGAGTCA	67<br>i7F07<br>TTATGCCA	68<br>i7F08<br>GCCATAGA	69<br>i7F09<br>TAATCGGA	70<br>i7F10<br>TCTCGTTA	71<br>i7F11<br>GGTCTGTA	72<br>i7F12<br>ATCCGGTA
G	73<br>i7G01<br>ATGCGACA	74<br>i7G02<br>TGAACGCA	75<br>i7G03<br>TGGTATGA	76<br>i7G04<br>TGGACAGA	77<br>i7G05<br>TTGCAAGA	78<br>i7G06<br>ACTAGGTA	79<br>i7G07<br>TACCGATA	80<br>i7G08<br>ATTGGTGA	81<br>i7G09<br>TTCCTCGA	82<br>i7G10<br>TCATGGTA	83<br>i7G11<br>TATCCACA	84<br>i7G12<br>GACTTGTA
H	85<br>i7H01	86<br>i7H02	87<br>i7H03	88<br>i7H04	89<br>i7H05	90<br>i7H06	91<br>i7H07	92<br>i7H08	93<br>i7H09	94<br>i7H10	95<br>i7H11	96<br>i7H12

Please note, each 8-bp barcode sequence as displayed in this table is the sequence to be used in a Workflow B Sample Sheet barcode field.
The displayed sequence is the reverse complement of the barcode sequence as it occurs in the i5 primer.

	1	2	3	4	5	6	7	8	9	10	11	12
A	1<br>i5A01<br>CTCCATCA	2<br>i5A02<br>CGAATTGA	3<br>i5A03<br>CGTTAAGA	4<br>i5A04<br>AGGAGTGA	5<br>i5A05<br>TCAATCGA	6<br>i5A06<br>AGTACCGA	7<br>i5A07<br>TCGGATTA	8<br>i5A08<br>TTGTGAGA	9<br>i5A09<br>TCTCGAGA	10<br>i5A10<br>CCGGTATA	11<br>i5A11<br>ATCTTGCA	12<br>i5A12<br>CCAACTTA
B	13<br>i5B01<br>CTAGGATA	14<br>i5B02<br>ACCAGAGA	15<br>i5B03<br>AGGTCAGA	16<br>i5B04<br>ATCGCATA	17<br>i5B05<br>CGCTAGTA	18<br>i5B06<br>TCGATGTA	19<br>i5B07<br>TGAGTAGA	20<br>i5B08<br>AACGCTCA	21<br>i5B09<br>TTCGCTGA	22<br>i5B10<br>ACGTGCTA	23<br>i5B11<br>ATACGCCA	24<br>i5B12<br>AGCAGCTA
C	25<br>i5C01<br>TGTGAACA	26<br>i5C02<br>ATGCCGTA	27<br>i5C03<br>AGAGCACA	28<br>i5C04<br>CGTCTACA	29<br>i5C05<br>AGGCAACA	30<br>i5C06<br>AAGCGTCA	31<br>i5C07<br>CCTTATCA	32<br>i5C08<br>CGTTGTTA	33<br>i5C09<br>TGACGCTA	34<br>i5C10<br>ACTCATGA	35<br>i5C11<br>TGTGCTTA	36<br>i5C12<br>CACCGTTA
D	37<br>i5D01<br>CAGTAGGA	38<br>i5D02<br>TGGCATGA	39<br>i5D03<br>CATGATGA	40<br>i5D04<br>ATCCAGGA	41<br>i5D05<br>CAATGACA	42<br>i5D06<br>CGGAATCA	43<br>i5D07<br>TTGGTACA	44<br>i5D08<br>CTACTGTA	45<br>i5D09<br>TCTTCTGA	46<br>i5D10<br>AGTTCGTA	47<br>i5D11<br>ACCTACGA	48<br>i5D12<br>TGAACGGA
E	49<br>i5E01<br>CAACACTA	50<br>i5E02<br>ATAGAGCA	51<br>i5E03<br>ACATTCCA	52<br>i5E04<br>TGCAACCA	53<br>i5E05<br>TCCTCCTA	54<br>i5E06<br>CTCGTCTA	55<br>i5E07<br>AGAGATGA	56<br>i5E08<br>CCTAAGTA	57<br>i5E09<br>CTGGAAGA	58<br>i5E10<br>TGCCAATA	59<br>i5E11<br>TCTACGCA	60<br>i5E12<br>ACTGTGGA
F	61<br>i5F01<br>TGATTGCA	62<br>i5F02<br>CTTACGGA	63<br>i5F03<br>CACATGCA	64<br>i5F04<br>TTCAGTCA	65<br>i5F05<br>AAGGACCA	66<br>i5F06<br>TGGTGGTA	67<br>i5F07<br>CTCAGGTA	68<br>i5F08<br>TCGAGACA	69<br>i5F09<br>CCAGAACA	70<br>i5F10<br>TTACACGA	71<br>i5F11<br>CAGACCTA	72<br>i5F12<br>CGCATATA
G	73<br>i5G01<br>ACTGACTA	74<br>i5G02<br>ACAGGTCA	75<br>i5G03<br>AACACGGA	76<br>i5G04<br>TGTCTGGA	77<br>i5G05<br>CTAGTTCA	78<br>i5G06<br>CTATCCTA	79<br>i5G07<br>CATCAGCA	80<br>i5G08<br>AGTGGATA	81<br>i5G09<br>TCACCATA	82<br>i5G10<br>CGATCTCA	83<br>i5G11<br>AATTGCGA	84<br>i5G12<br>AGTCCTCA
H	85<br>i5H01<br>TTGACCGA	86<br>i5H02<br>ATGTCTCA	87<br>i5H03<br>AACGTAGA	88<br>i5H04<br>CTTGCACA	89<br>i5H05<br>TTCATGGA	90<br>i5H06<br>CCATGGTA	91<br>i5H07<br>TTAGCGTA	92<br>i5H08<br>CCATTAGA	93<br>i5H09<br>CTAAGCGA	94<br>i5H10<br>TCGTAGCA	95<br>i5H11<br>CAAGTGGA	96<br>i5H12<br>TCCGTTCA

Jupyter Notebook interface:

<u>Workflow A: i5 'forward', i7 'reverse complement'</u>

	1	2	3	4	5	6	7	8	9	10	11	12
**A**	1<br>i7A01<br>GTACGTCA	2<br>i7A02<br>TGCAGTTA	3<br>i7A03<br>ACTGTGGA	4<br>i7A04<br>GGTTAAGA	5<br>i7A05<br>TCACACTA	6<br>i7A06<br>TAGAGGTA	7<br>i7A07<br>GCGACATA	8<br>i7A08<br>TACATGCA	9<br>i7A09<br>GATGATGA	10<br>i7A10<br>TGTGTGCA	11<br>i7A11<br>TCGCTACA	12<br>i7A12<br>AAGCTAGA
**B**	13<br>i7B01<br>TAGGACCA	14<br>i7B02<br>TCGTTGGA	15<br>i7B03<br>GTGTCCTA	16<br>i7B04<br>TCCGTATA	17<br>i7B05<br>TACGCTTA	18<br>i7B06<br>TACAACGA	19<br>i7B07<br>GAAGGAGA	20<br>i7B08<br>AAGCATCA	21<br>i7B09<br>TGAGATCA	22<br>i7B10<br>TCTAGACA	23<br>i7B11<br>TGCTCATA	24<br>i7B12<br>TAACTCCA
**C**	25<br>i7C01<br>GACACACA	26<br>i7C02<br>TTGTAGCA	27<br>i7C03<br>GTCTACGA	28<br>i7C04<br>GCATTACA	29<br>i7C05<br>GTTCCATA	30<br>i7C06<br>GAATACCA	31<br>i7C07<br>TCACTTGA	32<br>i7C08<br>ACACAAGA	33<br>i7C09<br>ACTACAGA	34<br>i7C10<br>TCTCAGGA	35<br>i7C11<br>ATCGTGCA	36<br>i7C12<br>TCCACGTA
**D**	37<br>i7D01<br>ATCCATGA	38<br>i7D02<br>TTCGCAGA	39<br>i7D03<br>TCGTCTCA	40<br>i7D04<br>ACTTACGA	41<br>i7D05<br>TCGGATTA	42<br>i7D06<br>TTGGTCTA	43<br>i7D07<br>ATAGCGGA	44<br>i7D08<br>TGTAACCA	45<br>i7D09<br>ACAGGCTA	46<br>i7D10<br>AAGTCGCA	47<br>i7D11<br>GAGTTCGA	48<br>i7D12<br>ACTCTTCA
**E**	49<br>i7E01<br>AAGACCTA	50<br>i7E02<br>ACCATCCA	51<br>i7E03<br>GAACGGTA	52<br>i7E04<br>TAGTGAGA	53<br>i7E05<br>GTCCAACA	54<br>i7E06<br>TATGGCGA	55<br>i7E07<br>GCTTATCA	56<br>i7E08<br>GTGACTGA	57<br>i7E09<br>ACGTGATA	58<br>i7E10<br>GGTTCTTA	59<br>i7E11<br>GATCAGCA	60<br>i7E12<br>GGACAATA
**F**	61<br>i7F01<br>AACTCTGA	62<br>i7F02<br>TGACCTTA	63<br>i7F03<br>GGAAGTGA	64<br>i7F04<br>AAGAAGGA	65<br>i7F05<br>GCAATCTA	66<br>i7F06<br>ACGAGTCA	67<br>i7F07<br>TTATGCCA	68<br>i7F08<br>GCCATAGA	69<br>i7F09<br>TAATCGGA	70<br>i7F10<br>TCTCGTTA	71<br>i7F11<br>GGTCTGTA	72<br>i7F12<br>ATCCGGTA
**G**	73<br>i7G01<br>ATGCGACA	74<br>i7G02<br>TGAACGCA	75<br>i7G03<br>TGGTATGA	76<br>i7G04<br>TGGACAGA	77<br>i7G05<br>TTGCAAGA	78<br>i7G06<br>ACTAGGTA	79<br>i7G07<br>TACCGATA	80<br>i7G08<br>ATTGGTGA	81<br>i7G09<br>TTCCTCGA	82<br>i7G10<br>TCATGGTA	83<br>i7G11<br>TATCCACA	84<br>i7G12<br>GACTTGTA
**H**	85<br>i7H01<br>ATGGAGTA	86<br>i7H02<br>GTCAGATA	87<br>i7H03<br>GAGCACTA	88<br>i7H04<br>GTCATTCA	89<br>i7H05<br>TCCTAAGA	90<br>i7H06<br>GATGCGTA	91<br>i7H07<br>TGCTTCCA	92<br>i7H08<br>ATGTCAGA	93<br>i7H09<br>GTTGCTCA	94<br>i7H10<br>GATATCCA	95<br>i7H11<br>GTTAGCGA	96<br>i7H12<br>GAGGTACA

	1	2	3	4	5	6	7	8	9	10	11	12
**A**	1<br>i5A01<br>GAGGTAGT	2<br>i5A02<br>GCTTAACT	3<br>i5A03<br>GCAATTCT	4<br>i5A04<br>TCCTCACT	5<br>i5A05<br>AGTTAGCT	6<br>i5A06<br>TCATGGCT	7<br>i5A07<br>AGCCTAAT	8<br>i5A08<br>AACACTCT	9<br>i5A09<br>AGAGCTCT	10<br>i5A10<br>GGCCATAT	11<br>i5A11<br>TAGAACGT	12<br>i5A12<br>GGTTGAAT
**B**	13<br>i5B01<br>GATCCTAT	14<br>i5B02<br>TGGTCTCT	15<br>i5B03<br>TCCAGTCT	16<br>i5B04<br>TAGCGTAT	17<br>i5B05<br>GCGATCAT	18<br>i5B06<br>AGCTACAT	19<br>i5B07<br>ACTCATCT	20<br>i5B08<br>TTGCGAGT	21<br>i5B09<br>AAGCGACT	22<br>i5B10<br>TGCACGAT	23<br>i5B11<br>TATGCGGT	24<br>i5B12<br>TCGTCGAT
**C**	25<br>i5C01<br>ACACTTGT	26<br>i5C02<br>TACGGCAT	27<br>i5C03<br>TCTCGTGT	28<br>i5C04<br>GCAGATGT	29<br>i5C05<br>TCCGTTGT	30<br>i5C06<br>TTCGCAGT	31<br>i5C07<br>GGAATAGT	32<br>i5C08<br>GCAACAAT	33<br>i5C09<br>ACTGCGAT	34<br>i5C10<br>TGAGTACT	35<br>i5C11<br>ACACGAAT	36<br>i5C12<br>GTGGCAAT
**D**	37<br>i5D01<br>GTCATCCT	38<br>i5D02<br>ACCGTACT	39<br>i5D03<br>GTACTACT	40<br>i5D04<br>TAGGTCCT	41<br>i5D05<br>GTTACTGT	42<br>i5D06<br>GCCTTAGT	43<br>i5D07<br>AACCATGT	44<br>i5D08<br>GATGACAT	45<br>i5D09<br>AGAAGACT	46<br>i5D10<br>TCAAGCAT	47<br>i5D11<br>TGGATGCT	48<br>i5D12<br>ACTTGCCT
**E**	49<br>i5E01<br>GTTGTGAT	50<br>i5E02<br>TATCTCGT	51<br>i5E03<br>TGTAAGGT	52<br>i5E04<br>ACGTTGGT	53<br>i5E05<br>AGGAGGAT	54<br>i5E06<br>GAGCAGAT	55<br>i5E07<br>TCTCTACT	56<br>i5E08<br>GGATTCAT	57<br>i5E09<br>GACCTTCT	58<br>i5E10<br>ACGGTTAT	59<br>i5E11<br>AGATGCGT	60<br>i5E12<br>TGACACCT
**F**	61<br>i5F01<br>ACTAACGT	62<br>i5F02<br>GAATGCCT	63<br>i5F03<br>GTGTACGT	64<br>i5F04<br>AAGTCAGT	65<br>i5F05<br>TTCCTGGT	66<br>i5F06<br>ACCACCAT	67<br>i5F07<br>GAGTCCAT	68<br>i5F08<br>AGCTCTGT	69<br>i5F09<br>GGTCTTGT	70<br>i5F10<br>AATGTGCT	71<br>i5F11<br>GTCTGGAT	72<br>i5F12<br>GCGTATAT
**G**	73<br>i5G01<br>TGACTGAT	74<br>i5G02<br>TGTCCAGT	75<br>i5G03<br>TTGTGCCT	76<br>i5G04<br>ACAGACCT	77<br>i5G05<br>GATCAAGT	78<br>i5G06<br>GATAGGAT	79<br>i5G07<br>GTAGTCGT	80<br>i5G08<br>TCACCTAT	81<br>i5G09<br>AGTGGTAT	82<br>i5G10<br>GCTAGAGT	83<br>i5G11<br>TTAACGCT	84<br>i5G12<br>TCAGGAGT
**H**	85<br>i5H01<br>AACTGGCT	86<br>i5H02<br>TACAGAGT	87<br>i5H03<br>TTGCATCT	88<br>i5H04<br>GAACGTGT	89<br>i5H05<br>AAGTACCT	90<br>i5H06<br>GGTACCAT	91<br>i5H07<br>AATCGCAT	92<br>i5H08<br>GGTAATCT	93<br>i5H09<br>GATTCGCT	94<br>i5H10<br>AGCATCGT	95<br>i5H11<br>GTTCACCT	96<br>i5H12<br>AGGCAAGT

Workflow B: i5 'reverse complement', i7 'reverse complement'

	1	2	3	4	5	6	7	8	9	10	11	12
A	1<br>i7A01<br>GTACGTCA	2<br>i7A02<br>TGCAGTTA	3<br>i7A03<br>ACTGTGGA	4<br>i7A04<br>GGTTAAGA	5<br>i7A05<br>TCACACTA	6<br>i7A06<br>TAGAGGTA	7<br>i7A07<br>GCGACATA	8<br>i7A08<br>TACATGCA	9<br>i7A09<br>GATGATGA	10<br>i7A10<br>TGTGTGCA	11<br>i7A11<br>TCGCTACA	12<br>i7A12<br>AAGCTAGA
B	13<br>i7B01<br>TAGGACCA	14<br>i7B02<br>TCGTTGGA	15<br>i7B03<br>GTGTCCTA	16<br>i7B04<br>TCCGTATA	17<br>i7B05<br>TACGCTTA	18<br>i7B06<br>TACAACGA	19<br>i7B07<br>GAAGGAGA	20<br>i7B08<br>AAGCATCA	21<br>i7B09<br>TGAGATCA	22<br>i7B10<br>TCTAGACA	23<br>i7B11<br>TGCTCATA	24<br>i7B12<br>TAACTCCA
C	25<br>i7C01<br>GACACACA	26<br>i7C02<br>TTGTAGCA	27<br>i7C03<br>GTCTACGA	28<br>i7C04<br>GCATTACA	29<br>i7C05<br>GTTCCATA	30<br>i7C06<br>GAATACCA	31<br>i7C07<br>TCACTTGA	32<br>i7C08<br>ACACAAGA	33<br>i7C09<br>ACTACAGA	34<br>i7C10<br>TCTCAGGA	35<br>i7C11<br>ATCGTGCA	36<br>i7C12<br>TCCACGTA
D	37<br>i7D01<br>ATCCATGA	38<br>i7D02<br>TTCGCAGA	39<br>i7D03<br>TCGTCTCA	40<br>i7D04<br>ACTTACGA	41<br>i7D05<br>TCGGATTA	42<br>i7D06<br>TTGGTCTA	43<br>i7D07<br>ATAGCGGA	44<br>i7D08<br>TGTAACCA	45<br>i7D09<br>ACAGGCTA	46<br>i7D10<br>AAGTCGCA	47<br>i7D11<br>GAGTTCGA	48<br>i7D12<br>ACTCTTCA
E	49<br>i7E01<br>AAGACCTA	50<br>i7E02<br>ACCATCCA	51<br>i7E03<br>GAACGGTA	52<br>i7E04<br>TAGTGAGA	53<br>i7E05<br>GTCCAACA	54<br>i7E06<br>TATGGCGA	55<br>i7E07<br>GCTTATCA	56<br>i7E08<br>GTGACTGA	57<br>i7E09<br>ACGTGATA	58<br>i7E10<br>GGTTCTTA	59<br>i7E11<br>GATCAGCA	60<br>i7E12<br>GGACAATA
F	61<br>i7F01<br>AACTCTGA	62<br>i7F02<br>TGACCTTA	63<br>i7F03<br>GGAAGTGA	64<br>i7F04<br>AAGAAGGA	65<br>i7F05<br>GCAATCTA	66<br>i7F06<br>ACGAGTCA	67<br>i7F07<br>TTATGCCA	68<br>i7F08<br>GCCATAGA	69<br>i7F09<br>TAATCGGA	70<br>i7F10<br>TCTCGTTA	71<br>i7F11<br>GGTCTGTA	72<br>i7F12<br>ATCCGGTA
G	73<br>i7G01<br>ATGCGACA	74<br>i7G02<br>TGAACGCA	75<br>i7G03<br>TGGTATGA	76<br>i7G04<br>TGGACAGA	77<br>i7G05<br>TTGCAAGA	78<br>i7G06<br>ACTAGGTA	79<br>i7G07<br>TACCGATA	80<br>i7G08<br>ATTGGTGA	81<br>i7G09<br>TTCCTCGA	82<br>i7G10<br>TCATGGTA	83<br>i7G11<br>TATCCACA	84<br>i7G12<br>GACTTGTA
H	85<br>i7H01<br>ATGGAGTA	86<br>i7H02<br>GTCAGATA	87<br>i7H03<br>GAGCACTA	88<br>i7H04<br>GTCATTCA	89<br>i7H05<br>TCCTAAGA	90<br>i7H06<br>GATGCGTA	91<br>i7H07<br>TGCTTCCA	92<br>i7H08<br>ATGTCAGA	93<br>i7H09<br>GTTGCTCA	94<br>i7H10<br>GATATCCA	95<br>i7H11<br>GTTAGCGA	96<br>i7H12<br>GAGGTACA

	1	2	3	4	5	6	7	8	9	10	11	12
A	1<br>i5A01<br>CTCCATCA	2<br>i5A02<br>CGAATTGA	3<br>i5A03<br>CGTTAAGA	4<br>i5A04<br>AGGAGTGA	5<br>i5A05<br>TCAATCGA	6<br>i5A06<br>AGTACCGA	7<br>i5A07<br>TCGGATTA	8<br>i5A08<br>TTGTGAGA	9<br>i5A09<br>TCTCGAGA	10<br>i5A10<br>CCGGTATA	11<br>i5A11<br>ATCTTGCA	12<br>i5A12<br>CCAACTTA
B	13<br>i5B01<br>CTAGGATA	14<br>i5B02<br>ACCAGAGA	15<br>i5B03<br>AGGTCAGA	16<br>i5B04<br>ATCGCATA	17<br>i5B05<br>CGCTAGTA	18<br>i5B06<br>TCGATGTA	19<br>i5B07<br>TGAGTAGA	20<br>i5B08<br>AACGCTCA	21<br>i5B09<br>TTCGCTGA	22<br>i5B10<br>ACGTGCTA	23<br>i5B11<br>ATACGCCA	24<br>i5B12<br>AGCAGCTA
C	25<br>i5C01<br>TGTGAACA	26<br>i5C02<br>ATGCCGTA	27<br>i5C03<br>AGAGCACA	28<br>i5C04<br>CGTCTACA	29<br>i5C05<br>AGGCAACA	30<br>i5C06<br>AAGCGTCA	31<br>i5C07<br>CCTTATCA	32<br>i5C08<br>CGTTGTTA	33<br>i5C09<br>TGACGCTA	34<br>i5C10<br>ACTCATGA	35<br>i5C11<br>TGTGCTTA	36<br>i5C12<br>CACCGTTA
D	37<br>i5D01<br>CAGTAGGA	38<br>i5D02<br>TGGCATGA	39<br>i5D03<br>CATGATGA	40<br>i5D04<br>ATCCAGGA	41<br>i5D05<br>CAATGACA	42 Text<br>i5D06<br>CGGAATCA	43<br>i5D07<br>TTGGTACA	44<br>i5D08<br>CTACTGTA	45<br>i5D09<br>TCTTCTGA	46<br>i5D10<br>AGTTCGTA	47<br>i5D11<br>ACCTACGA	48<br>i5D12<br>TGAACGGA
E	49<br>i5E01<br>CAACACTA	50<br>i5E02<br>ATAGAGCA	51<br>i5E03<br>ACATTCCA	52<br>i5E04<br>TGCAACCA	53<br>i5E05<br>TCCTCCTA	54<br>i5E06<br>CTCGTCTA	55<br>i5E07<br>AGAGATGA	56<br>i5E08<br>CCTAAGTA	57<br>i5E09<br>CTGGAAGA	58<br>i5E10<br>TGCCAATA	59<br>i5E11<br>TCTACGCA	60<br>i5E12<br>ACTGTGGA
F	61<br>i5F01<br>TGATTGCA	62<br>i5F02<br>CTTACGGA	63<br>i5F03<br>CACATGCA	64<br>i5F04<br>TTCAGTCA	65<br>i5F05<br>AAGGACCA	66<br>i5F06<br>TGGTGGTA	67<br>i5F07<br>CTCAGGTA	68<br>i5F08<br>TCGAGACA	69<br>i5F09<br>CCAGAACA	70<br>i5F10<br>TTACACGA	71<br>i5F11<br>CAGACCTA	72<br>i5F12<br>CGCATATA
G	73<br>i5G01<br>ACTGACTA	74<br>i5G02<br>ACAGGTCA	75<br>i5G03<br>AACACGGA	76<br>i5G04<br>TGTCTGGA	77<br>i5G05<br>CTAGTTCA	78<br>i5G06<br>CTATCCTA	79<br>i5G07<br>CATCAGCA	80<br>i5G08<br>AGTGGATA	81<br>i5G09<br>TCACCATA	82<br>i5G10<br>CGATCTCA	83<br>i5G11<br>AATTGCGA	84<br>i5G12<br>AGTCCTCA
H	85<br>i5H01<br>TTGACCGA	86<br>i5H02<br>ATGTCTCA	87<br>i5H03<br>AACGTAGA	88<br>i5H04<br>CTTGCACA	89<br>i5H05<br>TTCATGGA	90<br>i5H06<br>CCATGGTA	91<br>i5H07<br>TTAGCGTA	92<br>i5H08<br>CCATTAGA	93<br>i5H09<br>CTAAGCGA	94<br>i5H10<br>TCGTAGCA	95<br>i5H11<br>CAAGTGGA	96<br>i5H12<br>TCCGTTCA

Supplementary Figure S7. Procedure for automated delivery of i7 and i5 primers to 384-well plates for PCR2, using the Echo® liquid handler (Labcyte, San Jose, CA / Beckman Coulter). The format illustrated preserves barcode relationships assigned to samples in 96-well format, and as elaborated in an Illumina® Sample Sheet using SampleSheet.py.

(a) Index oligo stocks can be transferred from 96-well source plates (typical source format from oligo supplier) to 384-well plates and adjusted to a practical stock concentration, using a 12-channel pipet. In this example, i5 oligos are arrayed in rows 1-4 of a Labcyte Echo®-qualified 384-well polypropylene source plate (well IDs and numbers correspond to ID in 96-well plate), and i7 oligos are arrayed in rows 9-12 (in up to 65 μL volumes, plates sealed with adhesive foil and stored at -20 °C). Preparing the index oligos in 384-well format facilitates convenient, automated transfer of oligos to destination wells as defined in Labcyte Plate Reformat software;

(b) Example workflow for PCR1 & PCR2 setup in 384-well plates. Here, four source plates (Plates 1-4) are indicated. 1) *Master Mix* for PCR1 is delivered using a multi-channel pipet to wells of a 384-well PCR plate (*labeled #1*); 2) Appropriate volume of sample lysate (genomic DNA source) is transferred from 96-well source plates to wells of a 384-well plate, enabling content from four 96-well plates to be arrayed into a single 384-well plate to expedite processing; 3) in a separate 384-well PCR plate (*labeled #2*), PCR2 Master Mix can be delivered by hand (12-channel pipet) or by Echo (*e.g.,* from 6-reservoir source plate); 4) *i7 and i5 indices can be delivered in small volumes to target wells using Echo acoustic technology (i5 across individual groups of four rows that correspond to a single 96-well plate; i7 to individual wells within a block of four rows)*; 5) finally, PCR2 template (small volume of PCR1 template) can be delivered by hand (12-channel pipet) from PCR1 plate to PCR2 plate. For those with access to other automation tools, such as Biomek (Beckman Coulter) or Apricot Designs liquid handlers, robotic-assisted plate setups could be used to accomplish any manual (multi-channel pipet) step illustrated here.

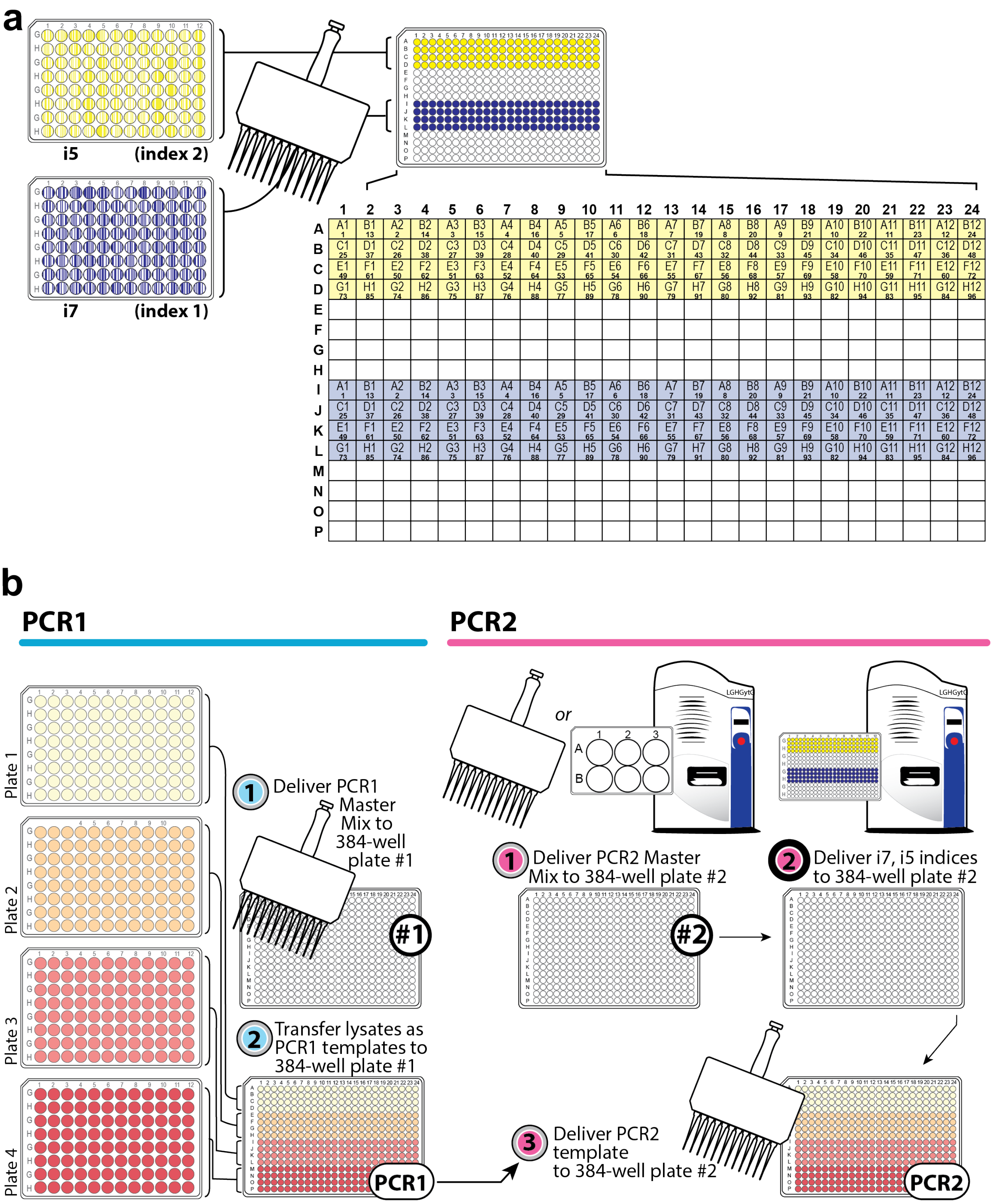
a
i5
(index 2)
i7
(index 1)
b
PCR1
PCR2
Plate 1
Plate 2
Plate 3
Plate 4
1 Deliver PCR1 Master Mix to 384-well plate #1
#1
2 Transfer lysates as PCR1 templates to 384-well plate #1
PCR1
or
1 Deliver PCR2 Master Mix to 384-well plate #2
#2
2 Deliver i7, i5 indices to 384-well plate #2
3 Deliver PCR2 template to 384-well plate #2
PCR2

Supplementary Figure S8. Amplicon length considerations in library preparation: PCR product purification and sequencing recovery.

Some editing cases may present situations where amplicons of varied length are pooled as a common library for sequencing; we empirically tested amplicon length-dependent recovery of reads to assess length-dependent recovery of short read sequences on an Illumina® platform.

(a) ***Purification of PCR products.*** In library preparation, SPRI clean-up retains longer amplicons more effectively than column clean-up. Four independent PCR products (labeled *red* 1-4, ranging from ~358-801 bp) were pooled, purified either by SPRISelect (Beckman Coulter, Brea, CA) or DNA Clean & Concentrator-5 (Zymo Research, Irvine, CA), and analyzed by Bioanalyzer 2100 (High Sensitivity DNA Assay, Agilent). Shown are traces (*left*), gel-like image and expected sizes of wild type PCR amplicons without adaptors (*right*).

(b) ***Amplicon length-dependent sequence recovery on MiSeq.*** To test the amplicon lengths most readily compatible with Illumina® MiSeq technology, we prepared a library of seven amplicons ranging in lengths from 100 – 1500 bp, identical in sequence at their ends outside of internal extensions for longer amplicons. Amplicons were uniquely barcoded such that they could be pooled in separate library sets, defined by molar ratios specified as 1:1:1:1:1:1:1 or 1:10:50:100:200:500:1000 (relative to 100:175:300:500:800:1000:1500 bp amplicons) (*see* **Supp. Methods**).

> *(i)* among total mapped reads, amplicon size >500 bp was associated with representation below expected (expected ~14.3% for each amplicon in 1:1 ratio [*horizontal dashed line*, upper left plot], *vertical black lines*, lower left plot); *insets*: absolute mapped read counts relative to amplicon size (bp);
>
> *(ii)* among top 15 most abundant reads identified for each amplicon in each library ratio, the % of R1 & R2 reads that mapped to each amplicon reference sequence appeared relatively consistent for amplicons up to 800 bp, and was markedly blunted for an amplicon 1500 bp in length. This suggested to us that amplicons up to 800 bp generally perform well for clustering and sequencing, but that performance drops for amplicons beyond these lengths (pronounced for 1500 bp).

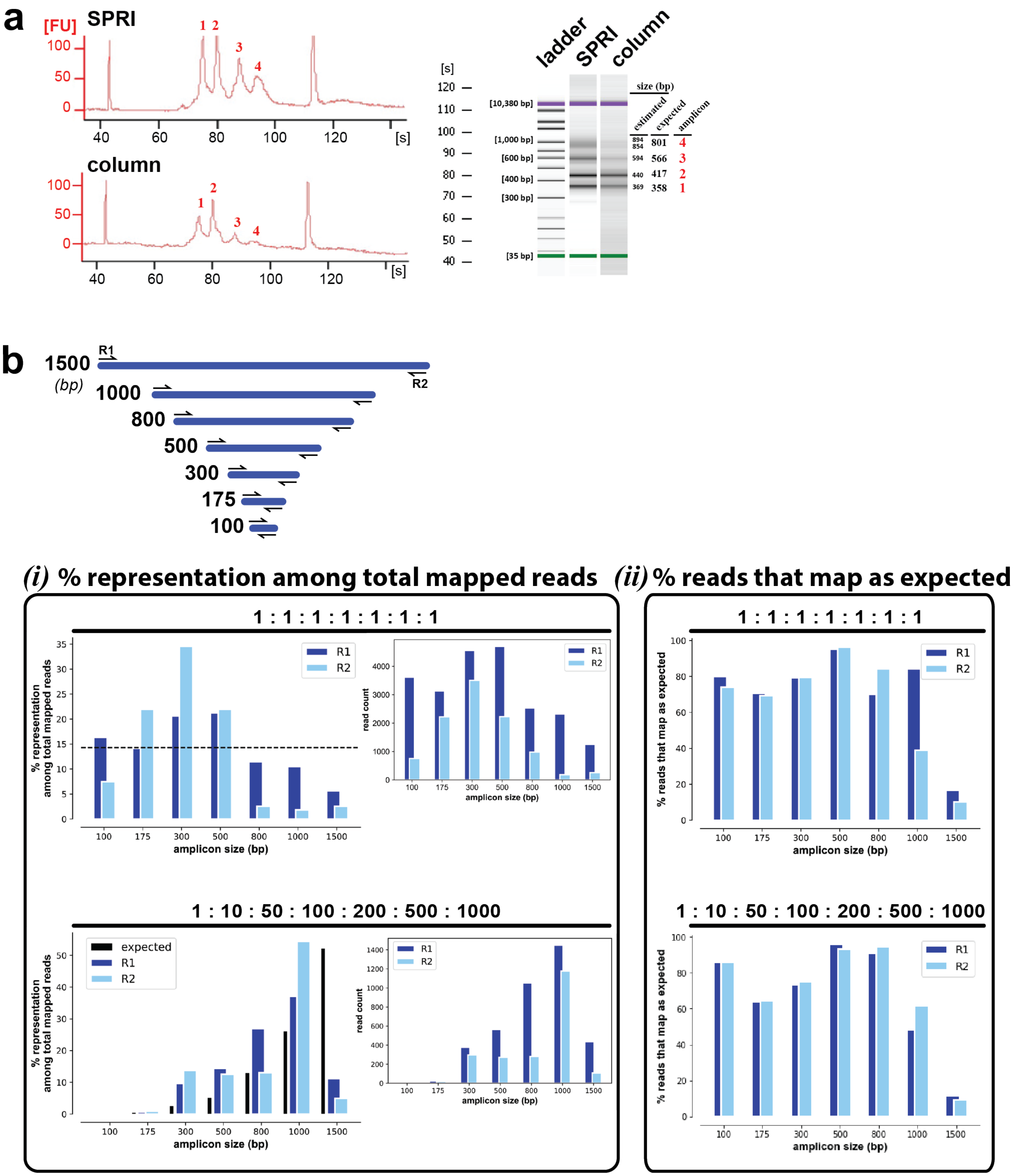
a
SPRI
column
ladder
size (bp)
estimated
expected
amplicon
b
1500
(bp)
1000
800
500
300
175
100
R1
R2
(i) % representation among total mapped reads
(ii) % reads that map as expected
1 : 1 : 1 : 1 : 1 : 1 : 1
1 : 10 : 50 : 100 : 200 : 500 : 1000
% representation among total mapped reads
read count
% reads that map as expected
amplicon size (bp)
expected

Supplementary Figure S9. File outputs of Genotypes.py.

Example output files are available in the **ExampleOutputFiles** directory of the associated Zenodo repository (**DOI 10.5281/zenodo.3406861**).

(a) Example of custom-named directory (Genotypes.py user *input* #1) populated with 8 output files; filenames are automatically prefixed by script operations with system start date. ***fasta.fa*** and ***blastn_alignments.txt*** are prepared during script operations, but key script output content is contained in the other six files.

GenotypesOutput
- 01032021_allele_definitions.csv
- 01032021_allele_definitions.txt
- 01032021_allele_evidence.pdf
- 01032021_blastn_alignments.txt
- 01032021_fasta.fa
- 01032021_genotypes.txt
- 01032021_population_summary.txt
- 01032021_script_metrics.txt

(b) ***allele_definitions.csv*** provides comma-separated panel data compatible with spreadsheet programs such as Excel, and/or import as panel data in Python pandas for user access and manipulation. This file contains raw content of *genotypes.dict,* the primary Python data object of Genotypes.py (sample-specific Ranked Alleles and their inferred properties). This spreadsheet does not contain deprecated Ranked Alleles, which are logged only in population_summary.txt.

	A	B	C	D	E	F	G	H	I	J	K	L	M	N	O	P	Q	R	S	T	U
1		allele	read	sample	reads	totalreads	%totalreads	%top10reads	%readsfilteredfor>1%	%readsfilteredfor>10%	chr	locusID	coordinates	alignment_query	alignment_midline	alignment_hit	allele_type	allele_specs	inferred_genotype		
3190	3188	KE4-4-G01_2	R1+R2	KE4-4-G01	367	881	41.66	47.48	48.87	49.8	Homo sapier	ref|NC_000006.12|	35602016-35602190	query ACTTAAA	||||||	reference ACTT	mutant	likely deletion, 4 bp	|heterozygous| deletion1 + deletion2 (del1/del2)		
3191	3189	KE4-4-G01_3	R1+R2	KE4-4-G01	14	881	1.59	1.81	1.86	None	Homo sapier	ref|NC_000006.12|	35602016-35602190	query ACTTAAA	||||||	reference ACTT	mutant	likely deletion, 3 bp	|heterozygous| deletion1 + deletion2 (del1/del2)		
3192	3190	KE4-4-G01_4	R1+R2	KE4-4-G01	7	881	0.79	0.91	None	None	Homo sapier	ref|NC_000006.12|	35602016-35602190	query ACTTAAA	||||||	reference ACTT	mutant	likely deletion, 5 bp	|heterozygous| deletion1 + deletion2 (del1/del2)		
3193	3191	KE4-4-G01_5	R1+R2	KE4-4-G01	4	881	0.45	0.52	None	None	Homo sapier	ref|NC_000006.12|	35602190-35602016	query TTACTGC	||||||	reference TTAC	mutant	likely deletion, 4 bp	|heterozygous| deletion1 + deletion2 (del1/del2)		
3194	3192	KE4-4-G01_6	R1+R2	KE4-4-G01	3	881	0.34	0.39	None	None	Homo sapier	ref|NC_000006.12|	35602018-35602190	query TTAAACT	||||||	reference TTAA	mutant	likely deletion, 2 bp	|heterozygous| deletion1 + deletion2 (del1/del2)		
3195	3193	KE4-4-G01_7	R1+R2	KE4-4-G01	2	881	0.23	0.26	None	None	Homo sapier	ref|NC_000006.12|	35602016-35602190	query ACTTAAA	||||||	reference ACTT	mutant	likely deletion, 4 bp	|heterozygous| deletion1 + deletion2 (del1/del2)		
3196	3194	KE4-4-G01_8	R1+R2	KE4-4-G01	2	881	0.23	0.26	None	None	Homo sapier	ref|NC_000006.12|	35602016-35602190	query ACTTAAA	||||||	reference ACTT	wild-type		|heterozygous| deletion1 + deletion2 (del1/del2)		
3197	3195	KE4-4-G01_9	R1+R2	KE4-4-G01	2	881	0.23	0.26	None	None	Homo sapier	ref|NC_000006.12|	35602016-35602190	query ACTTAAA	||||||	reference ACTT	mutant	likely deletion, 3 bp	|heterozygous| deletion1 + deletion2 (del1/del2)		
3198	3196	KE4-4-G01_10	R1+R2	KE4-4-G01	2	881	0.23	0.26	None	None	Homo sapier	ref|NC_000006.12|	35602016-35602190	query ACTTAAA	||||||	reference ACTT	mutant	likely deletion, 4 bp	|heterozygous| deletion1 + deletion2 (del1/del2)		
3199	3197	KE4-4-G02_1	R1+R2	KE4-4-G02	443	996	44.48	51.75	53.31	54.89	Homo sapier	ref|NC_000006.12|	35602016-35602190	query ACTTAAA	||||||	reference ACTT	mutant	likely deletion, 4 bp	|heterozygous| deletion1 + deletion2 (del1/del2)		
3200	3198	KE4-4-G02_2	R1+R2	KE4-4-G02	364	996	36.55	42.52	43.8	45.11	Homo sapier	ref|NC_000006.12|	35602016-35602190	query ACTTAAA	||||||	reference ACTT	mutant	likely deletion, 2 bp	|heterozygous| deletion1 + deletion2 (del1/del2)		
3201	3199	KE4-4-G02_3	R1+R2	KE4-4-G02	13	996	1.31	1.52	1.56	None	Homo sapier	ref|NC_000006.12|	35602016-35602190	query ACTTAAA	||||||	reference ACTT	mutant	likely deletion, 3 bp	|heterozygous| deletion1 + deletion2 (del1/del2)		
3202	3200	KE4-4-G02_4	R1+R2	KE4-4-G02	11	996	1.1	1.29	1.32	None	Homo sapier	ref|NC_000006.12|	35602016-35602190	query ACTTAAA	||||||	reference ACTT	mutant	likely deletion, 5 bp	|heterozygous| deletion1 + deletion2 (del1/del2)		
3203	3201	KE4-4-G02_5	R1+R2	KE4-4-G02	7	996	0.7	0.82	None	None	Homo sapier	ref|NC_000006.12|	35602016-35602190	query ACTTAAA	||||||	reference ACTT	wild-type		|heterozygous| deletion1 + deletion2 (del1/del2)		
3204	3202	KE4-4-G02_6	R1+R2	KE4-4-G02	5	996	0.5	0.58	None	None	Homo sapier	ref|NC_000006.12|	35602190-35602016	query TTACTGC	||||||	reference TTAC	mutant	likely deletion, 2 bp	|heterozygous| deletion1 + deletion2 (del1/del2)		
3205	3203	KE4-4-G02_7	R1+R2	KE4-4-G02	4	996	0.4	0.47	None	None	Homo sapier	ref|NC_000006.12|	35602016-35602190	query ACTTAAA	||||||	reference ACTT	mutant	likely deletion, 5 bp	|heterozygous| deletion1 + deletion2 (del1/del2)		
3206	3204	KE4-4-G02_8	R1+R2	KE4-4-G02	3	996	0.3	0.35	None	None	Homo sapier	ref|NC_000006.12|	35602016-35602190	query ACTTAAA	||||||	reference ACTT	mutant	likely deletion, 5 bp	|heterozygous| deletion1 + deletion2 (del1/del2)		
3207	3205	KE4-4-G02_9	R1+R2	KE4-4-G02	3	996	0.3	0.35	None	None	Homo sapier	ref|NC_000006.12|	35602016-35602190	query ACTTAAA	||||||	reference ACTT	mutant	likely deletion, 2 bp	|heterozygous| deletion1 + deletion2 (del1/del2)		
3208	3206	KE4-4-G02_10	R1+R2	KE4-4-G02	3	996	0.3	0.35	None	None	Homo sapier	ref|NC_000006.12|	35602016-35602190	query ACTTAAA	||||||	reference ACTT	mutant	likely deletion, 3 bp	|heterozygous| deletion1 + deletion2 (del1/del2)		
3209	3207	KE4-4-G03_1	R1+R2	KE4-4-G03	712	832	85.58	94.93	97.4	100	Homo sapier	ref|NC_000006.12|	35602016-35602190	query ACTTAAA	||||||	reference ACTT	wild-type		|homozygous| wild-type (wt/wt)		
3210	3208	KE4-4-G03_2	R1+R2	KE4-4-G03	19	832	2.28	2.53	2.6	None	Homo sapier	ref|NC_000006.12|	35602016-35602190	query ACTTAAA	||||||	reference ACTT	mutant	likely deletion, 1 bp	|homozygous| wild-type (wt/wt)		
3211	3209	KE4-4-G03_3	R1+R2	KE4-4-G03	3	832	0.36	0.4	None	None	Homo sapier	ref|NC_000006.12|	35602016-35602190	query ACTTAAA	||||||	reference ACTT	mutant	likely deletion, 1 bp	|homozygous| wild-type (wt/wt)		
3212	3210	KE4-4-G03_4	R1+R2	KE4-4-G03	3	832	0.36	0.4	None	None	Homo sapier	ref|NC_000006.12|	35602016-35602190	query ACTTAAA	||||||	reference ACTT	mutant	likely substitution	|homozygous| wild-type (wt/wt)		
3213	3211	KE4-4-G03_5	R1+R2	KE4-4-G03	3	832	0.36	0.4	None	None	Homo sapier	ref|NC_000006.12|	35602018-35602190	query TTAAACT	||||||	reference TTAA	wild-type		|homozygous| wild-type (wt/wt)		
3214	3212	KE4-4-G03_6	R1+R2	KE4-4-G03	2	832	0.24	0.27	None	None	Homo sapier	ref|NC_000006.12|	35602016-35602190	query ACTTAAA	||||||	reference ACTT	mutant	likely substitution	|homozygous| wild-type (wt/wt)		
3215	3213	KE4-4-G03_7	R1+R2	KE4-4-G03	2	832	0.24	0.27	None	None	Homo sapier	ref|NC_000006.12|	35602016-35602190	query ACTTAAA	||||||	reference ACTT	mutant	likely substitution	|homozygous| wild-type (wt/wt)		
3216	3214	KE4-4-G03_8	R1+R2	KE4-4-G03	2	832	0.24	0.27	None	None	Homo sapier	ref|NC_000006.12|	35602016-35602189	query ACTTAAA	||||||	reference ACTT	wild-type		|homozygous| wild-type (wt/wt)		
3217	3215	KE4-4-G03_9	R1+R2	KE4-4-G03	2	832	0.24	0.27	None	None	Homo sapier	ref|NC_000006.12|	35602016-35602190	query ACTTAAA	||||||	reference ACTT	mutant	likely substitution	|homozygous| wild-type (wt/wt)		
3218	3216	KE4-4-G03_10	R1+R2	KE4-4-G03	2	832	0.24	0.27	None	None	Homo sapier	ref|NC_000006.12|	35602016-35602190	query ACTTAAA	||||||	reference ACTT	mutant	likely substitution	|homozygous| wild-type (wt/wt)		
3219	3217	KE4-4-G04_1	R1+R2	KE4-4-G04	1	1	100	100	100	100	Homo sapier	ref|NC_000006.12|	35602016-35602190	query ACTTAAA	||||||	reference ACTT	wild-type		|homozygous| wild-type (wt/wt)		
3220	3218	KE4-4-G05_1	R1+R2	KE4-4-G05	643	770	83.51	93.46	96.26	100	Homo sapier	ref|NC_000006.12|	35602016-35602190	query ACTTAAA	||||||	reference ACTT	wild-type		|homozygous| wild-type (wt/wt)		
3221	3219	KE4-4-G05_2	R1+R2	KE4-4-G05	17	770	2.21	2.47	2.54	None	Homo sapier	ref|NC_000006.12|	35602016-35602190	query ACTTAAA	||||||	reference ACTT	mutant	likely deletion, 1 bp	|homozygous| wild-type (wt/wt)		
3222	3220	KE4-4-G05_3	R1+R2	KE4-4-G05	8	770	1.04	1.16	1.2	None	Homo sapier	ref|NC_000006.12|	35602190-35602016	query TTACTGC	||||||	reference TTAC	wild-type		|homozygous| wild-type (wt/wt)		
3223	3221	KE4-4-G05_4	R1+R2	KE4-4-G05	6	770	0.78	0.87	None	None	Homo sapier	ref|NC_000006.12|	35602016-35602189	query ACTTAAA	||||||	reference ACTT	wild-type		|homozygous| wild-type (wt/wt)		
3224	3222	KE4-4-G05_5	R1+R2	KE4-4-G05	3	770	0.39	0.44	None	None	Homo sapier	ref|NC_000006.12|	35602016-35602190	query ACTTAAA	||||||	reference ACTT	mutant	likely deletion, 1 bp	|homozygous| wild-type (wt/wt)		
3225	3223	KE4-4-G05_6	R1+R2	KE4-4-G05	3	770	0.39	0.44	None	None	Homo sapier	ref|NC_000006.12|	35602016-35602190	query ACTTAAA	||||||	reference ACTT	mutant	likely deletion, 1 bp	|homozygous| wild-type (wt/wt)		
3226	3224	KE4-4-G05_7	R1+R2	KE4-4-G05	2	770	0.26	0.29	None	None	Homo sapier	ref|NC_000006.12|	35602016-35602190	query ACTTAAA	||||||	reference ACTT	mutant	likely substitution	|homozygous| wild-type (wt/wt)		
3227	3225	KE4-4-G05_8	R1+R2	KE4-4-G05	2	770	0.26	0.29	None	None	Homo sapier	ref|NC_000006.12|	35602016-35602190	query ACTTAAA	||||||	reference ACTT	mutant	likely substitution	|homozygous| wild-type (wt/wt)		
3228	3226	KE4-4-G05_9	R1+R2	KE4-4-G05	2	770	0.26	0.29	None	None	Homo sapier	ref|NC_000006.12|	35602016-35602190	query ACTTAAA	||||||	reference ACTT	mutant	likely substitution	|homozygous| wild-type (wt/wt)		
3229	3227	KE4-4-G05_10	R1+R2	KE4-4-G05	2	770	0.26	0.29	None	None	Homo sapier	ref|NC_000006.12|	35602016-35602190	query ACTTAAA	||||||	reference ACTT	mutant	likely substitution	|homozygous| wild-type (wt/wt)		
3230	3228	KE4-4-G06_1	R1+R2	KE4-4-G06	674	809	83.31	93.87	96.84	100	Homo sapier	ref|NC_000006.12|	35602016-35602190	query ACTTAAA	||||||	reference ACTT	wild-type		|homozygous| wild-type (wt/wt)		

01032021_allele_definitions

(c) ***allele_definitions.txt*** reports (up to) the top 10 ranked alleles for R1+R2 (paired-end/PE data), with allele metadata (frequency metrics, allele specifications, reference sequence database alignment location and alignment), along with (optional) mapped DNA sub-sequences (*e.g.*, Cas9 guide RNA sequence(s), test sequence(s). Hypothesized alleles derive from two sources in Genotypes.py: 1) direct BLASTN alignments, and 2) reconstructions for high-scoring alignment pairs (hsp's) reported separately by BLASTN but recovered by Genotypes.py for reconstituted alignment (using BLASTDBCMD to retrieve reference sequenced spanned by the hsp's).

genotypes.txt reports the same content, but ordered based on inferred genotype cohorts. Predicted Cas9 incision site is denoted by "v" under "guide sequence".

```
=========
KE4-4-G02
=========
Inferred Genotype: |heterozygous| deletion1 + deletion2 (del1/del2)

   *~~~~~~~~~~~~~~~~*
   | READ 1 + READ 2 |
   *~~~~~~~~~~~~~~~~*

   Allele: KE4-4-G02 R1+R2 [443/996] rank1 %totalreads:44.48 percentile:100 %top10reads:51.75 %readsfilteredfor1%:53.31 %readsfilteredfor10%:54.89 | mutant, likely deletion, 4 bp
    Locus: Homo sapiens chromosome 6, GRCh38.p12 Primary Assembly, ref|NC_000006.12| 35602016-35602190

                                                                                    3'-CAAGACTTACACCGACCGTG-5' (guide sequence)
                                                                                          v
    query  ACTTAAACTGGAGCTCTGACTTATTGTTCTCTTACTGCCCTAGAGCAATTTTGTTTTGAAGAGCACAGAACACCCTGTT----ATGTGGCTGGCACATGAACTCGATGTGCTGACAGCAATTTGTACTCCGATTAAAATAGGGGGGAAAAAAGGAAAGAGAGTGCACAGCAGTAA
           |||||||||||||||||||||||||||||||||||||||||||||||||||||||||||||||||||||||||||||||    ||||||||||||||||||||||||||||||||||||||||||||||||||||||||||||||||||||||||||||||||||||||||||||
reference  ACTTAAACTGGAGCTCTGACTTATTGTTCTCTTACTGCCCTAGAGCAATTTTGTTTTGAAGAGCACAGAACACCCTGTTCTGAATGTGGCTGGCACATGAACTCGATGTGCTGACAGCAATTTGTACTCCGATTAAAATAGGGGGGAAAAAAGGAAAGAGAGTGCACAGCAGTAA
                                                                            ^^^^^^^^^^^^^^^^
                                                                         3'-GTCTTGTGGGACAAGA-5' (sequence of interest)


   Allele: KE4-4-G02 R1+R2 [364/996] rank2 %totalreads:36.55 percentile:99 %top10reads:42.52 %readsfilteredfor1%:43.8 %readsfilteredfor10%:45.11 | mutant, likely deletion, 2 bp
    Locus: Homo sapiens chromosome 6, GRCh38.p12 Primary Assembly, ref|NC_000006.12| 35602016-35602190

                                                                                    3'-CAAGACTTACACCGACCGTG-5' (guide sequence)
                                                                                          v
    query  ACTTAAACTGGAGCTCTGACTTATTGTTCTCTTACTGCCCTAGAGCAATTTTGTTTTGAAGAGCACAGAACACCCTG--CTGAATGTGGCTGGCACATGAACTCGATGTGCTGACAGCAATTTGTACTCCGATTAAAATAGGGGGGAAAAAAGGAAAGAGAGTGCACAGCAGTAA
           |||||||||||||||||||||||||||||||||||||||||||||||||||||||||||||||||||||||||||||  ||||||||||||||||||||||||||||||||||||||||||||||||||||||||||||||||||||||||||||||||||||||||||||||||||||
reference  ACTTAAACTGGAGCTCTGACTTATTGTTCTCTTACTGCCCTAGAGCAATTTTGTTTTGAAGAGCACAGAACACCCTGTTCTGAATGTGGCTGGCACATGAACTCGATGTGCTGACAGCAATTTGTACTCCGATTAAAATAGGGGGGAAAAAAGGAAAGAGAGTGCACAGCAGTAA
                                                                            ^^^^^^^^^^^^^^^^
                                                                         3'-GTCTTGTGGGACAAGA-5' (sequence of interest)


  *~~~~~~~~~~~~~~~~~~~~~~~~~~~~~~~~~~~~~~~~~~~~~~~~~~~~~~~*
  |  >>>>> remaining alleles occur at frequency <10% <<<<< |
  *~~~~~~~~~~~~~~~~~~~~~~~~~~~~~~~~~~~~~~~~~~~~~~~~~~~~~~~*

   Allele: KE4-4-G02 R1+R2 [13/996] rank3 %totalreads:1.31 percentile:98 %top10reads:1.52 %readsfilteredfor1%:1.56 %readsfilteredfor10%:None | mutant, likely deletion, 3 bp
    Locus: Homo sapiens chromosome 6, GRCh38.p12 Primary Assembly, ref|NC_000006.12| 35602016-35602190

                                                                                    3'-CAAGACTTACACCGACCGTG-5' (guide sequence)
                                                                                          v
    query  ACTTAAACTGGAGCTCTGAC-TATTGTTCTCTTACTGCCCTAGAGCAATTTTGTTTTGAAGAGCACAGAACACCCTG--CTGAATGTGGCTGGCACATGAACTCGATGTGCTGACAGCAATTTGTACTCCGATTAAAATAGGGGGGAAAAAAGGAAAGAGAGTGCACAGCAGTAA
           |||||||||||||||||||| ||||||||||||||||||||||||||||||||||||||||||||||||||||||||  ||||||||||||||||||||||||||||||||||||||||||||||||||||||||||||||||||||||||||||||||||||||||||||||||||||
reference  ACTTAAACTGGAGCTCTGACTTATTGTTCTCTTACTGCCCTAGAGCAATTTTGTTTTGAAGAGCACAGAACACCCTGTTCTGAATGTGGCTGGCACATGAACTCGATGTGCTGACAGCAATTTGTACTCCGATTAAAATAGGGGGGAAAAAAGGAAAGAGAGTGCACAGCAGTAA
                                                                            ^^^^^^^^^^^^^^^^
                                                                         3'-GTCTTGTGGGACAAGA-5' (sequence of interest)


   Allele: KE4-4-G02 R1+R2 [11/996] rank4 %totalreads:1.1 percentile:97 %top10reads:1.29 %readsfilteredfor1%:1.32 %readsfilteredfor10%:None | mutant, likely deletion, 5 bp
    Locus: Homo sapiens chromosome 6, GRCh38.p12 Primary Assembly, ref|NC_000006.12| 35602016-35602190

                                                                                    3'-CAAGACTTACACCGACCGTG-5' (guide sequence)
                                                                                          v
    query  ACTTAAACTGGAGCTCTGAC-TATTGTTCTCTTACTGCCCTAGAGCAATTTTGTTTTGAAGAGCACAGAACACCCTGTT----ATGTGGCTGGCACATGAACTCGATGTGCTGACAGCAATTTGTACTCCGATTAAAATAGGGGGGAAAAAAGGAAAGAGAGTGCACAGCAGTAA
           |||||||||||||||||||| ||||||||||||||||||||||||||||||||||||||||||||||||||||||||||    ||||||||||||||||||||||||||||||||||||||||||||||||||||||||||||||||||||||||||||||||||||||||||||
reference  ACTTAAACTGGAGCTCTGACTTATTGTTCTCTTACTGCCCTAGAGCAATTTTGTTTTGAAGAGCACAGAACACCCTGTTCTGAATGTGGCTGGCACATGAACTCGATGTGCTGACAGCAATTTGTACTCCGATTAAAATAGGGGGGAAAAAAGGAAAGAGAGTGCACAGCAGTAA
                                                                            ^^^^^^^^^^^^^^^^
                                                                         3'-GTCTTGTGGGACAAGA-5' (sequence of interest)


   Allele: KE4-4-G02 R1+R2 [7/996] rank5 %totalreads:0.7 percentile:97 %top10reads:0.82 %readsfilteredfor1%:None %readsfilteredfor10%:None | wild-type
    Locus: Homo sapiens chromosome 6, GRCh38.p12 Primary Assembly, ref|NC_000006.12| 35602016-35602190

    query  ACTTAAACTGGAGCTCTGACTTATTGTTCTCTTACTGCCCTAGAGCAATTTTGTTTTGAAGAGCACAGAACACCCTGTTCTGAATGTGGCTGGCACATGAACTCGATGTGCTGACAGCAATTTGTACTCCGATTAAAATAGGGGGGAAAAAAGGAAAGAGAGTGCACAGCAGTAA
           |||||||||||||||||||||||||||||||||||||||||||||||||||||||||||||||||||||||||||||||||||||||||||||||||||||||||||||||||||||||||||||||||||||||||||||||||||||||||||||||||||||||||||||||
reference  ACTTAAACTGGAGCTCTGACTTATTGTTCTCTTACTGCCCTAGAGCAATTTTGTTTTGAAGAGCACAGAACACCCTGTTCTGAATGTGGCTGGCACATGAACTCGATGTGCTGACAGCAATTTGTACTCCGATTAAAATAGGGGGGAAAAAAGGAAAGAGAGTGCACAGCAGTAA
                                                                            ^^^^^^^^^^^^^^^^
                                                                         3'-GTCTTGTGGGACAAGA-5' (sequence of interest)


   Allele: KE4-4-G02 R1+R2 [5/996] rank6 %totalreads:0.5 percentile:96 %top10reads:0.58 %readsfilteredfor1%:None %readsfilteredfor10%:None | mutant, likely deletion, 2 bp
    Locus: Homo sapiens chromosome 6, GRCh38.p12 Primary Assembly, ref|NC_000006.12| 35602190-35602016

                                                                                       5'-GTGCCAGCCACATTCAGAAC-3' (guide sequence)
                                                                                                          v
    query  TTACTGCTGTGCACTCTCTTTCCTTTTTTCCCCCCTATTTTAATCGGAGTACAAATTGCTGTCAGCACATCGAGTTCATGTGCCAGCCACATTCAG--CAGGGTGTTCTGTGCTCTTCAAAACAAAATTGCTCTAGGGCAGTAAGAGAACAATAAGTCAGAGCTCCAGTTTAAGT
           ||||||||||||||||||||||||||||||||||||||||||||||||||||||||||||||||||||||||||||||||||||||||||||||||  |||||||||||||||||||||||||||||||||||||||||||||||||||||||||||||||||||||||||||||
reference  TTACTGCTGTGCACTCTCTTTCCTTTTTTCCCCCCTATTTTAATCGGAGTACAAATTGCTGTCAGCACATCGAGTTCATGTGCCAGCCACATTCAGAACAGGGTGTTCTGTGCTCTTCAAAACAAAATTGCTCTAGGGCAGTAAGAGAACAATAAGTCAGAGCTCCAGTTTAAGT
                                                                                                         ^^^^^^^^^^^^^^^^
                                                                                                      5'-AGAACAGGGTGTTCTG-3' (sequence of interest)


   Allele: KE4-4-G02 R1+R2 [4/996] rank7 %totalreads:0.4 percentile:95 %top10reads:0.47 %readsfilteredfor1%:None %readsfilteredfor10%:None | mutant, likely deletion, 5 bp
    Locus: Homo sapiens chromosome 6, GRCh38.p12 Primary Assembly, ref|NC_000006.12| 35602016-35602190

                                                                                    3'-CAAGACTTACACCGACCGTG-5' (guide sequence)
                                                                                          v
    query  ACTTAAACTGGAGCTC-GACTTATTGTTCTCTTACTGCCCTAGAGCAATTTTGTTTTGAAGAGCACAGAACACCCTGTT----ATGTGGCTGGCACATGAACTCGATGTGCTGACAGCAATTTGTACTCCGATTAAAATAGGGGGGAAAAAAGGAAAGAGAGTGCACAGCAGTAA
           |||||||||||||||| ||||||||||||||||||||||||||||||||||||||||||||||||||||||||||||||    ||||||||||||||||||||||||||||||||||||||||||||||||||||||||||||||||||||||||||||||||||||||||||||
reference  ACTTAAACTGGAGCTCTGACTTATTGTTCTCTTACTGCCCTAGAGCAATTTTGTTTTGAAGAGCACAGAACACCCTGTTCTGAATGTGGCTGGCACATGAACTCGATGTGCTGACAGCAATTTGTACTCCGATTAAAATAGGGGGGAAAAAAGGAAAGAGAGTGCACAGCAGTAA
                                                                            ^^^^^^^^^^^^^^^^
                                                                         3'-GTCTTGTGGGACAAGA-5' (sequence of interest)
     .
     .
     .
     .
     .
     . etc.
```

(d) ***allele_definitions.pdf*** presents a visualization of ranked allele frequencies (raw frequency relative to all other reads, and adjusted frequencies relative to reads at various abundance thresholds). This output is intended to visually aid the plausibility of sample-specific genotype inferences, given that most diploid clonal samples 'step' down from one or two primary Allele Ranks with high read representation to relatively negligible Allele Ranks with very low read representation (as observed for sample 4-4 G02, shown). Alternatively, multiploid or mixed variant populations are distinguishable by Allele Ranks beyond two—without clear single (homozygous) or binary (heterozygous) representatives.

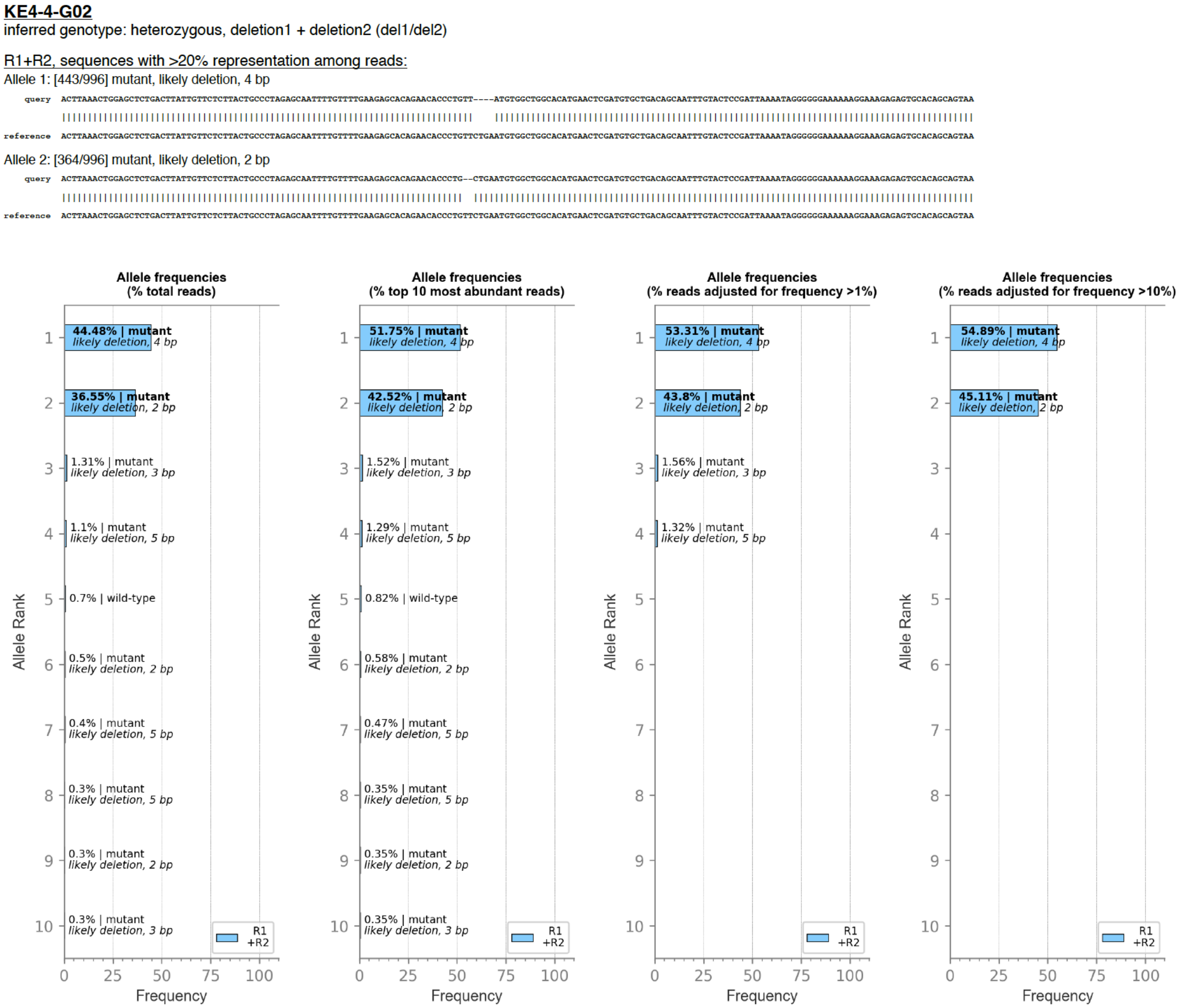

(e) ***population_summary.txt*** reports aggregate genotype and allele representations across all samples (“*I. Synopsis of Interpretations: Allele Definitions & Genotype Inferences”),* as well as “Ranked Alleles” deprecated from analysis due to 1) no hits or 2) multi-mapping hits in reference database (BLASTN), or 3) (*if characterized by >1 high-scoring pair [hsp])* overlapping hsp’s or hsp span exceeding 1 kb *(“II. Synopsis of Reads Lost to Analysis”*). Properties of these deprecated Ranked Alleles (‘reads’) can be manually examined by interested users by referring to their read ID as a defline in fasta.fa, or in blastn_alignments.txt.

```
Genotypes.py: Population Summary
Date: 01/03/2021

I. Synopsis of Interpretations: Allele Definitions & Genotype Inferences

    (A) Sample summary
        (i) Number of samples processed: 384
        (ii) % samples called (genotype inferred): 379 (98.7%)

    (B) Genotypes summary
        (i) % samples diploid (1-2 prominent alleles inferred): 367 (95.57%)
            (1) % homozygous wild-type (wt): 317 (82.55%)
            (2) % homozygous mutant: 17 (4.43%)
                -> % homozygous deletion: 13 (3.39%)
                -> % homozygous insertion: 0 (0.0%)
                -> % homozygous substitution: 4 (1.04%)
                -> % homozygous complex indel: 0 (0.0%)
            (3) % heterozygous (wt + mutant): 26 (6.77%)
                -> % heterozygous deletion: 13 (3.39%)
                -> % heterozygous insertion: 0 (0.0%)
                -> % heterozygous substitution: 13 (3.39%)
                -> % heterozygous complex indel: 0 (0.0%)
            (4) % heterozygous (mutant + mutant): 7 (1.82%)
                -> % heterozygous deletion + insertion: 0 (0.0%)
                -> % heterozygous deletion + substitution: 1 (0.26%)
                -> % heterozygous insertion + substitution: 0 (0.0%)
                -> % heterozygous deletion + complex indel: 0 (0.0%)
                -> % heterozygous insertion + complex indel: 1 (0.26%)
                -> % heterozygous substitution + complex indel: 0 (0.0%)
        (ii) % samples multiploid (>2 prominent alleles inferred): 5 (1.3%)

    (B) Alleles summary
        (i) % wild-type alleles: 653 (89.33% of total alleles)
        (ii) % mutant alleles: 78 (10.67% of total alleles)
            (1) % deletion alleles: 55 (7.52% of total alleles)
            (2) % insertion alleles: 2 (0.27% of total alleles)
            (3) % substitution alleles: 25 (3.42% of total alleles)
            (4) % complex indel alleles: 0 (0.0% of total alleles)

II. Synopsis of Reads Lost to Analysis
    Reads among the 'Top 10' reads are deprecated (not analyzed by Genotypes.py) if they fall into the following categories:
    (A) no hits, (B) multiple hits, or (C) (if >1 hsp for 1 hit) overlapping hsp's in reference database and/or hsp span (end-to-end) that
        exceeds 1 kb

    (A) Samples with reads among the 'top 10 most abundant reads', that did not map to the reference genome
        (i) For the following sample IDs (1), NO reads among the "top 10 most abundant reads" could be mapped to the reference genome:
             KE4-1-D06
        (ii) For the following sample IDs (45), the indicated reads among the "top 10 most abundant reads" did not map to the reference
             genome:
             KE4-1-B05:
               KE4-1-B05_R1+R2_[1/82]_rank7_%totalreads:1.22_percentile:47_%top10reads:1.43_%readsfilteredfor1%:1.22_%readsfilteredfor10%:None
             KE4-1-C07:
               KE4-1-C07_R1+R2_[1/403]_rank8_%totalreads:0.25_percentile:44_%top10reads:0.28_%readsfilteredfor1%:None_%readsfilteredfor10%:None
             KE4-1-C10:
               KE4-1-C10_R1+R2_[2/50]_rank4_%totalreads:4.0_percentile:88_%top10reads:5.26_%readsfilteredfor1%:4.0_%readsfilteredfor10%:None
               KE4-1-C10_R1+R2_[1/50]_rank10_%totalreads:2.0_percentile:43_%top10reads:2.63_%readsfilteredfor1%:2.0_%readsfilteredfor10%:None
               .
               . etc.
               .
    (B) Samples with reads among the "top 10 most abundant reads", that mapped to multiple loci in the reference genome
        (i) For the following sample IDs (28), the indicated reads among the "top 10 most abundant reads" mapped to more than one locus in
            the reference genome:
              KE4-1-B11
              KE4-1-D11
               .
               . etc.
               .
              Details:
              KE4-1-B11
              =========
              Read: KE4-1-B11_R1+R2_[3/368]_rank3_%totalreads:0.82_percentile:95_%top10reads:0.94_%readsfilteredfor1%:None_%readsfilteredfor10%:None
              Hit 1: Homo sapiens chromosome 16, GRCh38.p12 Primary Assembly: ref|NC_000016.10|, 33966746-33966594
AGC-TCTGA--CTTATTGTTCCTGAGATGAGAAAATCATCTCTAATCACACATCACAGAGCAAATCTGTAAACAAGAGTGTTTCTATTGAAGATCCGGGGGGATCTGAACACCAGGCAGGTGCTGGAGACACTGTTTCAGGAGTGCACAGCAG
||| |||||  ||||||||||||||||||||||||||||||||||||||||||||||||||||||||||||||||||||||||||||||||||||||||||||||||||||||||||||||||||||||||||||||| ||||||
AGCTTCTGATTCTTATTGTTCCTGAGATGAGAAAATCATCTCTAATCACACATCACAGAGCAAATCTGTAAACAAGAGTGTTTCTATTGAAGATCCGGGGGGATCTGAACACCAGGCAGGTGCTGGAGACACTGTTTCAGGAGTGCCCAGCAG
              Hit 2: Homo sapiens chromosome 16 unlocalized genomic scaffold, GRCh38.p12 Primary Assembly HSCHR16_RANDOM_CTG1: ref|NT_187383.1|, 1156890-1157042
AGC-TCTGA--CTTATTGTTCCTGAGATGAGAAAATCATCTCTAATCACACATCACAGAGCAAATCTGTAAACAAGAGTGTTTCTATTGAAGATCCGGGGGGATCTGAACACCAGGCAGGTGCTGGAGACACTGTTTCAGGAGTGCACAGCAG
||| |||||  ||||| ||||||||||||||||||||||||||||||||||||||||||||||||||||||||||||||| ||||||||||||||||||||||||||||||||||||||||||||||||| ||||||
AGCTTCTGATTCTTATGGTTCCTGAGATGAGAAAATCATCTCTAATCACACATCACAGAGCAAATCTGTAAACAAGAGTGTTTCTATTGAAGATCCTGGGGGATCTGAACACCAGGCAGGTGCTGGAGACACTGTTTCAGGAGTGCCCAGCAG
```

```
     Hit 3: Homo sapiens chromosome 14 genomic scaffold, GRCh38.p12 alternate locus group ALT_REF_LOCI_1 HSCHR14_3_CTG1: ref|NT_187600.1|, 643201-643049
AGC-TCTGA--CTTATTGTTCCTGAGATGAGAAAATCATCTCTAATCACACATCACAGAGCAAATCTGTAAACAAGAGTGTTTCTATTGAAGATCCGGGGGGATCTGAACACCAGGCAGGTGCTGGAGACACTGTTTCAGGAGTGCACAGCAG
||| |||||  |||||||||||||||||||||||||||||||||||||||||||||| |||||||||||||||||||||||||||| |||||||| ||||||||||||||||||||||||||||||||| |||||||||||| || ||||||
AGCTTCTGATTCTTATTGTTCCTGAGATGAGAAAATCATCTCTAATCACACATCACACAGCAAATCTGTAAACAAGAGTGTTTCTATGGAAGATCCTGGGGGATCTGAACACCAGGCAGGTGCTGGAGACCCTGTTTCAGGAGCGCCCAGCAG
     Hit 4: Homo sapiens chromosome 14, GRCh38.p12 Primary Assembly: ref|NC_000014.9|, 106175432-106175280
AGC-TCTGA--CTTATTGTTCCTGAGATGAGAAAATCATCTCTAATCACACATCACAGAGCAAATCTGTAAACAAGAGTGTTTCTATTGAAGATCCGGGGGGATCTGAACACCAGGCAGGTGCTGGAGACACTGTTTCAGGAGTGCACAGCAG
||| |||||  |||||||||||||||||||||||||||||||||||||||||||||| |||||||||||||||||||||||||||| |||||||| ||||||||||||||||||||||||||||||||| |||||||||||| || ||||||
AGCTTCTGATTCTTATTGTTCCTGAGATGAGAAAATCATCTCTAATCACACATCACACAGCAAATCTGTAAACAAGAGTGTTTCTATGGAAGATCCTGGGGGATCTGAACACCAGGCAGGTGCTGGAGACCCTGTTTCAGGAGCGCCCAGCAG
     Hit 5: Homo sapiens chromosome 6, GRCh38.p12 Primary Assembly: ref|NC_000006.12|, 35602016-35602044
ACTTAAACTGGAGCTCTGACTTATTGTTC
|||||||||||||||||||||||||||||
ACTTAAACTGGAGCTCTGACTTATTGTTC

  KE4-1-D11
  =========
  Read: KE4-1-D11_R1+R2_[1/278]_rank10_%totalreads:0.36_percentile:45_%top10reads:0.43_%readsfilteredfor1%:None_%readsfilteredfor10%:None
  Hit 1: Homo sapiens chromosome 1, GRCh38.p12 Primary Assembly: ref|NC_000001.11|, 183919052-183918909
  CTGGAGCTCTTTGGAATATTCAATGCTGTTTCAGAGTATAAAGGGGTCCTGAGAACAAAAAGTTTGAGAACTGCTAGTCTCGCCTGAACCTGGACTGATTTATTTCCATCTTCTGGTCAACAGCACACAGCATAATACCTTTCA
  |||||||||||||||||||||||||||||||||||||||||||||||||||||||||||||||||||||||||||||||||||||||||||||||||||||||||||||||||||||||||||||||||||||||||| |||||
  CTGGAGCTCTTTGGAATATTCAATGCTGTTTCAGAGTATAAAGGGGTCCTGAGAACAAAAAGTTTGAGAACTGCTAGTCTCGCCTGAACCTGGACTGATTTATTTCCATCTTCTGGTCAACAGCACACAGCATAATACATTTCA
  Hit 2: Homo sapiens chromosome 16, GRCh38.p12 Primary Assembly: ref|NC_000016.10|, 75359285-75359326
  TAAAGGGGTCCTGAGAACAAAAAGTTTGAGAACTGCT-AGTC
  ||||| |||||||||||||||||||||||||||| || ||||
  TAAAGAGGTCCTGAGAACAAAAAGTTTGAGAACTACTGAGTC
```

.

. etc.

.

C) Samples with reads among the 'top 10 most abundant reads', with high-scoring pairs (hsp's) that were attempted for reconstruction with BLASTDBCMD sequence retrieval across hsp span, but deprecated because the hsp's

a) overlapped in the reference genome (potential artefact or signature of inversion, duplication and/or

b) exceeded 1 kb span (end-to-end)

Consult fasta.fa and/or BLASTN output if any of these reads is of further interest to examine manually:

(i) For the following sample IDs (51), the indicated reads among the 'top 10 most abundant reads' exhibited overlapping hsp's aligned to the reference genome:

KE4-1-B05
KE4-1-B10

.

. etc.

.

Details:

KE4-1-B05

```
   =========
Read: KE4-1-B05_R1+R2_[1/82]_rank3_%totalreads:1.22_percentile:47_%top10reads:1.43_%readsfilteredfor1%:1.22_%readsfilteredfor10%:None
Hit: Homo sapiens chromosome 6, GRCh38.p12 Primary Assembly: ref|NC_000006.12|
Hsp 1: 35602001-35602046
    GAG-TGCACAGCAGTAACTTAAACTGGAGCTCTGACTTATTGTTCTC
    ||| |||| | || | |||||||||||||||||||||||||||||||
    GAGCTGCAAAACA-TCACTTAAACTGGAGCTCTGACTTATTGTTCTC
Hsp 2: 35602016-35602046
    ACTTAAACTGGAGCTCTGACTTATTGTTCTC
    |||||||||||||||||||||||||||||||
    ACTTAAACTGGAGCTCTGACTTATTGTTCTC

Read: KE4-1-B05_R1+R2_[1/82]_rank9_%totalreads:1.22_percentile:47_%top10reads:1.43_%readsfilteredfor1%:1.22_%readsfilteredfor10%:None
Hit: Homo sapiens chromosome 6, GRCh38.p12 Primary Assembly: ref|NC_000006.12|
Hsp 1: 35602016-35602166
    ACTTAAACTGGAGCTCTGACTTATTGTTCTCTTACTGCCCTAGAGCAATTTTGTTTTGAAGAGCACAGAACACCCTGTTCTGAATGTGGCTGGCACATGAACTCGATGTGCTGACAGCAATTTGTACTCCGATTAAAATAGGGGGGAAAAA
    |||||||||||||||||||||||||||||||||||||||||||||||||||||||||||||||||||||||||||||||||||||||||||||||||||||||||||||||||||||||||||||||||||||||||||||||||||||||
    ACTTAAACTGGAGCTCTGACTTATTGTTCTCTTACTGCCCTAGAGCAATTTTGTTTTGAAGAGCACAGAACACCCTGTTCTGAATGTGGCTGGCACATGAACTCGATGTGCTGACAGCAATTTGTACTCCGATTAAAATAGGGGGGAAAAA
Hsp 2: 35602041-35602190
    GTTCTCTTACGGTCCTATAGCAATTTTGTTTTGAAGAGCACAGAACACCATGTATTGAATGTGGCTGGCACATGAACGCGAAGTGCTGACAGCAATTGGTACTCCGATTAAAATAGGGGGGAAAAAAGGAAAGAGAGTGCACAGCAGTAA
    |||||||||| | |||| ||||||||||||||||||||||||||||||| |||  |||||||||||||||||||||| ||| ||||||||||||||| ||||||||||||||||||||||||||||||||||||||||||||||||||||
    GTTCTCTTACTGCCCTAGAGCAATTTTGTTTTGAAGAGCACAGAACACCCTGTTCTGAATGTGGCTGGCACATGAACTCGATGTGCTGACAGCAATTTGTACTCCGATTAAAATAGGGGGGAAAAAAGGAAAGAGAGTGCACAGCAGTAA

Read: KE4-1-B05_R1+R2_[1/82]_rank10_%totalreads:1.22_percentile:47_%top10reads:1.43_%readsfilteredfor1%:1.22_%readsfilteredfor10%:None
Hit: Homo sapiens chromosome 6, GRCh38.p12 Primary Assembly: ref|NC_000006.12|
Hsp 1: 35602016-35602046
    ACTTAAACTGGAGCTCTGACTTATTGTTCTC
    |||||||||||||||||||||||||||||||
    ACTTAAACTGGAGCTCTGACTTATTGTTCTC
Hsp 2: 35602017-35602046
    CTTAAACTGGAGCTCTGACTTATTGTTCTC
    ||||||||||||||||||||||||||||||
    CTTAAACTGGAGCTCTGACTTATTGTTCTC


KE4-1-B10
=========
Read: KE4-1-B10_R1+R2_[1/540]_rank8_%totalreads:0.19_percentile:44_%top10reads:0.2_%readsfilteredfor1%:None_%readsfilteredfor10%:None
Hit: Homo sapiens chromosome 6, GRCh38.p12 Primary Assembly: ref|NC_000006.12|
Hsp 1: 35602016-35602166
    ACTTAAACTGGAGCTCTGACTTATTGTTCTCTTACTGCCCTAGAGCAATTTTGTTTTGAAGAGCACAAAACACCCTGTTCTGAATGTGGCTGGCACATGAACTCGATGTGCTGACAGCAATTTGTACTCCGATTAAAATAGGGGGGAAAAA
    ||||||||||||||||||||||||||||||||||||||||||||||||||||||||||||||||||| |||||||||||||||||||||||||||||||||||||||||||||||||||||||||||||||||||||||||||||||||||
    ACTTAAACTGGAGCTCTGACTTATTGTTCTCTTACTGCCCTAGAGCAATTTTGTTTTGAAGAGCACAGAACACCCTGTTCTGAATGTGGCTGGCACATGAACTCGATGTGCTGACAGCAATTTGTACTCCGATTAAAATAGGGGGGAAAAA
Hsp 2: 35602055-35602190
    CTAGAGCATGTTAGTTTGCAAGAGCAGACAACACACAGTTCGGAATGTGGATGAAACATGAACACGAGGAGCTGACAGCAAGTTGTACACCGATTAAAATAGGGGGGAAAAAAGGAAAGAGAGTACACAGCAGTAA
    ||||||||  || ||||  ||||||| | ||||| | |||| |||||||| ||  |||||||| ||| | ||||||||||| |||||| ||||||||||||||||||||||||||||||||||| |||||||||||
    CTAGAGCAATTTTGTTTTGAAGAGCACAGAACACCCTGTTCTGAATGTGGCTGGCACATGAACTCGATGTGCTGACAGCAATTTGTACTCCGATTAAAATAGGGGGGAAAAAAGGAAAGAGAGTGCACAGCAGTAA
```

.

.

.

.

.

.

.

.

.

. etc.

(f) ***script_metrics.txt*** logs script operation metadata, including operating system information, user-entered variables, fastq file information, fastq files processed, file output information (output files, sizes), and script operation times (total computing time).

```
Genotypes.py: Script Metrics
Date: 01/03/2021

Operating system information:
    name: Kirks-MBP.attlocal.net
    platform: Darwin-17.7.0-x86_64-i386-64bit
    RAM (GB): 16.0
    physical CPU/effective CPU: 4/8
    executable: /Library/Frameworks/Python.framework/Versions/3.7/Resources/Python.app/Contents/MacOS/Python

User-entered variables:
    output_directory: /Users/kirkehmsen/Documents/GenotypesOutput
    fastq_directory: /Users/kirkehmsen/Documents/Zenodo/ExampleTestFiles/Genotypes_testfiles/fastq_files
    blastn_path: /Users/kirkehmsen/anaconda3/bin/blastn
    db_path: /Users/kirkehmsen/Documents/Zenodo/ExampleTestFiles/Genotypes_testfiles/blastn_database
    db_prefix: GRCh38
    blastdbcmd_path: /Users/kirkehmsen/anaconda3/bin/blastdbcmd
    guideRNA_seq: GTGCCAGCCACATTCAGAAC
    extant_seq: AGAACAGGGTGTTCTG

fastq file information:
    Illumina sequencing run ID(s): @M00582:216
    Number of fastq files processed: 768
    Size distribution of fastq files processed:
        total... 47 MB
        range... max: 0.16 MB; min: 0.0009 MB; median: 0.061 MB; mean +/- stdev: 0.062 +/- 0.031 MB
    Read distribution within fastq files to process:
        total... 569,374 reads
        range... max: 1745 reads; min: 2 reads; median: 754.0 reads; mean +/- stdev: 741.0 +/- 382.0 reads

fastq files processed (name, size (MB), reads):
    /Users/kirkehmsen/Documents/Zenodo/ExampleTestFiles/Genotypes_testfiles/fastq_files/KE4-1-A01_S1153_L001_R1_001.fastq, 0.13484, 389
    /Users/kirkehmsen/Documents/Zenodo/ExampleTestFiles/Genotypes_testfiles/fastq_files/KE4-1-A01_S1153_L001_R2_001.fastq.gz, 0.03843, 389
    /Users/kirkehmsen/Documents/Zenodo/ExampleTestFiles/Genotypes_testfiles/fastq_files/KE4-1-A02_S1154_L001_R1_001.fastq.gz, 0.05263, 751
    /Users/kirkehmsen/Documents/Zenodo/ExampleTestFiles/Genotypes_testfiles/fastq_files/KE4-1-A02_S1154_L001_R2_001.fastq.gz, 0.06725, 751
    /Users/kirkehmsen/Documents/Zenodo/ExampleTestFiles/Genotypes_testfiles/fastq_files/KE4-1-A03_S1155_L001_R1_001.fastq.gz, 0.04951, 677
    /Users/kirkehmsen/Documents/Zenodo/ExampleTestFiles/Genotypes_testfiles/fastq_files/KE4-1-A03_S1155_L001_R2_001.fastq.gz, 0.06415, 677
    .
    .
    .
    .
    .
    .
    .
    .
    .
    . etc.


    File output information:
        Output directory: /Users/kirkehmsen/Documents/GenotypesOutput
        Total file #: 7
        Total file output sizes:
            01032021_allele_definitions.txt: 3.7 MB
            01032021_genotypes.txt: 3.7 MB
            01032021_script_metrics.txt: 105.5 KB
            01032021_allele_evidence.pdf: 80.2 MB
            01032021_population_summary.txt: 565.1 KB
            01032021_blastn_alignments.txt: 43.2 MB
            01032021_fasta.fa: 1.1 MB

    Script operation times:
        start time: 22:42:21
        fasta processing time: 0 hr|00 min|23 sec|715762 microsec
        alignments processing time: 0 hr|00 min|38 sec|341546 microsec
        genotype inference processing time: 0 hr|00 min|12 sec|783026 microsec
        frequency plots compilation time: 2 hr|54 min|40 sec|869292 microsec
        accessory file processing time: 0 hr|00 min|08 sec|601552 microsec
        total processing time: 2 hr|55 min|53 sec|460931 microsec
        end time: 01:38:15
```

Supplementary Figure S10. File outputs of CollatedMotifs.py.

Example output files are available in the **ExampleOutputFiles** directory of the associated Zenodo repository (**DOI 10.5281/zenodo.3406861**).

(a) Example of custom-named directory (CollatedMotifs.py user *input #1*) populated with 6 output files and 3 sub-directories; filenames are automatically prefixed by script operations with system start date. ***fasta.fa, blastn_alignments.txt, markov_background.txt,*** and the three sub-directories are prepared during script operations and support script operations, but key script output content is contained in ***collated_TFBS.txt*** and ***collated_TFBS.xlsx***, with script operation parameters logged in ***script_metrics.txt***.

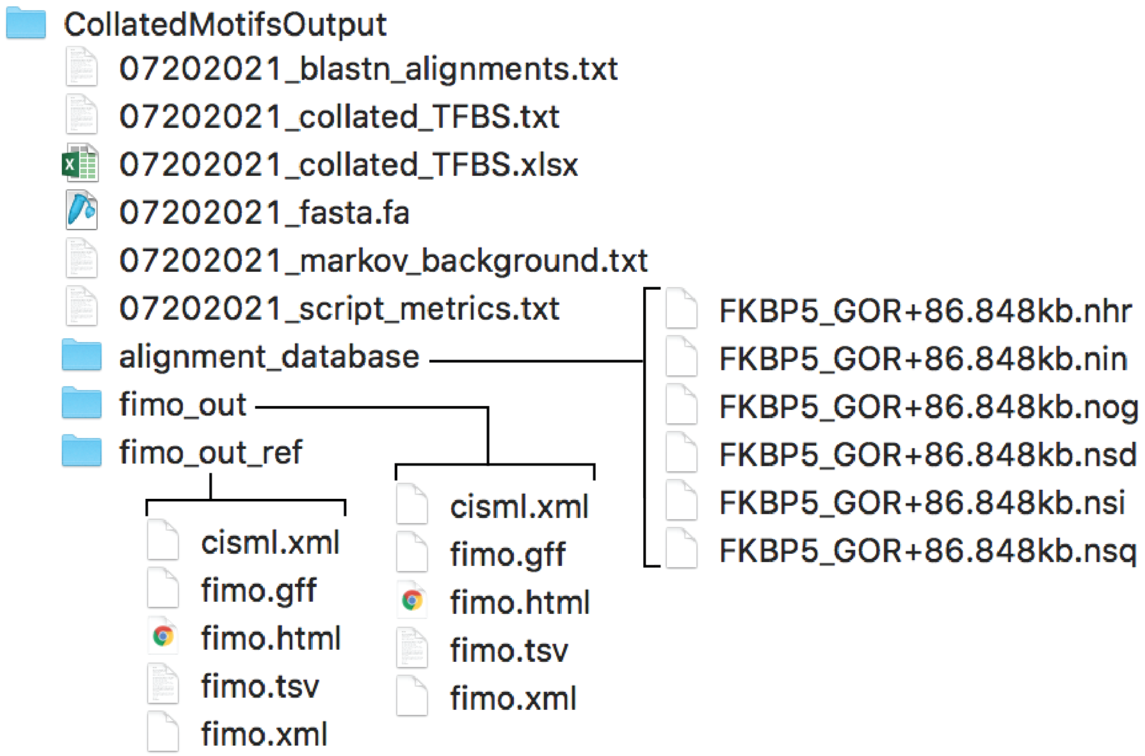

(b) ***collated_TFBS.txt*** reports the top 5 ranked alleles for R1+R2 (for paired-end/PE data), with allele frequency metrics in the '*Allele*' definition. '*Motifs*' indicates the total number of distinct TFBS identified in the allele, along with the number of distinct TFs that comprise these TFBS; '*Synopsis*' summarizes the # of lost sites and # of new TFBS relative to a reference sequence; '*Details'* summarizes the TF identities associated with the TFBS differences. *NEW motifs* are mapped above the allele alignment ('+ strand' indicating the sequence as reported, '-strand' indicating its implicit complement); *LOST motifs* are mapped below the allele alignment.

```
========
KE4-4-G02
========
  Allele: KE4-4-G02 | R1+R2 | [443/996] | %totalreads:44.48 | percentile:100 | %top5reads:52.86 | %readsfilteredfor1%:53.31 | %readsfilteredfor10%:54.89
  Motifs: total distinct sites |30|, total unique TFs |23| (motifs for 7 TFs occur >1x)
Synopsis: relative to reference sequence--# lost sites |6|, # new sites |5|
 Details: lost |Ar:2, NR3C1:2, NR3C2:2|
           new |Ar:2, NR3C1:2, NR3C2:1|

          NEW motifs:
          plus(+) strand:
                                                                           CAGAACACCCTGTTATG |-- NR3C1 (MA0113.3) (pval 7.71e-05) [note, approx. position]
                                                                           CAGAACACCCTGTTATG |-- Ar (MA0007.3) (pval 7.86e-05) [note, approx. position]
          minus(-) strand:
                                                                               GTCTTGTGGGACAATAC |-- NR3C2 (MA0727.1) (pval 4.99e-05) [note, approx. position]
                                                                               GTCTTGTGGGACAATAC |-- NR3C1 (MA0113.3) (pval 5.74e-05) [note, approx. position]
                                                                               GTCTTGTGGGACAATAC |-- Ar (MA0007.3) (pval 6.74e-05) [note, approx. position]

   query  ACTTAAACTGGAGCTCTGACTTATTGTTCTCTTACTGCCCTAGAGCAATTTTGTTTTGAAGAGCACAGAACACCCTGTT----ATGTGGCTGGCACATGAACTCGATGTGCTGACAGCAATTTGTACTCCGATTAAAATAGGGGGGAAAAAAGGAAAGAGAGTGCACAGCAGTAA
          |||||||||||||||||||||||||||||||||||||||||||||||||||||||||||||||||||||||||||||||    ||||||||||||||||||||||||||||||||||||||||||||||||||||||||||||||||||||||||||||||||||||||||||||
reference ACTTAAACTGGAGCTCTGACTTATTGTTCTCTTACTGCCCTAGAGCAATTTTGTTTTGAAGAGCACAGAACACCCTGTTCTGAATGTGGCTGGCACATGAACTCGATGTGCTGACAGCAATTTGTACTCCGATTAAAATAGGGGGGAAAAAAGGAAAGAGAGTGCACAGCAGTAA

          LOST motifs:
          plus(+) strand:
                                                                           CAGAACACCCTGTTCTG |-- NR3C2 (MA0727.1) (pval 2.62e-06)
                                                                           CAGAACACCCTGTTCTG |-- Ar (MA0007.3) (pval 3.26e-06)
                                                                           CAGAACACCCTGTTCTG |-- NR3C1 (MA0113.3) (pval 2.95e-06)
          minus(-) strand:
                                                                           GTCTTGTGGGACAAGAC |-- Ar (MA0007.3) (pval 1.66e-06)
                                                                           GTCTTGTGGGACAAGAC |-- NR3C1 (MA0113.3) (pval 2.03e-06)
                                                                           GTCTTGTGGGACAAGAC |-- NR3C2 (MA0727.1) (pval 1.67e-06)

  Allele: KE4-4-G02 | R1+R2 | [364/996] | %totalreads:36.55 | percentile:99 | %top5reads:43.44 | %readsfilteredfor1%:43.8 | %readsfilteredfor10%:45.11
  Motifs: total distinct sites |25|, total unique TFs |21| (motifs for 4 TFs occur >1x)
Synopsis: relative to reference sequence--# lost sites |6|, # new sites |0|
 Details: lost |Ar:2, NR3C1:2, NR3C2:2|
           new ||

   query  ACTTAAACTGGAGCTCTGACTTATTGTTCTCTTACTGCCCTAGAGCAATTTTGTTTTGAAGAGCACAGAACACCCTG--CTGAATGTGGCTGGCACATGAACTCGATGTGCTGACAGCAATTTGTACTCCGATTAAAATAGGGGGGAAAAAAGGAAAGAGAGTGCACAGCAGTAA
          |||||||||||||||||||||||||||||||||||||||||||||||||||||||||||||||||||||||||||||  ||||||||||||||||||||||||||||||||||||||||||||||||||||||||||||||||||||||||||||||||||||||||||||||||
reference ACTTAAACTGGAGCTCTGACTTATTGTTCTCTTACTGCCCTAGAGCAATTTTGTTTTGAAGAGCACAGAACACCCTGTTCTGAATGTGGCTGGCACATGAACTCGATGTGCTGACAGCAATTTGTACTCCGATTAAAATAGGGGGGAAAAAAGGAAAGAGAGTGCACAGCAGTAA

          LOST motifs:
          plus(+) strand:
                                                                           CAGAACACCCTGTTCTG |-- NR3C2 (MA0727.1) (pval 2.62e-06)
                                                                           CAGAACACCCTGTTCTG |-- Ar (MA0007.3) (pval 3.26e-06)
                                                                           CAGAACACCCTGTTCTG |-- NR3C1 (MA0113.3) (pval 2.95e-06)
          minus(-) strand:
                                                                           GTCTTGTGGGACAAGAC |-- Ar (MA0007.3) (pval 1.66e-06)
                                                                           GTCTTGTGGGACAAGAC |-- NR3C1 (MA0113.3) (pval 2.03e-06)
                                                                           GTCTTGTGGGACAAGAC |-- NR3C2 (MA0727.1) (pval 1.67e-06)

  Allele: KE4-4-G02 | R1+R2 | [13/996] | %totalreads:1.31 | percentile:98 | %top5reads:1.55 | %readsfilteredfor1%:1.56 | %readsfilteredfor10%:None
  Motifs: total distinct sites |27|, total unique TFs |23| (motifs for 4 TFs occur >1x)
Synopsis: relative to reference sequence--# lost sites |6|, # new sites |2|
 Details: lost |Ar:2, NR3C1:2, NR3C2:2|
           new |SOX9:1, Sox6:1|

          NEW motifs:
          plus(+) strand:
                             CTATTGTTC |-- SOX9 (MA0077.1) (pval 1.53e-05) [note, approx. position]
                             CTATTGTTCT |-- Sox6 (MA0515.1) (pval 8e-05) [note, approx. position]

   query  ACTTAAACTGGAGCTCTGAC-TATTGTTCTCTTACTGCCCTAGAGCAATTTTGTTTTGAAGAGCACAGAACACCCTG--CTGAATGTGGCTGGCACATGAACTCGATGTGCTGACAGCAATTTGTACTCCGATTAAAATAGGGGGGAAAAAAGGAAAGAGAGTGCACAGCAGTAA
          |||||||||||||||||||| ||||||||||||||||||||||||||||||||||||||||||||||||||||||||  ||||||||||||||||||||||||||||||||||||||||||||||||||||||||||||||||||||||||||||||||||||||||||||||||
reference ACTTAAACTGGAGCTCTGACTTATTGTTCTCTTACTGCCCTAGAGCAATTTTGTTTTGAAGAGCACAGAACACCCTGTTCTGAATGTGGCTGGCACATGAACTCGATGTGCTGACAGCAATTTGTACTCCGATTAAAATAGGGGGGAAAAAAGGAAAGAGAGTGCACAGCAGTAA

          LOST motifs:
          plus(+) strand:
                                                                           CAGAACACCCTGTTCTG |-- NR3C2 (MA0727.1) (pval 2.62e-06)
                                                                           CAGAACACCCTGTTCTG |-- Ar (MA0007.3) (pval 3.26e-06)
                                                                           CAGAACACCCTGTTCTG |-- NR3C1 (MA0113.3) (pval 2.95e-06)
          minus(-) strand:
                                                                           GTCTTGTGGGACAAGAC |-- Ar (MA0007.3) (pval 1.66e-06)
                                                                           GTCTTGTGGGACAAGAC |-- NR3C1 (MA0113.3) (pval 2.03e-06)
                                                                           GTCTTGTGGGACAAGAC |-- NR3C2 (MA0727.1) (pval 1.67e-06)

  Allele: KE4-4-G02 | R1+R2 | [11/996] | %totalreads:1.1 | percentile:97 | %top5reads:1.31 | %readsfilteredfor1%:1.32 | %readsfilteredfor10%:None
  Motifs: total distinct sites |32|, total unique TFs |25| (motifs for 7 TFs occur >1x)
Synopsis: relative to reference sequence--# lost sites |6|, # new sites |7|
 Details: lost |Ar:2, NR3C1:2, NR3C2:2|
           new |Ar:2, NR3C1:2, NR3C2:1, SOX9:1, Sox6:1|

          NEW motifs:
          plus(+) strand:
                                                                          CAGAACACCCTGTTATG |-- Ar (MA0007.3) (pval 7.86e-05) [note, approx. position]
                                                                            CAGAACACCCTGTTATG |-- NR3C1 (MA0113.3) (pval 7.71e-05) [note, approx. position]
                             CTATTGTTC |-- SOX9 (MA0077.1) (pval 1.53e-05) [note, approx. position]
                             CTATTGTTCT |-- Sox6 (MA0515.1) (pval 8e-05) [note, approx. position]
          minus(-) strand:
                                                                               GTCTTGTGGGACAATAC |-- NR3C1 (MA0113.3) (pval 5.74e-05) [note, approx. position]
                                                                               GTCTTGTGGGACAATAC |-- Ar (MA0007.3) (pval 6.74e-05) [note, approx. position]
                                                                               GTCTTGTGGGACAATAC |-- NR3C2 (MA0727.1) (pval 4.99e-05) [note, approx. position]

   query  ACTTAAACTGGAGCTCTGAC-TATTGTTCTCTTACTGCCCTAGAGCAATTTTGTTTTGAAGAGCACAGAACACCCTGTT----ATGTGGCTGGCACATGAACTCGATGTGCTGACAGCAATTTGTACTCCGATTAAAATAGGGGGGAAAAAAGGAAAGAGAGTGCACAGCAGTAA
          |||||||||||||||||||| ||||||||||||||||||||||||||||||||||||||||||||||||||||||||||    ||||||||||||||||||||||||||||||||||||||||||||||||||||||||||||||||||||||||||||||||||||||||||||
reference ACTTAAACTGGAGCTCTGACTTATTGTTCTCTTACTGCCCTAGAGCAATTTTGTTTTGAAGAGCACAGAACACCCTGTTCTGAATGTGGCTGGCACATGAACTCGATGTGCTGACAGCAATTTGTACTCCGATTAAAATAGGGGGGAAAAAAGGAAAGAGAGTGCACAGCAGTAA

          LOST motifs:
          plus(+) strand:
                                                                           CAGAACACCCTGTTCTG |-- NR3C2 (MA0727.1) (pval 2.62e-06)
                                                                           CAGAACACCCTGTTCTG |-- Ar (MA0007.3) (pval 3.26e-06)
                                                                           CAGAACACCCTGTTCTG |-- NR3C1 (MA0113.3) (pval 2.95e-06)
          minus(-) strand:
                                                                           GTCTTGTGGGACAAGAC |-- Ar (MA0007.3) (pval 1.66e-06)
                                                                           GTCTTGTGGGACAAGAC |-- NR3C1 (MA0113.3) (pval 2.03e-06)
                                                                           GTCTTGTGGGACAAGAC |-- NR3C2 (MA0727.1) (pval 1.67e-06)

  Allele: KE4-4-G02 | R1+R2 | [7/996] | %totalreads:0.7 | percentile:97 | %top5reads:0.84 | %readsfilteredfor1%:None | %readsfilteredfor10%:None
  Motifs: total distinct sites |31|, total unique TFs |23| (motifs for 7 TFs occur >1x)
Synopsis: relative to reference sequence--# lost sites |0|, # new sites |0|
 Details: lost ||
           new ||

   query  ACTTAAACTGGAGCTCTGACTTATTGTTCTCTTACTGCCCTAGAGCAATTTTGTTTTGAAGAGCACAGAACACCCTGTTCTGAATGTGGCTGGCACATGAACTCGATGTGCTGACAGCAATTTGTACTCCGATTAAAATAGGGGGGAAAAAAGGAAAGAGAGTGCACAGCAGTAA
          |||||||||||||||||||||||||||||||||||||||||||||||||||||||||||||||||||||||||||||||||||||||||||||||||||||||||||||||||||||||||||||||||||||||||||||||||||||||||||||||||||||||||||||||
reference ACTTAAACTGGAGCTCTGACTTATTGTTCTCTTACTGCCCTAGAGCAATTTTGTTTTGAAGAGCACAGAACACCCTGTTCTGAATGTGGCTGGCACATGAACTCGATGTGCTGACAGCAATTTGTACTCCGATTAAAATAGGGGGGAAAAAAGGAAAGAGAGTGCACAGCAGTAA
```

(c) ***collated_TFBS.xlsx*** provides comma-separated panel data compatible with spreadsheet programs such as Excel, and/or import as panel data in Python pandas for user access and manipulation. This file's multiple worksheets (eight) contain raw content of *dict_allele_TFBS_synopsis,* a primary Python data object of CollatedMotifs.py (sample-specific Ranked Alleles and their TFBS content as identified by FIMO, relative to TFBS content of a reference sequence), with interpretations for TFBS alterations across alleles. Example Excel file is available at: **https://zenodo.org/record/3406861 (DOI 10.5281/zenodo.3406861**

Example worksheet names (TF of interest 'NR3C1')

1 TFBS, predicted lost, gained	3 NR3C1, lost (all)	5 NR3C1, lost (+regain)	7 NR3C1, curated samples
2 TFBS, lost-regained pairs	4 NR3C1, lost (-gain, -regain)	6 NR3C1, lost (+gain)	8 All TFBS in alleles

Worksheets 1 & 2: lost & gained TFBS summaries

- Worksheet 1 (*1 TFBS, predicted lost, gained*)
  Synopsis: data table of lost and gained TFBSs—encompassing all alleles, all samples
  Rows in worksheet 1 contain data for individual TFBSs identified as **lost or gained** in each sample-associated ranked allele (relative to reference sequence), detailing the following data in columns:
    - sample
    - allele rank (1 – up to 5)
    - allele ID (fasta defline for ranked allele, including sample name derived from original fastq file name input as sample reads source to CollatedMotifs.py and read count parameters such as reads/total reads, allele rank, % total reads, percentile, % top 5 reads, % reads filtered to exclude reads occurring at <1% frequency, % reads filtered to exclude reads occurring at <10% frequency)
    - reads
    - total reads
    - % total reads
    - % reads filtered for reads <1%
    - % reads filtered for reads <10%
    - alignment query (allele sequence)
    - alignment midline
    - alignment hit (reference)
    - TF
    - strand (+/-)
    - Lost TFBS sequence (in reference)
    - Lost TFBS start coordinate (in reference)
    - Lost TFBS end coordinate (in reference)
    - Gained TFBS sequence (not in reference at this position, novel to allele. *Note: this TFBS sequence is in the allele, 5'-3' on strand indicated in 'strand'
    - Gained TFBS start coordinate (in allele)
    - Gained TFBS end coordinate (in allele)
    - p-val
    - lost or gained in allele (relative to ref)?
    - comment

- this field notes whether the ranked allele detailed in the row is below 50 bp, potentially consistent with primer dimer in the sequenced reads *("note: inferred allele length <=50 bp; check read in fasta file if warranted, or pre-process fastq reads")*

- Worksheet 2 (*2 TFBS, lost-regained pairs*)
  Synopsis: data table of lost TFBSs, interpreted by CollatedMotifs.py as coinciding (or not) with 'regain' of a distinct TFBS for the same TF—encompassing all alleles, all samples
  Rows in worksheet 2 contain data for individual TFBSs identified as **lost** in each sample-associated ranked allele (relative to reference sequence), if allele has lost TFBS(s); if no TFBS has been identified as lost or gained relative to the reference in a ranked allele, the allele frequency and alignment data for the allele are logged in a single row included in the table, but the 'interpretation' field is labelled as '*no TFBS predicted as lost or gained in allele*'. The 'interpretation' field for each row represents the primary utility of worksheet 2, which is to provide an interpretation for whether a lost TFBS may in fact positionally coincide with a distinct TFBS for the same TF, termed here as a 'regain'. P-values in side-by-side columns for the "Lost TFBS" and "Regained TFBS" enable p-value comparison between the lost and regained TFBSs. The following data are detailed in columns:
    - sample
    - allele rank (1 – up to 5)
    - allele ID (fasta defline for ranked allele, including sample name derived from original fastq file name input as sample reads source to CollatedMotifs.py and read count parameters such as reads/total reads, allele rank, % total reads, percentile, % top 5 reads, % reads filtered to exclude reads occurring at <1% frequency, % reads filtered to exclude reads occurring at <10% frequency)
    - reads
    - total reads
    - % total reads
    - % reads filtered for reads <1%
    - % reads filtered for reads <10%
    - alignment query (allele sequence)
    - alignment midline
    - alignment hit (reference)
    - TF
    - strand (+/-)
    - Lost TFBS sequence (in reference)
    - Lost TFBS start coordinate (in reference)
    - Lost TFBS end coordinate (in reference)
    - Regained TFBS sequence (not in reference at this position, novel to allele. *Note: this TFBS sequence is in the allele, 5'-3' on strand indicated in 'strand'
    - Regained TFBS start coordinate (in allele)
    - Regained TFBS end coordinate (in allele)

- Lost TFBS p-val (in reference)
- Regained TFBS p-val (in allele)
- Interpretation
  - predicted lost-gained TFBS pair
  - predicted TFBS loss (TFBS lost in allele)
  - no TFBS predicted as lost or gained in allele
- comment
  - this field notes whether the ranked allele detailed in the row is below 50 bp, potentially consistent with primer dimer in the sequenced reads *("note: inferred allele length <=50 bp; check read in fasta file if warranted, or pre-process fastq reads")*

Worksheets 3-7: focus on TFBS losses for TF of interest

Worksheets 3-7 relate to interpretations performed only if a user has specified a "**TF of interest**" (*see input #11, B.3.c.xi Identity of a transcription factor (TF) of interest, to focus specialized analysis on samples and ranked alleles in which TFBS for this TF are predicted to have been lost)*. Where the term "TF of interest" occurs in the worksheet names below, this text is replaced in the output file by the user-specified TF identity (*e.g.*, *NR3C1*). If no "TF of interest" has been specified by user, these worksheets do not appear in *collated_TFBS.xlsx*.

- Worksheet 3 (*3 "TF of interest", lost (all)*)
  Synopsis: data table of alleles that have lost TFBSs for user-specified TF of interest—encompassing all alleles that have lost TFBS for TF of interest, all samples
  Rows in worksheet 3 contain data for individual TFBSs **for a user-specified TF of interest**, identified as **lost** in each sample-associated ranked allele (relative to reference sequence). The primary utility of worksheet 3 is to provide a spreadsheet collection of sample-specific alleles that have lost a TFBS for the TF of interest, enabling sorting based on sample and allele rank columns to quickly deduce whether a sample may have TFBS losses in some or all primary alleles (defined by high rank, *e.g.,* 1-2, based on high relative frequency). Note that this spreadsheet does not include interpretation yet for whether any of the lost TFBS(s) for the TF of interest may also positionally coincide with either restoration ('**regain**') of a FIMO-identified TFBS for the TF of interest (these data are logged in worksheet 5), and/or novel acquisition ('**gain**') of a FIMO-identified TFBS for a distinct TF (other than the TF of interest; these data are logged in worksheet 6). The following data are detailed in columns:
  - sample
  - allele rank (1 – up to 5)
  - allele ID (fasta defline for ranked allele, including sample name derived from original fastq file name input as sample reads source to CollatedMotifs.py and read count parameters such as reads/total reads, allele rank, % total reads, percentile, % top 5 reads, % reads filtered to exclude reads occurring at <1% frequency, % reads filtered to exclude reads occurring at <10% frequency)
  - reads

- total reads
- % total reads
- % reads filtered for reads <1%
- % reads filtered for reads <10%
- alignment query (allele sequence)
- alignment midline
- alignment hit (reference)
- TF lost
- TF lost strand (+/-)
- Lost TFBS sequence (in reference at this position, lost in allele) *Note: this TFBS sequence is in the reference, 5'-3' on strand indicated in 'strand'
- Lost TFBS start coordinate (in reference)
- Lost TFBS end coordinate (in reference)
- Lost TFBS p-val (in reference)
- comment
    - this field notes whether the ranked allele detailed in the row is below 35 bp, potentially consistent with primer dimer in the sequenced reads *("note: inferred allele length <=50 bp; check read in fasta file if warranted, or pre-process fastq reads")*

- Worksheet 4 (*4 "TF of interest", lost (-gain, -regain)*)
  Synopsis: data table of alleles that have lost TFBSs for user-specified TF of interest, without coincident (positionally overlapping) regain of distinct TFBS for TF of interest, and also without coincident gain of TFBS for novel TF—filtered from all alleles that have lost TFBS for TF of interest, all samples; particularly useful for identifying samples with alleles having exclusively lost a TFBS for the TF of interest (no regain/gain)
  Rows in worksheet 4 contain data for individual TFBSs **for a user-specified TF of interest**, identified as **lost** in each sample-associated ranked allele (relative to reference sequence) and further filtered (from worksheet 3) as lacking identification of a coinciding 'gain' or 'regain' of TFBS (*see definitions of 'gain' and 'regain' above for worksheet 3*). The primary utility of worksheet 4 is to provide a spreadsheet collection of sample-specific alleles that have lost a TFBS for the TF of interest *without coinciding substitution of this TFBS with any other identified TFBS*, enabling sorting based on sample and allele rank columns to quickly deduce whether a sample may have TFBS losses in some or all primary alleles (defined by high rank, *e.g.,* 1-2, based on high relative frequency). The following data are detailed in columns:
    - sample
    - allele rank (1 – up to 5)
    - allele ID (fasta defline for ranked allele, including sample name derived from original fastq file name input as sample reads source to CollatedMotifs.py and read count parameters such as reads/total reads, allele rank, % total reads, percentile, % top 5 reads, % reads filtered to exclude reads occurring at <1% frequency, % reads filtered to exclude reads occurring at <10% frequency)
    - reads

- total reads
- % total reads
- % reads filtered for reads <1%
- % reads filtered for reads <10%
- alignment query (allele sequence)
- alignment midline
- alignment hit (reference)
- TF lost
- TF lost strand (+/-)
- Lost TFBS sequence (in reference at this position, lost in allele) *Note: this TFBS sequence is in the reference, 5'-3' on strand indicated in 'strand'
- Lost TFBS start coordinate (in reference)
- Lost TFBS end coordinate (in reference)
- Lost TFBS p-val (in reference)
- comment
    - this field notes whether the ranked allele detailed in the row is below 35 bp, potentially consistent with primer dimer in the sequenced reads *("note: inferred allele length <=50 bp; check read in fasta file if warranted, or pre-process fastq reads")*

- <u>Worksheet 5 (*5 "TF of interest", lost (+regain)*)</u>
  Synopsis: data table of alleles that have lost TFBSs for user-specified TF of interest, <u>with</u> coincident (positionally overlapping) regain of distinct TFBS for TF of interest, and without coincident gain of TFBS for novel TF (i.e., substitution of TFBS for TF of interest with distinct TFBS for TF of interest)—filtered from all alleles that have lost TFBS for TF of interest, all samples
  Rows in worksheet 5 contain data for individual TFBSs **for a user-specified TF of interest**, identified as **lost** in each sample-associated ranked allele (relative to reference sequence) and further filtered (from worksheet 3) as having a coinciding '**regain**' of TFBS (*see definitions of 'gain' and 'regain' above for worksheet 3*). The primary utility of worksheet 5 is to provide a spreadsheet collection of sample-specific alleles that have lost a TFBS for the TF of interest *with coinciding substitution of this TFBS with another distinct TFBS for the TF of interest*, enabling sorting based on sample and allele rank columns to quickly deduce whether a sample may have TFBS losses in some or all primary alleles (defined by high rank, *e.g.,* 1-2, based on high relative frequency), and how the p-value of the 'regained' TFBS compares to the lost TFBS at the same relative position. The following data are detailed in columns:
    - sample
    - allele rank (1 – up to 5)
    - allele ID (fasta defline for ranked allele, including sample name derived from original fastq file name input as sample reads source to CollatedMotifs.py and read count parameters such as reads/total reads, allele rank, % total reads, percentile, % top 5 reads, % reads filtered to exclude reads occurring at <1% frequency, % reads filtered to exclude reads occurring at <10% frequency)

- reads
- total reads
- % total reads
- % reads filtered for reads <1%
- % reads filtered for reads <10%
- alignment query (allele sequence)
- alignment midline
- alignment hit (reference)
- TF lost
- TF gained
- TF lost strand (+/-)
- TF gained strand (+/-)
- Lost TFBS sequence (in reference at this position, lost in allele) *Note: this TFBS sequence is in the reference, 5'-3' on strand indicated in 'strand'
- Gained TFBS sequence (not in reference at this position, novel to allele) *Note: this TFBS sequence is in the allele, 5'-3' on strand indicated in 'strand'
- Lost TFBS start coordinate (in reference)
- Lost TFBS end coordinate (in reference)
- Gained TFBS coordinate start (in allele)
- Gained TFBS coordinate end (in allele)
- Lost TFBS p-val (in reference)
- Gained TFBS p-val (in allele)
- comment
    - this field notes whether the ranked allele detailed in the row is below 35 bp, potentially consistent with primer dimer in the sequenced reads *("note: inferred allele length <=50 bp; check read in fasta file if warranted, or pre-process fastq reads")*

- Worksheet 6 (*6 "TF of interest", lost (+gain)*)
  Synopsis: table of metadata for alleles that have lost TFBSs for user-specified TF of interest, without coincident (positionally overlapping) regain of distinct TFBS for TF of interest, but with gain of a TFBS for an entirely different TF—filtered from all alleles that have lost TFBS for TF of interest, all samples
  Rows in worksheet 6 contain data for individual TFBSs **for a user-specified TF of interest**, identified as **lost** in each sample-associated ranked allele (relative to reference sequence) and further filtered (from worksheet 3) as having a coinciding '**gain**' of TFBS (*see definitions of 'gain' and 'regain' above for worksheet 3*). The primary utility of worksheet 6 is to provide a spreadsheet collection of sample-specific alleles that have lost a TFBS for the TF of interest *with coinciding substitution of this TFBS with a TFBS for an entirely distinct TF*, enabling sorting based on sample and allele rank columns to quickly deduce whether a sample may have TFBS losses in some or all primary alleles (defined by high rank, *e.g.*, 1-2, based on high relative frequency), and the identity (and TFBS p-value) of the replacing TF. The following data are detailed in columns:
    - sample

- allele rank (1 – up to 5)
- allele ID (fasta defline for ranked allele, including sample name derived from original fastq file name input as sample reads source to CollatedMotifs.py and read count parameters such as reads/total reads, allele rank, % total reads, percentile, % top 5 reads, % reads filtered to exclude reads occurring at <1% frequency, % reads filtered to exclude reads occurring at <10% frequency)
- reads
- total reads
- % total reads
- % reads filtered for reads <1%
- % reads filtered for reads <10%
- alignment query (allele sequence)
- alignment midline
- alignment hit (reference)
- TF lost
- TF gained
- TF lost strand (+/-)
- TF gained strand (+/-)
- Lost TFBS sequence (in reference at this position, lost in allele) *Note: this TFBS sequence is in the reference, 5'-3' on strand indicated in 'strand'
- Gained TFBS sequence (not in reference at this position, novel to allele) *Note: this TFBS sequence is in the allele, 5'-3' on strand indicated in 'strand'
- Lost TFBS start coordinate (in reference)
- Lost TFBS end coordinate (in reference)
- Gained TFBS coordinate start (in allele)
- Gained TFBS coordinate end (in allele)
- Lost TFBS p-val (in reference)
- Gained TFBS p-val (in allele)
- comment
    - this field notes whether the ranked allele detailed in the row is below 35 bp, potentially consistent with primer dimer in the sequenced reads *("note: inferred allele length <=50 bp; check read in fasta file if warranted, or pre-process fastq reads")*

- Worksheet 7 (*7 "TF of interest", curated samples*)
  Synopsis: data table of all alleles for samples with at least one allele that has lost TFBSs for user-specified TF of interest, without coincident (positionally overlapping) regain of distinct TFBS for TF of interest, or gain of TFBS for different TF)—encompassing all alleles, for all samples that have at least one allele with lost TFBS for TF of interest
  Rows in worksheet 7 contain data for every ranked allele for samples having at least one allele with a lost TFBS **for a user-specified TF of interest** (without 'regain' or 'gain'; *see definitions of 'gain' and 'regain' above for worksheet 3).* The primary utility of worksheet 7 is to provide a spreadsheet collection of all alleles for samples having at least one allele that has lost a TFBS for the TF of interest *without coinciding substitution of this*

TFBS with a TFBS the TF of interest or for an entirely distinct TF, enabling sorting based on sample and allele rank columns to quickly deduce whether a sample may have TFBS losses for the TF of interest in some or all primary alleles (defined by high rank, *e.g.,* 1-2, based on high relative frequency), and whether the TFBS losses for the TF of interest are 'exclusive' losses (no 'regain' or 'gain'), or whether they are associated with regain of a different TFBS for the same TF, or gain of a new TFBS for a different TF. The following data are detailed in columns:

- sample
- allele rank (1 – up to 5)
- allele ID (fasta defline for ranked allele, including sample name derived from original fastq file name input as sample reads source to CollatedMotifs.py and read count parameters such as reads/total reads, allele rank, % total reads, percentile, % top 5 reads, % reads filtered to exclude reads occurring at <1% frequency, % reads filtered to exclude reads occurring at <10% frequency)
- reads
- total reads
- % total reads
- % reads filtered for reads <1%
- % reads filtered for reads <10%
- alignment query (allele sequence)
- alignment midline
- alignment hit (reference)
- TF lost (lost TFBS, no predicted regain of related TFBS for same TF, or gain of novel TFBS for distinct TF)
- TFBS for TF exclusively lost
    - 'x' means this is true (blank means not true) for allele in indicated row
- TFBS for TF lost with regain of different TFBS for same TF
    - 'x' means this is true (blank means not true) for allele in indicated row
- TFBS for TF lost with gain of TFBS for different TF
    - 'x' means this is true (blank means not true) for allele in indicated row
- TFBS for TF unchanged relative to reference
    - 'x' means this is true (blank means not true) for allele in indicated row
- TF lost strand (+/- or 'n/a')
- Lost TFBS sequence (in reference at this position, lost in allele) *Note: this TFBS sequence is in the reference, 5'-3' on strand indicated in 'strand'
- Lost TFBS start coordinate (in reference)
- Lost TFBS end coordinate (in reference)
- Lost TFBS p-val (in reference)
- comment
    - this field notes whether the ranked allele detailed in the row is below 35 bp, potentially consistent with primer dimer in the sequenced reads (*"note: inferred allele length <=50 bp; check read in fasta file if warranted, or pre-process fastq reads"*)
- genotype inference

        - this field assigns a simple genotype prediction (presuming diploidy among high-ranking alleles); for more complex genotypes (*e.g.*, if non-diploid genotypes are expected), consult Genotypes.py outputs

Worksheet 8: record of all TFBSs identified by FIMO in all alleles (no lost/gained annotation)

- Worksheet 8 (*8 All TFBS in alleles*)
  Synopsis: full data table of all TFBSs identified by FIMO (no filtering for lost or gained relative to reference sequence)—encompassing all alleles, all samples
  Rows in worksheet 8 contain data for every TFBS identified for FIMO, for every ranked allele for all samples. The primary utility of worksheet 8 is to provide a spreadsheet collection of all TFBSs identified by FIMO based on TF positional frequency matrices provided to CollatedMotifs.py (without filtering or interpretation as lost or gained relative to a reference sequence). These data are also available in the FIMO subdirectory *fimo_out*, but are compiled here for easy sorting and evaluation for samples and their alleles. The following data are detailed in columns:
    - Sample
    - allele rank (1 – up to 5)
    - allele ID (fasta defline for ranked allele, including sample name derived from original fastq file name input as sample reads source to CollatedMotifs.py and read count parameters such as reads/total reads, allele rank, % total reads, percentile, % top 5 reads, % reads filtered to exclude reads occurring at <1% frequency, % reads filtered to exclude reads occurring at <10% frequency)
    - reads
    - total reads
    - % total reads
    - % reads filtered for reads <1%
    - % reads filtered for reads <10%
    - alignment query (allele sequence)
    - alignment midline
    - alignment hit (reference)
    - TF
    - strand (+/-)
    - TFBS
    - TFBS start coordinate (in allele)
    - TFBS end coordinate (in allele)
    - TFBS p-val
    - comment
        - this field notes whether the ranked allele detailed in the row is below 35 bp, potentially consistent with primer dimer in the sequenced reads (*"note: inferred allele length <=50 bp; check read in fasta file if warranted, or pre-process fastq reads"*)

(d) ***script_metrics.txt*** logs script operation metadata, including operating system information, user-entered variables, fastq file information, fastq files processed, reference sequence(s) provided in fasta_ref file, file output information (output files, sizes), and script operation times.

```
CollatedMotifs.py: Script Metrics
Date: 07/20/2021

Operating system information:
    name: Kirks-MBP.attlocal.net
    platform: Darwin-17.7.0-x86_64-i386-64bit
    RAM (GB): 16.0
    physical CPU/effective CPU: 4/8
    executable: /Library/Frameworks/Python.framework/Versions/3.7/Resources/Python.app/Contents/MacOS/Python

User-entered variables:
    output_directory: /Users/kirkehmsen/Documents/CollatedMotifsOutput
    fastq_directory: /Users/kirkehmsen/Documents/Zenodo/ExampleTestFiles/CollatedMotifs_testfiles/fastq_files_subset
    fasta_ref: /Users/kirkehmsen/Documents/Zenodo/ExampleTestFiles/CollatedMotifs_testfiles/FKBP5_GOR+86.848kb_KE4.txt
    blastn_path: /Users/kirkehmsen/anaconda3/bin/blastn
    makeblastdb_path: /Users/kirkehmsen/anaconda3/bin/makeblastdb
    db_prefix: FKBP5_GOR+86.848kb
    fimo_path: /Users/kirkehmsen/Meme/bin/fimo
    fimo_motifs_path: /Users/kirkehmsen/Documents/Zenodo/ExampleTestFiles/CollatedMotifs_testfiles/JASPAR_CORE_2016_vertebrates.meme
    fasta_get_markov_path: /Users/kirkehmsen/Meme/meme-5.0.1/src/fasta-get-markov
    markov_background_file: /Users/kirkehmsen/Documents/Zenodo/ExampleTestFiles/CollatedMotifs_testfiles/hg38.fa
    TF_of_interest: NR3C1

fastq file information:
    Illumina sequencing run ID(s): @M00582:216
    Number of fastq files processed: 8
    Size distribution of fastq files processed:
        total... 3 MB
        range... max: 0.58 MB; min: 0.05928 MB; median: 0.339 MB; mean +/- stdev: 0.329 +/- 0.206 MB
    Read distribution within fastq files to process:
        total... 7,592 reads
        range... max: 1667 reads; min: 171 reads; median: 979.0 reads; mean +/- stdev: 949.0 +/- 595.0 reads

fastq files processed (name, size (MB), reads):
    /Users/kirkehmsen/Documents/Zenodo/ExampleTestFiles/CollatedMotifs_testfiles/fastq_files_subset/KE4-1-C02_S1178_L001_R1_001.fastq,
0.05928, 171
    /Users/kirkehmsen/Documents/Zenodo/ExampleTestFiles/CollatedMotifs_testfiles/fastq_files_subset/KE4-1-C02_S1178_L001_R2_001.fastq,
0.05928, 171
    /Users/kirkehmsen/Documents/Zenodo/ExampleTestFiles/CollatedMotifs_testfiles/fastq_files_subset/KE4-2-A01_S1249_L001_R1_001.fastq,
0.20663, 596
    /Users/kirkehmsen/Documents/Zenodo/ExampleTestFiles/CollatedMotifs_testfiles/fastq_files_subset/KE4-2-A01_S1249_L001_R2_001.fastq,
0.20663, 596
    /Users/kirkehmsen/Documents/Zenodo/ExampleTestFiles/CollatedMotifs_testfiles/fastq_files_subset/KE4-4-G02_S1514_L001_R1_001.fastq,
0.57792, 1667
    /Users/kirkehmsen/Documents/Zenodo/ExampleTestFiles/CollatedMotifs_testfiles/fastq_files_subset/KE4-4-G02_S1514_L001_R2_001.fastq,
0.57792, 1667
    /Users/kirkehmsen/Documents/Zenodo/ExampleTestFiles/CollatedMotifs_testfiles/fastq_files_subset/KE4-4-G10_S1522_L001_R1_001.fastq,
0.47218, 1362
    /Users/kirkehmsen/Documents/Zenodo/ExampleTestFiles/CollatedMotifs_testfiles/fastq_files_subset/KE4-4-G10_S1522_L001_R2_001.fastq,
0.47218, 1362

Reference sequences provided in fasta_ref file:
    >KE4

ACTTAAACTGGAGCTCTGACTTATTGTTCTCTTACTGCCCTAGAGCAATTTTGTTTTGAAGAGCACAGAACACCCTGTTCTGAATGTGGCTGGCACATGAACTCGATGTGCTGACAGCAATTTGTACTCCGATTAAAATAG
GGGGGAAAAAAGGAAAGAGAGTGCACAGCAGTAAC

# of TFBS motifs examined: 519
Identities of TFBS motifs examined:
    ALX3      ARNT::HIF1A     ATF4         ATF7      Ahr::Arnt       Alx1          Alx4           Ar
   Arid3a       Arid3b       Arid5a        Arnt        Arntl          Arx         Ascl2         Atf1
    Atf3         Atoh1       BARHL2        BARX1        BATF3      BATF::JUN      BCL6B        BHLHE22
   BHLHE23      BHLHE40      BHLHE41        BSX      Bach1::Mafk     Barhl1         Bcl6         Bhlha15
    CDX1         CDX2         CEBPA        CEBPB        CEBPD        CEBPE         CEBPG         CENPB
    CLOCK        CREB1        CREB3       CREB3L1        CTCF         CUX1          CUX2         Creb3l2
    Creb5        Crem          Crx          DBP         DLX6         DMRT3         DUX4          DUXA
Ddit3::Cebpa     Dlx1         Dlx2         Dlx3         Dlx4         Dmbx1          Dux           E2F1
    E2F2         E2F3         E2F4         E2F6         E2F7         E2F8          EBF1          EGR1
    EGR2         EGR3         EGR4          EHF         ELF1         ELF3          ELF4          ELF5
    ELK1         ELK3         ELK4         EMX1         EMX2          EN1           EN2          EOMES
     ERF          ERG         ESR1         ESR2         ESRRB        ESX1          ETS1          ETV1
    ETV2         ETV3         ETV4         ETV5         ETV6         EVX1          EVX2        EWSR1-FLI1
    Esrra        Esrrg         FEV         FIGLA        FLI1          FOS         FOS::JUN        FOSL1
    FOSL2        FOXA1        FOXB1        FOXC1        FOXC2        FOXD1         FOXD2         FOXF2
    FOXG1        FOXH1        FOXI1        FOXL1        FOXO3        FOXO4         FOXO6         FOXP1
    FOXP2        FOXP3        Foxa2        Foxd3        Foxj2        Foxj3         Foxk1         Foxo1
    Foxq1     GATA1::TAL1     GATA2        GATA3        GATA5        GBX1          GBX2          GCM1
    GCM2         GLI2         GLIS1        GLIS2        GLIS3        GMEB2         GRHL1          GSC
    GSC2         GSX1         GSX2         Gabpa        Gata1        Gata4         Gfi1          Gfi1b
    Gmeb1        HES5         HES7         HESX1        HEY1         HEY2          HIC2          HINFP
     HLF         HLTF        HMBOX1        HNF1A        HNF1B        HNF4G        HOXA10        HOXA13
    HOXA2        HOXA5        HOXB13       HOXB2        HOXB3       HOXC10        HOXC11        HOXC12
   HOXC13       HOXD11       HOXD12       HOXD13        HSF1         HSF2          HSF4       Hand1::Tcf3
    Hes1         Hes2         Hic1         Hmx1         Hmx2         Hmx3          Hnf4a         Hoxa11
```

Hoxa9	Hoxb5	Hoxc9	Hoxd3	Hoxd8	Hoxd9	ID4	INSM1
IRF1	IRF2	IRF7	IRF8	IRF9	ISL2	ISX	Id2
JDP2	JDP2(var.2)	JUN	JUN(var.2)	JUNB	JUND	JUND(var.2)	KLF13
KLF14	KLF16	KLF5	Klf1	Klf12	Klf4	LBX1	LBX2
LEF1	LHX2	LHX6	LHX9	LIN54	LMX1A	LMX1B	Lhx3
Lhx4	Lhx8	MAF::NFE2	MAFF	MAFG	MAFG::NFE2L1	MAFK	MAX
MAX::MYC	MEF2A	MEF2B	MEF2C	MEF2D	MEIS1	MEIS2	MEIS3
MEOX1	MEOX2	MGA	MIXL1	MLX	MLXIPL	MNT	MNX1
MSC	MSX1	MSX2	MTF1	MYBL1	MYBL2	MYF6	MZF1
MZF1(var.2)	Mafb	Mecom	Mitf	Mlxip	Msx3	Myb	Myc
Mycn	Myod1	Myog	NEUROD2	NEUROG2	NFAT5	NFATC1	NFATC2
NFATC3	NFE2	NFIA	NFIC	NFIC::TLX1	NFIL3	NFIX	NFKB1
NFKB2	NFYA	NFYB	NHLH1	NKX2-3	NKX2-8	NKX3-2	NKX6-1
NKX6-2	NOTO	NR1H2::RXRA	NR2C2	NR2F1	NR3C1	NR3C2	NR4A2
NRF1	NRL	Neurog1	Nfe2l2	Nkx2-5	Nkx2-5(var.2)	Nkx3-1	Nobox
Npas2	Nr1h3::Rxra	Nr2e1	Nr2e3	Nr2f6	Nr2f6(var.2)	Nr5a2	OLIG1
OLIG2	OLIG3	ONECUT1	ONECUT2	ONECUT3	OTX1	OTX2	PAX1
PAX3	PAX4	PAX5	PAX7	PAX9	PBX1	PDX1	PHOX2A
PITX3	PKNOX1	PKNOX2	PLAG1	POU1F1	POU2F1	POU2F2	POU3F1
POU3F2	POU3F3	POU3F4	POU4F1	POU4F2	POU4F3	POU5F1B	POU6F1
POU6F2	PPARG	PRDM1	PROP1	PROX1	PRRX1	Pax2	Pax6
Phox2b	Pitx1	Pou2f3	Pou5f1::Sox2	Pparg::Rxra	Prrx2	RARA	RARA(var.2)
RARA::RXRA	RAX	RAX2	REL	RELA	REST	RFX2	RFX3
RFX4	RFX5	RHOXF1	RORA	RORA(var.2)	RREB1	RUNX1	RUNX2
RUNX3	RXRA::VDR	RXRB	RXRG	Rarb	Rarb(var.2)	Rarg	Rarg(var.2)
Rfx1	Rhox11	Rxra	SCRT1	SCRT2	SHOX	SMAD2::SMAD3::SMAD4	SMAD3
SNAI2	SOX10	SOX21	SOX4	SOX8	SOX9	SP1	SP2
SP3	SP4	SP8	SPDEF	SPI1	SPIB	SPIC	SREBF1
SREBF2	SREBF2(var.2)	SRF	SRY	STAT1	STAT1::STAT2	STAT3	Shox2
Six3	Sox1	Sox11	Sox17	Sox2	Sox3	Sox5	Sox6
Spz1	Srebf1(var.2)	Stat4	Stat5a::Stat5b	Stat6	T	TAL1::TCF3	TBP
TBR1	TBX1	TBX15	TBX19	TBX2	TBX20	TBX21	TBX4
TBX5	TCF3	TCF4	TCF7L2	TEAD1	TEAD3	TEAD4	TEF
TFAP2A	TFAP2A(var.2)	TFAP2A(var.3)	TFAP2B	TFAP2B(var.2)	TFAP2B(var.3)	TFAP2C	TFAP2C(var.2)
TFAP2C(var.3)	TFAP4	TFCP2	TFE3	TFEB	TFEC	TGIF1	TGIF2
THAP1	TP53	TP63	TP73	Tcf12	Tcf21	Tcf7	Tcfl5
Twist2	UNCX	USF1	USF2	VAX1	VAX2	VENTX	VSX1
VSX2	Vdr	XBP1	YY1	YY2	ZBED1	ZBTB18	ZBTB33
ZBTB7A	ZBTB7B	ZBTB7C	ZEB1	ZIC1	ZIC3	ZIC4	ZNF143
ZNF263	ZNF354C	ZNF410	ZNF740	Zfx	Znf423	mix-a	

Record of ranked alleles deprecated from analysis output:

No hits identified by BLASTN in alignment database:
KE4-1-C02_R1+R2_[3/144]_rank3_%totalreads:2.08_percentile:96_%top5reads:3.16_%readsfilteredfor1%:3.23_%readsfilteredfor10%:None
KE4-1-C02_R1+R2_[1/144]_rank4_%totalreads:0.69_percentile:48_%top5reads:1.05_%readsfilteredfor1%:None_%readsfilteredfor10%:None
KE4-4-G10_R1+R2_[4/806]_rank5_%totalreads:0.5_percentile:97_%top5reads:0.59_%readsfilteredfor1%:None_%readsfilteredfor10%:None

Multiple hits identified by BLASTN in alignment database:
None

>1 high-scoring pair (hsp) identified by BLASTN, and hsp's were reconstructed into a hypothesized allele:
KE4-1-C02_R1+R2_[82/144]_rank1_%totalreads:56.94_percentile:100_%top5reads:86.32_%readsfilteredfor1%:88.17_%readsfilteredfor10%:100.0

>1 high-scoring pair (hsp) identified by BLASTN, but hsp's could not be reconstructed into a hypothesized allele:
KE4-1-C02_R1+R2_[1/144]_rank5_%totalreads:0.69_percentile:48_%top5reads:1.05_%readsfilteredfor1%:None_%readsfilteredfor10%:None

File output information:
Output directory: /Users/kirkehmsen/Documents/CollatedMotifsOutput
Total file #: 6
Total file output sizes: 352.0 bytes
07212021_collated_TFBS.txt: 33.5 KB
07212021_markov_background.txt: 274.0 bytes
07212021_blastn_alignments.txt: 40.4 KB
07212021_collated_TFBS.xlsx: 76.4 KB
07212021_script_metrics.txt: 11.4 KB
07212021_fasta.fa: 5.4 KB

Script operation times:
start time: 22:30:43
makeblastdb and fasta-get-markov processing time: 0 hr|00 min|17 sec|497406 microsec
fasta processing time: 0 hr|00 min|00 sec|410666 microsec
alignments processing time: 0 hr|00 min|00 sec|462618 microsec
allele definitions processing time: 0 hr|00 min|00 sec|186646 microsec
TFBS processing time (FIMO): 0 hr|00 min|04 sec|599773 microsec
TFBS collation processing time: 0 hr|00 min|04 sec|572136 microsec
total processing time: 0 hr|00 min|27 sec|876946 microsec
end time: 22:31:11

Supplementary Figure 11.

(a, b) ***FKBP5* GOR conservation across 100 vertebrates:** PhyloP (blue) and phastCons (green) tracks (12) for *FKBP5* (a) intronic and (b) upstream GORs defined in A549, viewed in the UCSC Genome Browser (13). GBS matches identified by RSAT matrix-scan ((14) or by 'short match' to generic GBS motif 5'-NNN**ACA**NNNN**GT**N**C**NN-3' in UCSC Genome Browser are indicated.

(c, d) **Topological units, genes, and GORs in the vicinity of dex-responsive *FKBP5:*** a 1.5 Mb region flanking *FKBP5* (c) is largely devoid of GORs, outside of the GORs contained with the ~400 kb topologically associated domain (TAD) encompassing dex-responsive *FKBP5* (d). *FKBP5* is the sole gene in this unit that is responsive to dex (fold-change >1.5 based on RNA-seq, 100 nM dex, 4 h; Lindsey Pack & Keith Yamamoto, unpublished). Intrachromosomal *topological contacts* between genomic regions (5 kb resolution, orange brackets) are displayed as a custom tabix track (15) generated from A549 Hi-C data (100 nM dex, 0 h) publicly available at the NCBI Gene Expression Omnibus (accession number: GSE92819 (16)).

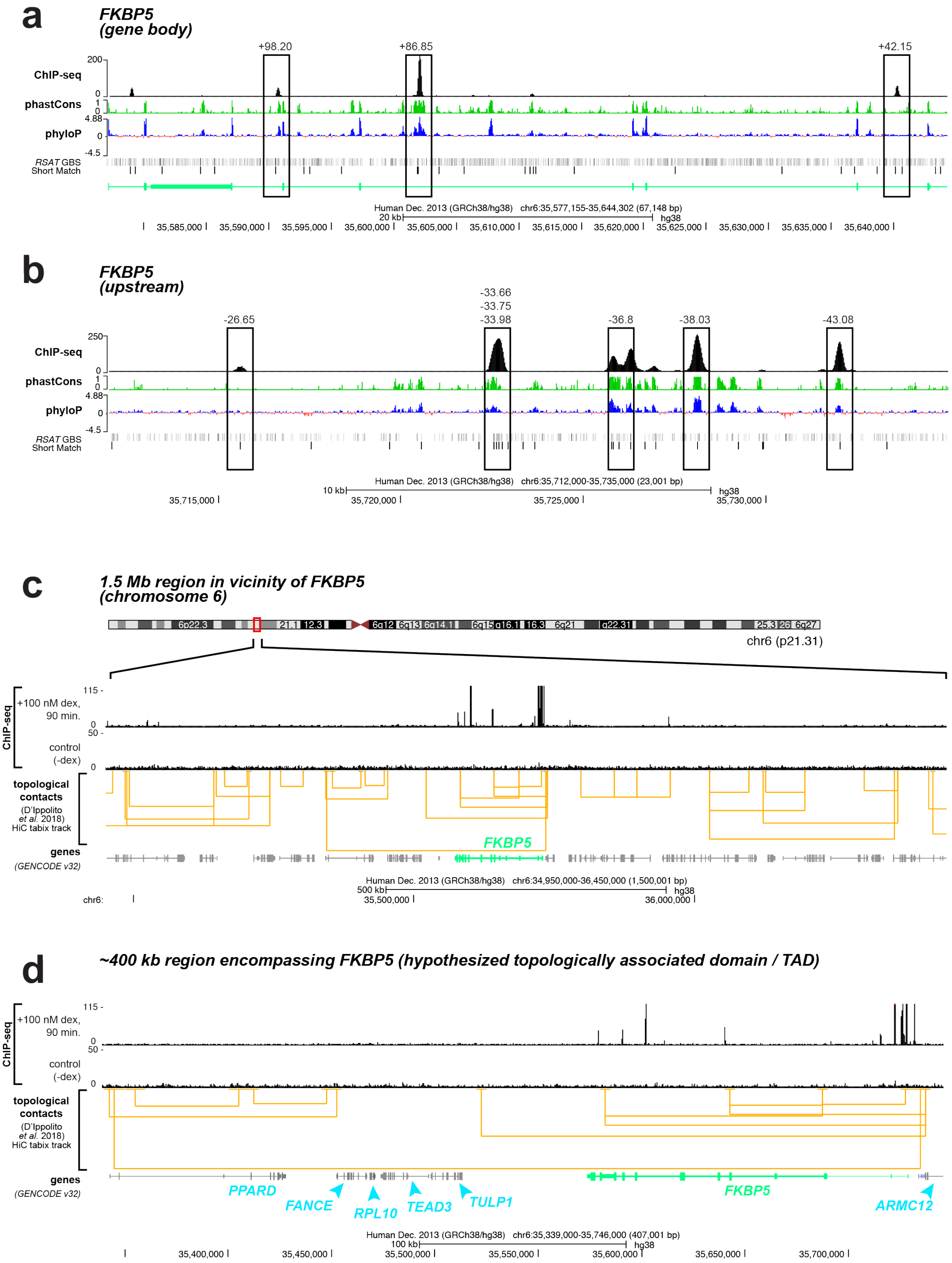
a
FKBP5
(gene body)
+98.20
+86.85
+42.15
ChIP-seq
200
0
phastCons
1
0
4.88
phyloP
0
-4.5
RSAT GBS
Short Match
Human Dec. 2013 (GRCh38/hg38) chr6:35,577,155-35,644,302 (67,148 bp)
20 kb
hg38
35,585,000
35,590,000
35,595,000
35,600,000
35,605,000
35,610,000
35,615,000
35,620,000
35,625,000
35,630,000
35,635,000
35,640,000
b
FKBP5
(upstream)
-33.66
-33.75
-33.98
-26.65
-36.8
-38.03
-43.08
ChIP-seq
250
0
phastCons
1
0
4.88
phyloP
0
-4.5
RSAT GBS
Short Match
Human Dec. 2013 (GRCh38/hg38) chr6:35,712,000-35,735,000 (23,001 bp)
10 kb
hg38
35,715,000
35,720,000
35,725,000
35,730,000
c
1.5 Mb region in vicinity of FKBP5
(chromosome 6)
6p22.3
21.1
12.3
6q12
6q13
6q14.1
6q15
q16.1
16.3
6q21
q22.31
25.3
26
6q27
chr6 (p21.31)
ChIP-seq
115 -
+100 nM dex,
90 min.
0
50 -
control
(-dex)
0
topological
contacts
(D'Ippolito
et al. 2018)
HiC tabix track
FKBP5
genes
(GENCODE v32)
Human Dec. 2013 (GRCh38/hg38) chr6:34,950,000-36,450,000 (1,500,001 bp)
500 kb
hg38
chr6:
35,500,000
36,000,000
d
~400 kb region encompassing FKBP5 (hypothesized topologically associated domain / TAD)
ChIP-seq
115 -
+100 nM dex,
90 min.
0
50 -
control
(-dex)
0
topological
contacts
(D'Ippolito
et al. 2018)
HiC tabix track
genes
(GENCODE v32)
PPARD
FANCE
RPL10
TEAD3
TULP1
FKBP5
ARMC12
Human Dec. 2013 (GRCh38/hg38) chr6:35,339,000-35,746,000 (407,001 bp)
100 kb
hg38
35,400,000
35,450,000
35,500,000
35,550,000
35,600,000
35,650,000
35,700,000

Supplementary Figure S12. BaseSpace Sequencing Hub metadata for files processed in use case.

Screenshots of metadata preserved in BaseSpace Sequencing Hub, https://basespace.illumina.com/run/36555528/171004-M00582-0217-000000000-AV3NW. 9,216 fastq files were demultiplexed from sequencing RunID: 171004_M00582_0217_000000000-AV3NW (10-04-2017).

General Info

Run Status	Complete
Lane QC Status	QC Passed
Flowcell ID	000000000-AV3NW
Run ID	171004_M00582_0217_000000000-AV3NW
Instrument Name	M00582
Instrument Type	
%PF	MiSeq
Avg %Q30	92.14%
Yield	88.45%
Cycles	2.53242 bp
Created	151 | 8 | 8 | 151
Owner	2017-10-04 11:32
User	Kirk Ehmsen
File Count/Size	Kirk Ehmsen

Per Lane Metrics

Columns

LANE	READ	CLUSTER PF (%)	%≥Q30	YIELD	ERROR RATE%	READS PF	DENSITY	TILES	LEGACY PHAS/PREPHAS (%)	INTENSITY	COMMENTS	STATUS
1	1	92.15 ±1.95	93.93	1.21 Gbp	0.62 ±0.10	8,065,046	449 ±11	28	0.155 / 0.396	121 ±12		QC Passed
	2(I)		75.65	56.46 Mbp	0.00 ±0.00				0.000 / 0.000	343 ±43		
	3(I)		95.51	56.46 Mbp	0.00 ±0.00				0.000 / 0.000	317 ±40		
	4		83.24	1.21 Gbp	0.73 ±0.08				0.250 / 0.373	91 ±11		

Per Lane Metrics

Columns

LANE	READ	CLUSTER PF (%)	%≥Q30	YIELD	ERROR RATE%	READS PF	DENSITY	TILES	LEGACY PHAS/PREPHAS (%)	INTENSITY	COMMENTS	STATUS
1	1	92.15 ±1.95	93.93	1.21 Gbp	0.62 ±0.10	8,065,046	449 ±11	28	0.155 / 0.396	121 ±12		QC Passed
	2(I)		75.65	56.46 Mbp	0.00 ±0.00				0.000 / 0.000	343 ±43		
	3(I)		95.51	56.46 Mbp	0.00 ±0.00				0.000 / 0.000	317 ±40		
	4		83.24	1.21 Gbp	0.73 ±0.08				0.250 / 0.373	91 ±11		

TOTAL READS	PF READS	% READS IDENTIFIED (PF)	CV	MIN	MAX
17,505,188	16,130,092	75.3308	0.6960	0.0000	0.0332

Supplementary Figure S13. Barcode distributions among sequenced reads.

Among 9,216 barcode pairs used to uniquely label amplicons, 99.4% (9,164) were identified as linked to reads by sequencing on an Illumina® MiSeq instrument. Barcode pairs not identified (52, <0.6%) are suspected to have been assigned to wells with insufficient or no template source.

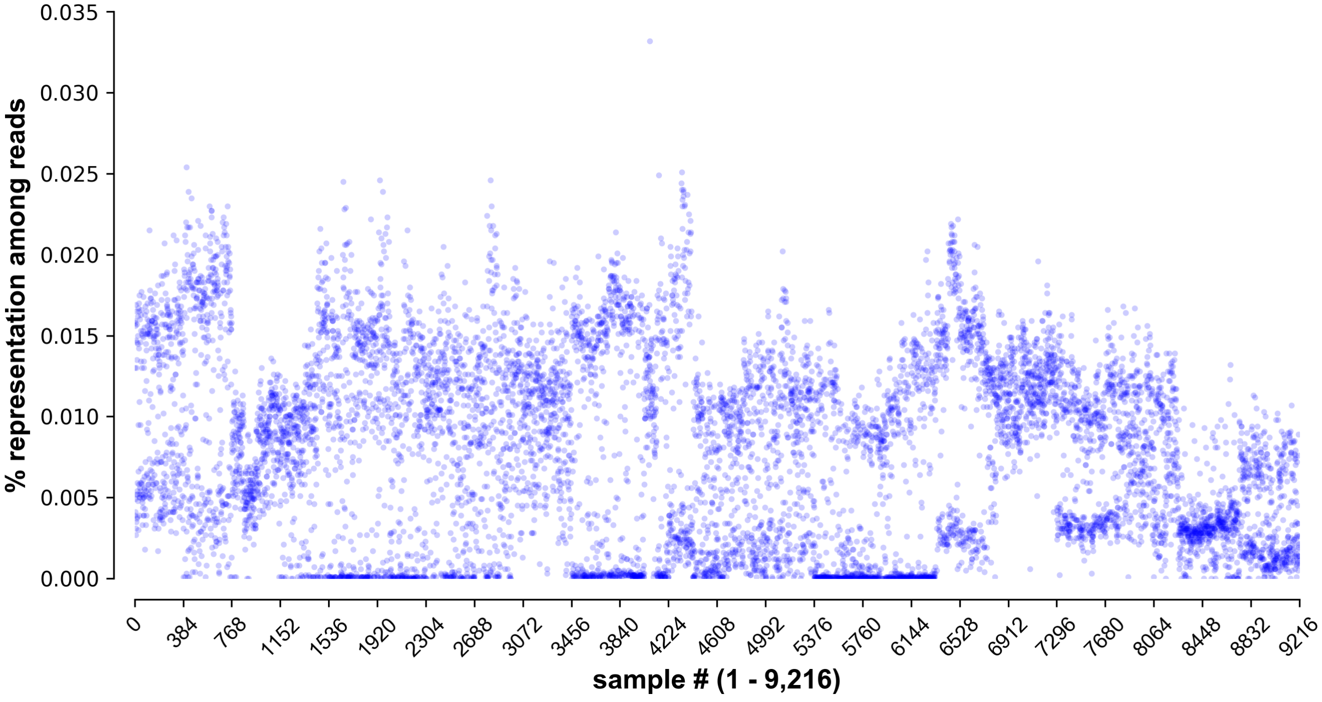

Supplementary Figure S14. GOR +*86.85 kb* reporter activity & *clone #1b* genotype.

(a) Luciferase reporter activity (*left*) of GOR +*86.85 kb*. 500-bp region flanking GBS (highlighted yellow, *right*) was PCR-amplified from A549 genomic DNA, cloned into pGL4.10 with minimal promoter E4TATA driving *Photinus* firefly luciferase (17), and transfected into A549 cells in 24-well plates with plasmid expressing *Renilla* luciferase (Promega) as signal normalization control (Lipofectamine 2000, ThermoFisher) (plasmid pKE439/pGL4.10-E4TATA-FKBP5_GOR+86.85kb: AddGene 173920; negative control (E4TATA): AddGene 173919). After 24 h, media was changed to DMEM with 5% charcoal-stripped FBS; 2 h later, cells were treated with 5X dexamethasone (Sigma) in ethanol to the indicated concentrations (0.01, 1, or 100 nM, or ethanol control) for 4 h. Cells were harvested using Passive Lysis Buffer (Promega) and luminescence was measured in a Veritas dual-injection luminometer (Turner BioSystems) after delivery of luciferin (*Photinus,* NanoLight Technology), quench, and delivery of coelenterazine (*Renilla,* NanoLight Technology). GOR -*26.65 kb* was not cloned for evaluation in reporter assay.

Genotype of 6-5 F03 (*clone #1b*): (b) agarose gel (2%) demonstrating two alleles (large deletions); M, marker = Quick-Load 100 bp DNA ladder, NEB; (c) allele sequences. Genotypes of ΔΔ+*86.85 kb* mutant 4-4 G02 (*clone #1a*) and ΔΔ-*26.65 kb* mutants 11-1 D06 (*clone #2a*) and 11-5 G06 (*clone #2b*) are shown in Fig. 5.

a

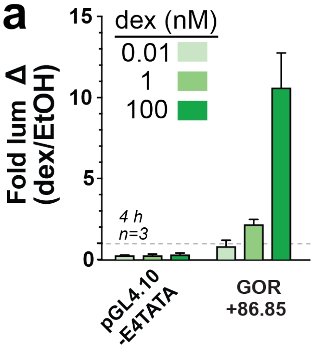

TAGAGAAAGAAATAAACAAGTTAAAAAGAGACAACATTTTTTTCTTGACT
TAAAGACTATGTAACTTAAAGGGGGAGGGAGGTCCTCCAAATCCCCACAA
AGTCAAACCAAACCAAATTACCCAAGCTTAGCTGCGCAATCGGAGTGTAA
CCACATCAAGCGAGCTGCAAAACATCACTTAAACTGGAGCTCTGACTTAT
TGTTCTCTTACTGCCCTAGAGCAATTTTGTTTTGAAGAGCACAGAACACC
CTGTTCTGAATGTGGCTGGCACATGAACTCGATGTGCTGACAGCAATTTG
TACTCCGATTAAAATAGGGGGGAAAAAAGGAAAGAGAGTGCACAGCAGTA
ACAAAAATGAAATGCAAGAACCCCTAAACCATGCAATTTGTATTTTAGGA
AGCAAAACTGAGAAAGAGGCTCCATAACTTAAAAAACAAAAAACAAAAAG
CTGTAAATGCTCCAGCCTAAAGACATTAGCTCTGGTAAGTGTCTATTTTT

b

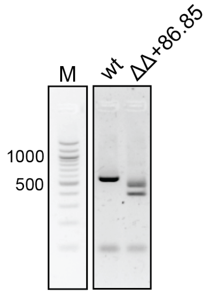

c

allele 1 Δ 84 bp

GCTGCGCAATCGGAGTGTAACCACAT-AAGCGAGCTGCAAAACATCACTT
GCTGCGCAATCGGAGTGTAACCACATCAAGCGAGCTGCAAAACATCACTT

AAACTGGAGCTCTGACTTATTGTTCTCTTACTGCCCTAGAGCAATTTTGT
AAACTGGAGCTCTGACTTATTGTTCTCTTACTGCCCTAGAGCAATTTTGT

TT--
TTTGAAGAGCACAGAACACCCTGTTCTGAATGTGGCTGGCACATGAACTC

------------------------------------GGGGGGAAAAAAGG
GATGTGCTGACAGCAATTTGTACTCCGATTAAAATAGGGGGGAAAAAAGG

allele 2 Δ 160 bp

GCTGCGCAATCGGAGTGTAACCACAT------------------------
GCTGCGCAATCGGAGTGTAACCACATCAAGCGAGCTGCAAAACATCACTT

AAACTGGAGCTCTGACTTATTGTTCTCTTACTGCCCTAGAGCAATTTTGT

TTTGAAGAGCACAGAACACCCTGTTCTGAATGTGGCTGGCACATGAACTC

------------------------------------GGGGGGAAAAAAGG
GATGTGCTGACAGCAATTTGTACTCCGATTAAAATAGGGGGGAAAAAAGG

Supplementary Figure S15. Percentage of edited alleles with GBS lost that reconstitute ('regain') a new GBS.

For five *FKBP5* GORs (canvased by fourteen distinct guide RNAs), the frequency of GBS 'side-steps' (native GBS sequence lost, but novel alternative GBS sequence conferred by indel) ranged from 14-60% of altered alleles. Vertical bar in each horizontal plot represents median % of alleles that lost native GBS (left) or % of these alleles that nevertheless reconstituted a novel GBS (right) for guides sampled across indicated GOR; white box is mean values across sampled guide(s) targeting the indicated GOR.

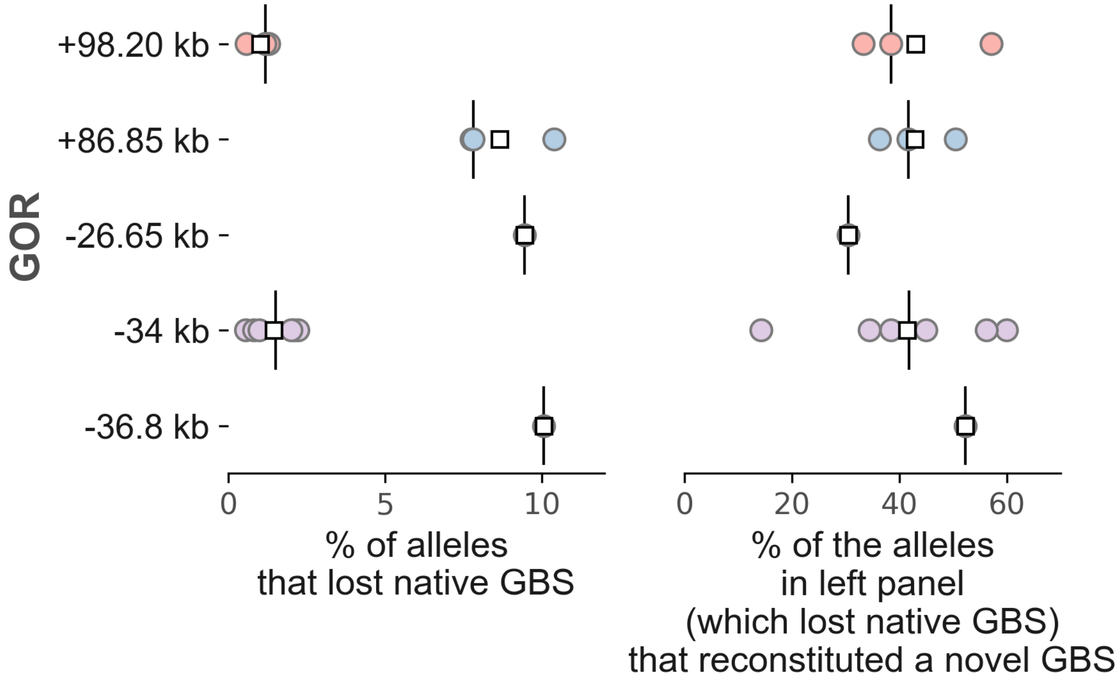

Supplementary Methods

GR ChIP-seq data availability—GR ChIP-seq datasets were collected under control (EtOH) and GR-activating (100 nM dexamethasone) conditions (90 min treatment). Raw and processed ChIP-seq datasets are available from the NCBI Gene Expression Omnibus (accession number: GSE163398); materials, methods, and analyses will be reported separately (Wissink, Martinez, *et al.*, in preparation).

sgRNA cloning into pSpCas9(BB)-2A-Puro vectors—20-nt single guide (sg) RNA sequences (18) were designed using online tools: CRISPR-MIT (crispr.mit.edu) and sgRNA Designer (19) (https://portals.broadinstitute.org/gpp/public/analysis-tools/sgrna-design). Guides were selected with predicted incision position within or near to targeted GBS motifs underlying GOR peak summits, and prioritized based on predicted on-target/off-target (CRISPR-MIT) and efficiency scores (sgRNA Designer). Oligo sequences for sgRNA duplexes with short single-stranded DNA overhangs (compatible with *Bbs*I cloning) were designed using the shell script *'sgRNA_BbsI_oligo_conversions.sh'*

(https://github.com/YamamotoLabUCSF/sgRNA_BbsI_oligo_conversions.sh),

which appends 'CACCG' to the 5' end of the sgRNA DNA sequence ('fwd'), and generates a reverse complement with 'AAAC' appended at its 5' and 'C' appended at its 3' end ('rev'). Oligos (unphosphorylated) were ordered from Integrated DNA Technologies (IDT, Coralville, IA). Oligos ('gRNA top' and 'gRNA bottom') were phosphorylated and annealed by mixing 1 µL 'top' (100 µM) and 1 µL 'bottom' (100 µM) oligos with 1 µL T4 PNK (New England Biolabs) and 1 µL T4 DNA ligase buffer in a 10 µL reaction volume, with incubation in a Peltier PTC-200 DNA Engine Thermal Cycler (37 °C for 30 min, 95 °C for 5 min, ramp to 25 °C at 0.1 °C/sec, 10 °C indefinitely). Phosphorylated, annealed sgRNA duplexes were diluted 1:20 with nuclease-free H_2O (Ambion).

1 µL 1:20 sgRNA duplex was mixed with 1 µL pSpCas9(BB)-2A-Puro (PX459) V2.0 (100 ng/µL, gift from Feng Zhang; Addgene plasmid #62988, http://n2t.net/addgene:62988, RRIS:Addgene_62988), 1 µL CutSmart buffer, 1 µL DTT (10 mM), 0.4 µL ATP (25 mM), 0.5 µL *Bbs*I, 0.5 µL T4 DNA ligase + nuclease-free H_2O to 20 µL. Vectors were assembled in a thermocycler ([37 °C for 5 min, 21 °C for 5 min], cycle 5x, 10 °C indefinitely). 1 µL ligation products were transformed into 8 µL competent *E. coli*, incubated on ice for 30 min, heat shocked for 30 sec, 42 °C, followed by incubation on ice for 5 min. 950 µL SOC was added, followed by incubation at 37 °C with shaking for 60 min; finally, 100 µL was spread onto LB+Amp plates for overnight growth and selection. Candidates were Sanger sequenced (QuintaraBio, South San Francisco, CA)

with oKE198 (U6_fwd, 5'- GAGGGCCTATTTCCCATGATTCC-3') to identify and confirm sgRNA cloned into *Bbs*I sites.

A549 transfection and puromycin selection—A549 cells (authenticated by short tandem repeat (STR) profiling (ATCC, Manassas, VA)) were plated in wells of a 24-well tissue culture plate (Corning Inc., Corning, NY) at $3x10^4$ cells/well in 500 µL DMEM/low glucose (HyClone Laboratories, Logan UT) containing 5% FBS (GemCell, Gemini Bio-Products, West Sacramento, CA), and maintained in an IncuSafe CO_2 incubator (Sanyo Scientific) at 37 °C. 24 h later, plasmids diluted to 100 ng/µL were transfected into plated cells under the following conditions: 500 ng plasmid was mixed in Opti-MEM Reduced Serum Media (ThermoFisher Scientific, Waltham, MA) in a final volume of 25 µL, then mixed with a 25 µL Opti-MEM volume containing 1 µL Lipofectamine 3000 (ThermoFisher); after 5 min incubation, the 50 µL transfection complex volume was delivered to plated cells (in total, twenty-five distinct Cas9 treatment combinations). After 24 h, puromycin selection was imposed at 0.61 µg/mL (2-day SF90 determined for A549) by replacing media with puromycin (InvivoGen, San Diego, CA) @ SF90. Four days later, single cells were sorted into wells of 96-well plates containing 100 µL HAM'S F-12 (Lonza, Basel, Switzerland)/10% FBS using a BD FACSAria2 (Center for Advanced Technology, UCSF), and cultured for >2 weeks with media changes until ready for genotyping. For each of twenty-five Cas9 treatment combinations, cells were sorted into 3-4 96-well plates (therefore sampling up to 288-384 clones (~1-1.3%) of each treatment population for genotypic evaluation), amounting to a total of ninety-six 96-well plates across Cas9 treatments. Following sequencing and genotypic analysis (*below*), selected clones were scaled up to 10-cm plates and aliquoted to 3 cryogenic vials for long-term storage in liquid N_2.

Assessment of amplicon length-dependent sequence recovery on MiSeq (Supp. Fig. 8b)—Seven amplicons ranging in size from 100-1500 bp (100, 175, 300, 500, 800, 1000, 1500) were PCR-amplified from pGL4.10 (Promega, GenBank AY738222); 100-1000 bp constructs were produced as stitched amplicons in two consecutive PCRs (A & B), such that all amplicons shared the same end sequences up to the maximum possible for each length: two fragments with engineered overlap were separately amplified in a PCR-1A, then stitched together as a single amplicon in a second PCR-1B. Full-length amplicons were isolated by agarose gel extraction (Qiagen QIAQuick Gel Extraction Kit), and re-amplified in a PCR-2 with primers to add extensions compatible with i5 and i7 barcode primers. Amplicon sets were uniquely barcoded in a PCR-3 as follows: amplicon set 'A1': all 7 amplicons barcoded with i5 A01; unique sizes independently barcoded with i7 A01-A07 (1500 bp-100 bp), amplicon set 'A3': all 7 amplicons barcoded with i5 A03; unique sizes independently barcoded with i7 A01-A07 (1500 bp-100 bp).

Amplicons were purified by DNA Clean & Concentrator-5 (Zymo Research, Irvine, CA), diluted to 5 pg/μL (approximated from NanoDrop spectrophotometry (ThermoFisher)), and each indexed amplicon was independently quantified by KAPA Library Quantification Kit for Illumina® Platforms (KK4844, KAPA Biosystems, Wilmington, MA) in duplicate, with six standards in triplicate. Amplicons were pooled in defined molar (amplicon) ratios as libraries (100 bp : 175 bp : 300 bp : 500 bp : 800 bp : 1000 bp : 1500 bp: 'A1'—1:1:1:1:1:1:1, 'A3'—1:10:50:100:200:500:1000). Libraries were diluted to 4 nM and prepared for sequencing using the MiSeq Nano Reagent Kit v2: PE, 2x150 bp (Illumina®, San Diego, CA), according to manufacturer instructions (final library concentration 7 pM, 10% each 'A1' and 'A3' library, 40% PhiX DNA spike-in (PhiX Control v3, Illumina®)). MiSeq cluster density was 1237 ± 1 k/mm^2, with 76.64% of reads passing filter @ %Q30=85.15 to yield 1,379,866 reads.

For 'A1' and 'A3' libraries, the top 15 read types for each amplicon were identified and quantified by bash, converted to fasta format (Python) and manually evaluated for alignment against amplicon sequences using the 'Align Multiple Sequences' function in SnapGene (GSL BioTech LLC, Chicago, IL). *% representation among total mapped reads* and *% reads that map as expected* were plotted in Python matplotlib as a function of amplicon size (bp) (**Supp. Fig. 8b**).

MiSeq primer design and T_m determination—To genotype cells in 96-well plates, media was removed from wells by aspiration, cells were washed with 100 μL DPBS/-calcium,-magnesium (HyClone), and cells were trypsinized in 30 μL trypsin-EDTA (Gibco); 15 μL trypsinized volume was lysed with an equal volume of 2X Lysis Buffer (2X: 100 mM KCl, 20 mM Tris-HCl, pH 8.3, 5 mM $MgCl_2$, 0.9% NP-40, 0.9% Tween-20 + Proteinase K (Roche, recombinant PCR-grade, 19 mg/mL) @1:100) in a thermocycler programmed at 65 °C for 30 min, 95 °C for 15 min. Primers to amplify Cas9-targeted loci were designed in SnapGene to span 175 bp flanking a targeted GBS with predicted T_m > 55 °C; 5' extensions to make PCR1 amplicons compatible with PCR2 amplification were added as described in **Supp. Fig. 5** (sequences available in **Supp. Excel file**). Primers were evaluated for empirical T_a in a thermocycler (95 °C for 10 min., [95 °C for 10 sec, 55→65 °C for 20 sec over 8 temperature increments (55, 55.8, 57.1, 59, 61.2, 63.1, 64.4, 65 °C), 72 °C for 15 sec], cycle 35x, 72 °C for 5 min., 12 °C indefinitely).

PCR1 & PCR2 for dual-indexed (i5 & i7) amplicon library preparation—4 μL lysed sample was used as template in 20 μL PCR1 volumes (1X HF Phusion buffer, 200 μM dNTP (each), 225 nM each primer, ~1 U Phusion pol). PCR1 was performed in a thermocycler (98 °C for 2.5 min, [98 °C for 30 sec, pre-determined T_a for 20 sec, 72 °C for 30 sec], cycle 30x total, 72 °C for 5 min, 12 °C indefinitely). 0.5 μL PCR1 was used as template in 20 μL PCR2 volumes (1X

HF Phusion buffer, 200 μM dNTP (each), 200 nM each i7/i5 primer, ~1 U Phusion pol). PCR2 primers (i7/i5) are described in **Supp. Fig. 1**. In some cases (larger anticipated deletions with two Cas9 constructs), amplicon products were visualized by agarose gel electrophoresis with SybrSafe (Invitrogen) on a Bio-Rad SubCell Model 192 (Bio-Rad Laboratories, Hercules, CA).

MiSeq deep sequencing—10 μL amplicons from each PCR2 well were pooled and cleaned by SPRISelect (Beckman Coulter, Brea, CA) @1:1 ratio (100 μL pooled amplicons + 100 μL SPRISelect beads). The pooled library was diluted to within range of standards provided with the KAPA Library Quantification Kit for Illumina® Platforms (KK4844, KAPA Biosystems, Wilmington, MA). qPCR quantification was performed as specified (6 μL 2X premix + 4 μL DNA standard or library, standards in 3 dilutions in triplicate (1:1,000,000, 1:10,000,000, 1:100,000,000), yielding estimated working concentration of 143.62 nM). Sample Sheet was prepared by a pilot version of *SampleSheet.py*, using 96 lines of plate:barcode assignments to populate 9,216 sample:barcode [Data] relationships as detailed in **ExampleTestFiles** (https://zenodo.org/record/3406861, DOI: **10.5281/zenodo.3406861**). The library was diluted to 4 nM and prepared for sequencing using the MiSeq Reagent Kit v2: PE, 2x150 bp (Illumina®, San Diego, CA), according to manufacturer instructions (10 pM with 10% PhiX DNA spike-in (PhiX Control v3, Illumina®)). MiSeq cluster density was 449 ± 11 k/mm^2, with 90% of reads passing filter to yield 8,065,046 reads (on average 875 reads/well).

Genotypic analysis—18,432 fastq files (representing R1 & R2 files for 9,216 demultiplexed barcode (sample)) were generated by MiSeq Controller Software onboard the MiSeq instrument. For genotype processing by *Genotypes.py,* fastq files were sorted to subdirectories by overarching sample ID (Cas9 treatment combination, *e.g.*, KE-1, KE-2, …KE-25) and submitted to the Jupyter Notebook script in batches.

TFBS analysis—As for genotype processing, TFBS collation by *CollatedMotifs.py* was performed for fastq files in sample batches.

Regulatory analysis (dex treatment, RNA isolation and RT-qPCR of ablated clones)—Cells revived from liquid N_2 storage were grown to near-confluency in 10-cm dishes, trypsinized and counted by hemocytometer, and diluted to 1.5 x 10^5 cells/mL. 3 mL diluted cells were transferred to each well of a 6-well tissue culture dish (Corning), and the entire plate was gently vortexed at speed setting 4-5 while loosely held flat on a 3-inch platform attachment of the Vortex Genie 2 (ThermoFisher), to evenly distribute cells in wells. Plates were transferred to a 37 °C incubator; 24 h later, media was aspirated and replaced with 2400 μL DMEM/5% FBS (charcoal/dextran-stripped, Omega Scientific, Tarzana, CA), and plates were returned to incubator for 3 h. During this time, dexamethasone (Sigma, St. Louis, MO) stock at 5 mM in

ethanol (Decon Koptec, King of Prussia, PA) was diluted to 1:100 and (serially) to 1:10,000 in ethanol. At 3 h time point, 1 μL each dex stock (or ethanol for control) was added to 10 mL media/charcoal-stripped FBS. 600 μL of appropriate treatment stock was added to appropriate wells, mixed gently by rocking, and plates were returned to incubator. 4 h later, media was aspirated; cells were washed with 3 mL PBS, lysed in 350 μL RLT buffer (Qiagen, Hilden, Germany) with β-mercaptoethanol (Bio-Rad Laboratories), transferred to 1.5 mL Eppendorfs, flash-frozen in liquid N_2, and stored at -80 °C until processing.

For RNA isolation, cell lysates were transferred to QIAShredder (Qiagen) and centrifuged at >12,000 *x*g, 2 min. 300 μL 70% ethanol was added to flow-through and the volume was transferred to an RNeasy Mini Kit tube and centrifuged at full speed, 30 sec. Flow-through was discarded and 350 μL Buffer RW1 (wash buffer) was added to the column, then centrifuged full speed, 30 sec. Flow-through was discarded. For each sample, 10 μL RNase-free DNase I (Qiagen) stock solution was added to 70 μL Buffer RDD, mixed, added directly to the RNeasy column, and incubated on benchtop for 15 min. 350 μL Buffer RW1 was added, followed by centrifugation, then 500 μL RPE followed by centrifugation, then 500 μL RPE with centrifugation for 2 min. RNA was eluted into a 1.5 mL collection tube in 30 μL RNase-free H_2O, and concentration was determined by NanoDrop (Thermofisher). RNA quality for all samples was verified on an Agilent 2100 Bioanalyzer (RNA 6000 Nano kit, Eukaryote Total RNA assay) with RNA Integrity Numbers (RINs) > 8 for all samples (mean = 9.7, median = 10, range 8.2-10).

In preparation for qPCR, a small volume of RNA sample was set aside to use as a 'no RT' control. For reverse transcription, 1 μg RNA was used as template in 500 μL RNase-free tubes (Ambion) using iScript (1 μL) plus 4 μL 5X iScript reaction mix (Bio-Rad) and nuclease-free H_2O to 20 μL reaction volume, with incubation in a thermocycler (25 °C for 5 min, 42 °C for 30 min, 85 °C for 5 min, 4 °C indefinitely).

For qPCR, reactions were assembled on ice in MicroAmp Optical 384-Well Reaction Plates (Applied Biosystems, Life Technologies, Foster City, CA). cDNA from iScript reaction was diluted 4-fold in nuclease free H_2O, targeting 4 μL (50 ng) cDNA/reaction. Oligos were diluted by mixing fwd and rev primers (100 μM stocks) 1:1, with further 60.24-fold dilution in nuclease-free H_2O to a working stock concentration of 0.83 μM each oligo. 6 μL diluted primer mix volumes were added to appropriate wells of a 96-well qPCR plate using low-retention tips and multichannel pipet (targeting 250 nM final working concentration in 20 μL qPCR volume). 4 μL diluted cDNA was then added, followed by 10 μL SsoAdvanced Supermix (Bio-Rad) (data collected as technical triplicates). Plates were sealed with clear Microseal B adhesive seals (Bio-Rad), vortexed briefly, then centrifuged 5 min, 1,500 x*g* in a table-top centrifuge. Cycling was performed using 95 °C, 30

sec, [95 °C, 5 sec, 57 °C, 30 sec], cycle 40x, with melt curve analysis in QuantStudio™ Real-Time PCR Software v1.3 on a QuantStudio 6 Flex 384-well instrument (Applied Biosystems). Three loci were used as internal reference controls (*RPL19, GAPDH, HMBS*) for geometric mean normalization, as recommended in (20). Reference controls were verified as unresponsive to dex treatment (RNA-seq, A549, ±100 nM dex, 4 h; Lindsey Pack & Keith Yamamoto, unpublished). Amplicons spanned exon-exon junctions (sequences and details in **Supp. Excel file—Oligonucleotides**, "ST6 qPCR primers"). Primer sets were verified for efficiency on titrated cDNA (reverse-transcribed RNA from A549, ±100 nM dex) and for specificity by melt curve analyses. All qPCR experiments met MIQE standards (21). Primer sequences, efficiencies/amplification factors, and targeted GENCODE transcript IDs available in **Supp. Excel file**.

Fold-change was expressed as ΔΔCT (dex/EtOH), with analysis performed *via* Python pandas data frames and plots generated in GraphPad Prism version 9.0.0 for macOS, GraphPad Software, San Diego, California USA, www.graphpad.com. ΔCT was calculated as $CT_{\text{experimental gene}} - CT_{\text{geometric mean [reference controls]}}$. A549 clone identities for ΔΔ+*86.85 kb* mutants in Fig. 5 are 4-4 G02 (*clone #1a*) and 6-5 F03 (*clone #1b*); ΔΔ-*26.65 kb* mutants are 11-1 D06 (*clone #2a*) and 11-5 G06 (*clone #2b*) (**Supp. Fig. 14**). Clone genotypes were confirmed by PCR-amplification, cloning (Zero Blunt™ TOPO™ PCR Cloning Kit, with One Shot™ TOP10 Chemically Competent *E. coli* cells, Invitrogen), and RCA (Rolling Circle Amplification) sequencing of 5 transformants/clone (ELIM Biopharmaceuticals, Inc., Hayward, CA).

Supplementary References

1. Stenglein,M.D., Jacobson,E.R., Chang,L.-W., Sanders,C., Hawkins,M.G., Guzman,D.S.-M., Drazenovich,T., Dunker,F., Kamaka,E.K., Fisher,D., *et al.* (2015) Widespread recombination, reassortment, and transmission of unbalanced compound viral genotypes in natural arenavirus infections. *PLoS Pathog.*, **11**, e1004900.

2. Altschul,S.F., Gish,W., Miller,W., Myers,E.W. and Lipman,D.J. (1990) Basic local alignment search tool. *J. Mol. Biol.*, **215**, 403–410.

3. Fass,J.N. and Joshi,N.A. (2011) Sickle: A sliding-window, adaptive, quality-based trimming tool for FastQ files (Version 1.33) [Software]. Available at https://github.com/najoshi/sickle.

4. Martin,M. Cutadapt removes adapter sequences from high-throughput sequencing reads. *EMBnetjournal, North America.*

5. Bolger,A.M., Lohse,M. and Usadel,B. (2014) Trimmomatic: a flexible trimmer for Illumina sequence data. *Bioinformatics*, **30**, 2114–2120.

6. Chen,S., Zhou,Y., Chen,Y. and Gu,J. (2018) fastp: an ultra-fast all-in-one FASTQ preprocessor. *Bioinformatics*, **34**, i884–i890.

7. Building a BLAST database with local sequences (2008) Building a BLAST database with local sequences. In *BLAST® Command Line Applications User Manual [Internet].* National Center for Biotechnology Information (US).

8. Brown,J., Pirrung,M. and McCue,L.A. (2017) FQC Dashboard: integrates FastQC results into a web-based, interactive, and extensible FASTQ quality control tool. *Bioinformatics*, **33**, 3137–3139.

9. Bailey,T.L., Boden,M., Buske,F.A., Frith,M., Grant,C.E., Clementi,L., Ren,J., Li,W.W. and Noble,W.S. (2009) MEME SUITE: tools for motif discovery and searching. *Nucleic Acids Res.*, **37**, W202–8.

10. Grant,C.E., Bailey,T.L. and Noble,W.S. (2011) FIMO: scanning for occurrences of a given motif. *Bioinformatics*, **27**, 1017–1018.

11. Khan,A., Fornes,O., Stigliani,A., Gheorghe,M., Castro-Mondragon,J.A., van der Lee,R., Bessy,A., Chèneby,J., Kulkarni,S.R., Tan,G., *et al.* (2018) JASPAR 2018: update of the open-access database of transcription factor binding profiles and its web framework. *Nucleic Acids Res.*, **46**, D1284–D1284.

12. Hubisz,M.J., Pollard,K.S. and Siepel,A. (2011) PHAST and RPHAST: phylogenetic analysis with space/time models. *Brief. Bioinformatics*, **12**, 41–51.

13. Navarro Gonzalez,J., Zweig,A.S., Speir,M.L., Schmelter,D., Rosenbloom,K.R., Raney,B.J., Powell,C.C., Nassar,L.R., Maulding,N.D., Lee,C.M., *et al.* (2020) The UCSC Genome Browser database: 2021 update. *Nucleic Acids Res.*, **12**, 996.

14. Nguyen,N.T.T., Contreras-Moreira,B., Castro-Mondragon,J.A., Santana-Garcia,W., Ossio,R., Robles-Espinoza,C.D., Bahin,M., Collombet,S., Vincens,P., Thieffry,D., *et al.* (2018) RSAT 2018: regulatory sequence analysis tools 20th anniversary. *Nucleic Acids Res.*, **46**, W209–W214.

15. Li,H. (2011) Tabix: fast retrieval of sequence features from generic TAB-delimited files. *Bioinformatics*, **27**, 718–719.

16. D'Ippolito,A.M., McDowell,I.C., Barrera,A., Hong,L.K., Leichter,S.M., Bartelt,L.C., Vockley,C.M., Majoros,W.H., Safi,A., Song,L., *et al.* (2018) Pre-established Chromatin Interactions Mediate the Genomic Response to Glucocorticoids. *Cell Syst*, **7**, 146–160.e7.

17. Chen,S.-H., Masuno,K., Cooper,S.B. and Yamamoto,K.R. (2013) Incoherent feed-forward regulatory logic underpinning glucocorticoid receptor action. *Proc. Natl. Acad. Sci. U.S.A.*, **110**, 1964–1969.

18. Jiang,F. and Doudna,J.A. (2017) CRISPR-Cas9 Structures and Mechanisms. *Annu Rev Biophys*, **46**, 505–529.

19. Doench,J.G., Fusi,N., Sullender,M., Hegde,M., Vaimberg,E.W., Donovan,K.F., Smith,I., Tothova,Z., Wilen,C., Orchard,R., *et al.* (2016) Optimized sgRNA design to maximize activity and minimize off-target effects of CRISPR-Cas9. *Nat. Biotechnol.*, **34**, 184–191.

20. Vandesompele,J., De Preter,K., Pattyn,F., Poppe,B., Van Roy,N., De Paepe,A. and Speleman,F. (2002) Accurate normalization of real-time quantitative RT-PCR data by geometric averaging of multiple internal control genes. **3**, RESEARCH0034.

21. Bustin,S.A., Benes,V., Garson,J.A., Hellemans,J., Huggett,J., Kubista,M., Mueller,R., Nolan,T., Pfaffl,M.W., Shipley,G.L., *et al.* (2009) The MIQE guidelines: minimum information for publication of quantitative real-time PCR experiments. *Clin Chem*, **55**, 611–622.
